# Fate of PFAS Through a Biosolids Drum Dryer With Regenerative Thermal Oxidizer Emissions Control

**DOI:** 10.1002/wer.70149

**Published:** 2025-10-20

**Authors:** John J. Ross, Alex Seidel, Embrey Bronstad, Farokh Kakar, Mary Lou Romero, Martha J. M. Wells, Lloyd J. Winchell, Katherine Y. Bell, Don Song

**Affiliations:** ^1^ Brown and Caldwell Walnut Creek California USA; ^2^ EnviroChem Services Cookeville Tennessee USA; ^3^ Synagro Technologies Baltimore Maryland USA

**Keywords:** gas‐phase PFAS, PFAS, PFAS transformation, thermal drying, thermal processes

## Abstract

Thermal dryers are commonly used for biosolids volume reduction and stabilization, but the fate of PFAS in these systems is not well understood. Triplicate samples from a full‐scale rotary drum dryer were evaluated, including gas‐phase emissions before and after a regenerative thermal oxidizer used for emissions control. Substantial PFAS removal between the feed and dried solids (60.0% on a total molar basis for targeted PFAS) was observed. PFAS did not accumulate in the drain water from wet scrubbers treating dryer exhaust. The scrubbed exhaust contained six orders of magnitude less PFAS than the dewatered feed solids on a total molar mass basis. The destruction or removal efficiency of PFAS across the RTO was complicated by contaminant‐prone and thermally unstable HFPO‐DA, achieving 93.5% when including the suspect compound and 99.3% when not. Further research is needed to identify potential transformation mechanisms and products to close the PFAS balance across drying processes.

## Summary

- When targeted PFAS values for the dryer inputs and outputs are considered, it is likely that a considerable amount of PFAS is transferring from dewatered solids to the gas phase in the dryer (60% of targeted analytes).
- Only a small fraction of targeted PFAS were detected in the gas phase (2.37 × 10^−4^% of targeted analytes found in the thickened solids samples on a molar basis), which suggests PFAS may be transforming within the dryer or within the exhaust recycle loop through the furnace.
- The PFAS destruction and removal (DRE) efficiency of the regenerative thermal oxidizer emission control device (reported as > 90%) is called into question due to HFPO‐DA complications. If the HFPO‐DA values are disregarded, the RTO achieves a PFAS DRE of 99.3% on a sum molar mass basis.

## Graphical abstract

Reportable levels of PFAS and organoflourine compounds were found to be reduced in the outputs of a biosolids drum dryer compared to its inputs (60% of targeted analytes). A small fraction of targeted PFAS were detected in the gas phase and the emissions control system achieved > 90% PFAS destruction or removal.

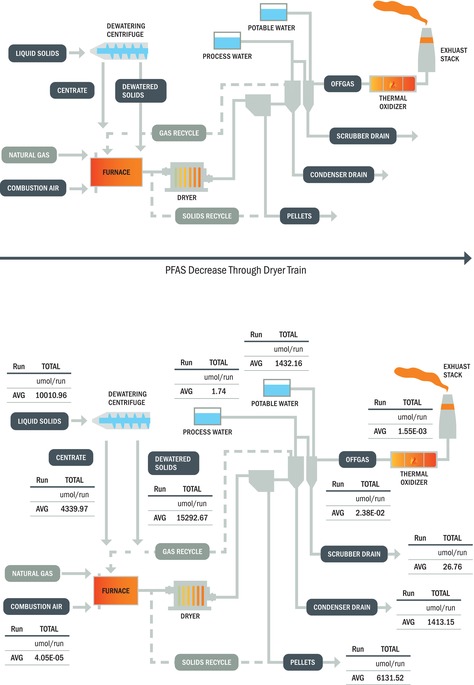

## 1 Introduction

Perfluoroalkyl and polyfluoroalkyl substances (PFAS) have become an important environmental contaminant of concern, particularly for water resource recovery facilities (WRRF) that face increasing scrutiny regarding the disposition of wastewater solids (biosolids when stabilized). In the United States, concerns regarding PFAS release to soils (Munoz et al. 2022), groundwater (Pepper et al. 2023; Pepper et al. 2021) and agricultural crops (Ghisi et al. 2019) from land application have increased scrutiny on traditional solids management practices. This study was developed and commissioned by Synagro Technologies, a biosolids processing and recycling company, to better understand the fate of PFAS through one of the primary biosolids processing technologies: thermal drying. Specifically, this study was designed to analyze PFAS at all inputs and outputs of a rotary drum dryer, which is commonly used for thermal drying at large‐scale WRRFs.

PFAS management solutions for biosolids is an increasingly significant topic in the wastewater industry, with thermal processes garnering attention as a promising treatment method (Winchell et al. 2021, 2024; Winchell, Ross, et al. 2022). While gaining interest, research has focused primarily on high‐temperature (> 1000°C) processing for PFAS destruction or removal. Because PFAS contain strong carbon‐fluorine bonds, they are known to degrade only at high temperatures, and thermal oxidation has been permitted as a best available control technology for gas‐phase PFAS emissions (Blotevogel et al. 2023; Winchell et al. 2021; Focus Environmental Inc. 2020; BARR 2022; Shields et al. 2023; Vargette et al. 2023).

Thermal drying operates at lower temperatures (average of 355°C in this study) than pyrolysis, incineration, or thermal oxidation. It also treats biosolids to meet US Environmental Protection Agency (USEPA) 40 Code of Federal Regulations (CFR) Part 503 Class A requirements, thereby increasing the diversity of final disposition outlets (USEPA 1993). The dual benefits of volume reduction and increased options for product disposition provide WRRFs with greater control over programmatic risks and operational costs associated with regulatory uncertainty over PFAS. Thermal dryers use a variety of heating media (hot water, steam, and thermal oil radiators; direct‐fired process gas; and direct contact with heated metal paddles, augers, or reactor jacketing) over a range of temperatures from 120°C to 650°C (WEF 2018; Williams 2023). Biosolids drying is typically designed to meet Class A pathogen reduction requirements per 40 CFR Part 503, where 10%–25% moisture is retained (USEPA 1993). If heat is evenly applied during drying, temperatures may be maintained < 100°C due to continuous evaporation. Additionally, a slight negative pressure is typically used to minimize the release of dust or odors (WEF 2014).

The fate of PFAS through biosolids dryers has not been extensively studied. Lazcano et al. (2019) tested Class B biosolids in a rotary drum dryer for 45 min at temperatures between 480°C and 650°C. Researchers reported that PFAS (specifically perfluoroalkyl acids [PFAA]) concentrations increased by 53%, largely due to an increase in perfluorohexanoic acid (PFHxA) and inferred that PFAS precursors were transformed during the drying process.

In a separate study, biosolids drying at 85°C–90°C for 20 min resulted in significant formation of perfluoropentanoic acid (PFPeA), pointing to hydrolysis of precursors (Lakshminarasimman et al. 2021). In this study, samples were collected twice (1 year apart over 3 consecutive days) and sent in chilled containers overnight to the laboratory for processing.

A third study found that rotary drum drying decreased the total sum of selected PFAS concentrations up to 31% (Rahman et al. 2025). Researchers hypothesized that PFAS volatilization drove the compounds into the vapor phase, which left lower concentrations in the dried solids fraction. Rahman et al. found this effect was more pronounced at higher drying temperatures; however, specific sampling methodology for the samples in the Rahman et al. study was unavailable.

A fourth study by McNamara et al. (2025) compared the fate of selected targeted PFAS analytes in biosolids through an unspecified full‐ and laboratory‐scale dryer. Pre‐ and post‐drying samples were collected on site from the full‐scale dryer. Additionally, solids samples from three WRRFs and the unspecified WRRF with a full‐scale dryer were processed through a laboratory‐scale oven operating at 105°C. The study found that both full‐ and laboratory‐scale drying significantly reduced the amount of targeted PFAS analytes in the final dried solids by 84.9% and 81.9%, respectively. In that study, triplicate samples were stored at 4°C for < 7 days before analyses.

These limited dryer studies have centered on rotary drum drying, the most common technology for biosolids drying at large WRRFs, because of their higher operating temperatures and higher throughput compared to other technologies (Girovich 1996). In rotary drum dryers, material cascades through a horizontally oriented rotating drum and is exposed to a continuous flow of hot gas and air (400°C–650°C supply). Dryer exhaust and dried product are passed through a gas/solids separator, and a portion of dried product is classified and returned to condition feed solids while a smaller fraction meeting the desired final characteristics is transferred for loadout. Dried solids recycling increases the solids residence time within a rotary drum dryer compared to other biosolids drying technologies (e.g., belt and indirect). In modern drum dryers, most of the air/gas stream (approximately 60%–80%) is also recycled back to the dryer furnace after moisture removal to increase thermal efficiency and maintain an inert atmosphere. Process gas not recycled is sent to emissions treatment, typically comprised of a Venturi scrubber and regenerative thermal oxidizer (RTO) (WEF 2018). The potential exists for PFAS to volatilize from biosolids into the off‐gas, where PFAS could be exposed to high temperatures in the recycle to the furnace (400°C–650°C for at least 1 s residence time for the gas), captured in the emissions scrubbing system and returned to the WRRF, or be submitted to high temperatures (typically at least 815°C for 0.5 s) in the RTO (WEF 2018). While little is known about the fate of PFAS through drying systems, there is a substantial body of evidence that suggests PFAS will be destroyed or degraded under the furnace and RTO operating conditions (Winchell et al. 2021; Xiao et al. 2020; Longendyke et al. 2022; Vargette et al. 2023). PFAS volatilization data could be compared to the environment throughout the dryer (again, less than 100°C due to evaporative cooling and negative 248.21 Pa), to elucidate whether PFAS volatilization is hampered by the few empirically derived Henry's Law constants for these compounds (Wang et al. 2022). Although partitioning data are being collected to support fate and risk analyses (Endo et al. 2023; Abusallout et al. 2022; Kutsuna and Hori 2008), the robustness of the database relative to the number of PFAS compounds, as well as the influence of matrices on which PFAS may be adsorbed, is an area of research that merits continued and further investigation.

In short, the number of dryers used in WRRFs is anticipated to increase considering regulatory and economic pressures related to PFAS compliance. This emphasizes the question regarding the fate of PFAS through dryers and their emissions control technologies, specifically RTOs. To the authors' knowledge, no peer‐reviewed research is available on the fate of PFAS through full‐scale biosolids rotary drum dryers that also examine exhaust emissions. This manuscript documents PFAS sampling and analysis of all inputs and outputs of a rotary drum dryer, including emissions prior to and after the RTO.

## 2 Methodology

Synagro Technologies commissioned this project at one of its full‐scale dewatering and rotary drum drying facilities in May 2023. The dryer evaluated is representative of the drum drying technology used at 14 Synagro drum drying facilities in North America. The test site operates on a continuous supply of anaerobically digested thickened solids. The sampling event consisted of triplicate test runs, each conducted for 4 h.

### 2.1 Sampling Site

The study facility includes centrifuge dewatering and a conventional rotary drum dryer equipped with a condenser, Venturi scrubber, and RTO (see also Section S1). All major inputs and outputs are shown on Figure S1 using the following numbering: (1) thickened solids, (2) dewatering centrate or liquid return, (3) dewatered solids, (4) furnace combustion air, (5) dried solids, (6a) treated WRRF effluent or process water Venturi scrubber supply, (6b) potable water Venturi scrubber supply, (7) condenser drain, (8) Venturi scrubber drain, (9) dryer process gas exhaust or off‐gas, and (10) RTO flue gas or exhaust stack.

Thickened solids fed to the facility represent both a solid‐ and liquid‐phase PFAS input. Thickened solids were conditioned with polymer ahead of dewatering and, notably, according to D. Song, the polymer manufacturer noted that no PFAS were used in its manufacturing (personal communication, April 2022). The polymer, as well as natural gas used in the dryer furnace and RTO, was assumed to be free of measurable PFAS. The dewatering process separates 90%–95% of the feed sludge liquid for return to the WRRF and transfers dewatered solids to the dryer. In the dryer, 95%–99% of the remaining water in the dewatered solids is evaporated, and dried solids are collected as dried solids or pellets.

Both potable and process (treated WRRF effluent) cooling water streams were fed to the scrubbing system used to condition the dryer exhaust (condenser and Venturi scrubber). Potable and process water can contain measurable PFAS levels (Lenka et al. 2021; Tavasoli et al. 2021; Winchell, Wells, et al. 2022). The condenser and Venturi scrubber drains were sampled as potential PFAS sinks that could result from gas–liquid transfer in the scrubbing system, or conversely, potential sources of PFAS release through volatilization in the scrubbing system. Dryer exhaust was sampled after the Venturi scrubber and after the RTO. Neither the dried solids recycle nor the dryer exhaust recycle was sampled, given that the focus of the study was on evaluating PFAS inputs and outputs to the system.

### 2.2 General Analytical Approach

The analytical plan was developed early in 2023 as PFAS analytical methods for biosolids were under development. Thus, the project used the polar PFAS analytical workflow developed by Winchell et al. (2024) to track the PFAS profile across solid, aqueous, and gaseous matrices. A summary of the samples collected and the sampling methods used in this study is presented in Table 1. Note that Eurofins analyzed solid and liquid samples at its Lancaster Laboratories Environment Testing facility in Pennsylvania; stack emission samples were analyzed at Eurofins' Knoxville Laboratories in Tennessee.

**TABLE 1:** Summary of study sample analyses.

Sample	Method	Polar targeted (gas)	Polar targeted (liquid)	Polar targeted (solid)	AOF (polar)	Additional samples
Thickened solids	Modified SW846/537	—	3	3	6	—
Centrate	Modified SW846/537	—	3	3	6	—
Dewatered solids	Modified SW846/537	—	—	3	3	1 solid ^a^
Combustion air	Modified OTM‐45	3	—	—	3	—
Dried solids	Modified SW846/537	—	—	3	3	1 solid ^a^
Cooling water supply (potable)	Modified SW846/537	—	3	—	3	1 liquid ^b^
Cooling water supply (process)	Modified SW846/537	—	3		3	1 liquid ^b^
Condenser drain	Modified SW846/537	—	3		3	2 liquid ^a^, ^b^
Scrubber drain	Modified SW846/537	—	3		3	1 liquid ^b^
RTO inlet	OTM‐45	3	—	—	—	1 gas ^b^
RTO exhaust	OTM‐45	3	—	—	—	—

^a^ Field duplicate.

^b^ Extra run.

#### 2.2.1 Targeted PFAS Analysis

At the time of this study, USEPA Method 1633 (USEPA 2024b) for analysis of polar PFAS in aqueous, solid, biosolids, and tissue samples by liquid chromatography tandem mass spectrometry (LC–MS/MS) was not finalized. Therefore, PFAS in solid and aqueous samples were analyzed using Eurofins Laboratories' proprietary standard operating procedures (SOP) based on Method SW846 (USEPA 2022) and Method 537 (modified) adapted from USEPA Method 537.1 (Shoemaker and Tettenhorst 2020).

Gaseous samples were collected according to the Other Test Method 45 (OTM‐45) Measurement of Selected Per‐ and Polyfluorinated Alkyl Substances from Stationary Sources (USEPA 2021). OTM‐45 measures semi‐volatile and particulate‐bound PFAS in stationary sources with a target analyte list analogous to Method 1633. OTM‐45 isokinetically extracts gas from the stationary emissions source and passes it through a particulate filter (part of analytical fraction 1), XAD resin tube (fraction 2), a series of water‐filled impingers (fraction 3), and a final breakthrough XAD‐2 resin tube (fraction 4). The OTM‐45 method and analytical sample fractions are described in Section S2. Since the study was conducted, USEPA has introduced Other Test Method 50 (OTM‐50) Sampling and Analysis of Volatile Fluorinated Compounds to measure volatile fluorinated compounds (VFC), indicative of incomplete decomposition of PFAS from thermal treatment technologies (USEPA 2025). Consequently, a limitation of the study is that OTM‐50 was not available to detect gas‐phase VFCs.

#### 2.2.2 Gross Organoflourine Analysis

Given that the non‐ and semi‐volatile polar PFAS targeted with the aforementioned analytical techniques represent a small fraction of the total number of PFAS present, the project also measured gross organofluorine compounds in liquid and solid samples using total organic fluorine (TOF) methods with combustion ion chromatography (CIC). The USEPA Method 1621 (USEPA 2024b) for Determination of Adsorbable Organic Fluorine (AOF) in Aqueous Matrices by CIC was not finalized until after sampling, and samples for this study were processed with proprietary analyses applied by Eurofins. TOF generically refers to AOF for liquids or extractable organic fluorine (EOF) for solids, separately applied depending on the sample matrix.

### 2.3 Sampling, Sample Preparation, and Analysis

Alliance Source Testing (AST) conducted onsite sample collection and provided specialized sampling equipment, including a stack emissions testing apparatus. Eurofins provided clean bottleware for the sampling event. Following sampling, Eurofins staff packed samples on ice and managed transportation to the laboratory.

#### 2.3.1 Process Air Emissions (Dryer Exhaust and Stack)

Two trains of the source air emissions sample apparatus were assembled according to OTM‐45 for sampling the dryer exhaust and RTO exhaust (USEPA 2021). Source air analytics are reported for the four fractions from the sampling train—front half and filter, back half and XAD‐2 resin tube, impinger condensate, and breakthrough XAD‐2 resin—collected from the sampling train. Analytical results of each fraction are converted to molar mass and summed to represent the sample PFAS levels.

Given the lack of detection of non‐polar PFAS in source emissions of biosolids thermal processes (i.e., sewage sludge incineration) documented by Winchell et al. (2024), the sequential extraction used in this study was not included. Surrogate and isotope dilution standards were spiked into samples before laboratory extraction.

#### 2.3.2 Combustion Air

Combustion air intake samples were collected using an XAD‐2 resin tube and using the same isokinetic extraction and sample preparation as used for the breakthrough XAD‐2 resin tube in the source air emissions samples described previously.

#### 2.3.3 Solid‐Phase Samples

Dewatered and dried solids composite samples were collected in two 4‐oz (113‐g) high‐density polyethylene (HDPE) containers: one for targeted PFAS analysis and EOF, and the other for total solids and volatile solids content. Samples were composited over the 4‐h duration of each test run in each jar. Equal volumes (approximately 10% of the jar volume) were collected at the start of the sample run and every 30 min thereafter, then combined and homogenized. After each run, sample bottles were packed on ice and, after the test event, transported on ice to the laboratory for analyses.

Neither dewatered solids nor dried solids contained filterable liquid. Surrogate and isotope dilution standards were spiked into samples prior to extraction. Raw samples were subjected to Eurofins proprietary extraction procedure, Method 537 (modified), that is, polar, basic extraction using a 0.4% potassium hydroxide (KOH) and methanol (CH_3_OH) solution subjected to shaking and sonication with final pH adjustment. The extract was cleaned using solid‐phase extraction/weak anion exchange (SPE/WAX). PFAS analytes were eluted using a 0.3% ammonium hydroxide (NH_4_OH) and CH_3_OH solution. The EOF sample was processed separately with calcium hydroxide (Ca (OH)_2_) to precipitate fluoride and processed for TOF.

#### 2.3.4 Liquid‐Phase Samples

Composite samples of thickened solids, scrubbing system supply, and drain streams were collected every 30 min and composited in two 250‐mL HDPE containers (for targeted PFAS analysis and AOF) and one 1‐L HDPE container (for total suspended solids and volatile solids). Equal volumes (approximately 10% of the bottle volume) were collected at the start of the sample run and every 30 min thereafter, then combined and homogenized. After each run, sample bottles were packed with ice and, after the test event, transported on ice to the laboratory for analyses.

Samples were filtered to separate the liquid and solid phases; surrogate and isotope dilution standards were spiked into samples prior to extraction. Filtered liquid from the raw samples was processed by SPE/WAX, and the analyte was eluted using 0.3% NH_4_OH and CH_3_OH solution. An aliquot of the resulting polar extract was analyzed by polar‐targeted analysis. A second aliquot of the extract was further processed with Ca (OH)_2_ to remove fluoride prior to TOF analysis.

#### 2.3.5 Analytical Methods

The following sections identify the methods used in this research. Further description of the quality control measures is provided in Sections 3.3.5 and S2.

##### 2.3.5.1 Targeted—Polar

Quantitative targeted polar analyses used known reference standards based on standard regulatory methods (Shoemaker and Tettenhorst 2020) and extended methods established by the analytical vendor referred to here as Method 537 (modified). The list of PFAS examined is presented in Table S3. Refer to this table for the names and acronyms of individual PFAS compounds and the PFAS families to which they belong, hereafter referred to by their acronyms.

##### 2.3.5.2 TOF

TOF analysis was conducted for this study. Briefly, PFAS were adsorbed, most often on activated carbon, which was then subjected to combustion to release the fluoride ion (F−) (HEPA 2020; Trojanowicz and Koc 2013; Mills et al. 2020). Removing inorganic F− allowed only organic F− to be measured. Combustion at 900°C–1000°C releases fluorine, after which fluoride concentration can be determined via ion chromatography. The combined process is termed CIC (Eurofins 2018; Trojanowicz and Koc 2013). Organic fluorine analyses are generically referred to as TOF unless reference material from elsewhere is noted.

To determine the percent TOF explained by targeted PFAS, the TOF data and the PFAS data were assessed separately. This assessment was done for fluoride and PFAS, respectively, using quality assurance/quality control (QA/QC) processing as described in Section 2.4. Second, the fluoride concentration of the method blank was subtracted from the TOF in the sample to estimate the fluoride attributable to the sample itself. Subtracting method blanks was necessary because surrogate and isotope dilution standards were spiked into samples and method blanks before extraction. The blank‐subtracted TOF fluoride concentration was converted to moles. Next, the fluorine equivalent in the targeted PFAS was calculated by (1) converting the mass of each PFAS detected into moles using the individual PFAS molecular weights, (2) multiplying the number of fluorine atoms in the molecular formula of each PFAS compound detected by its molar concentration to determine the moles of fluorine present in targeted analyses, and (3) summing the moles of all fluorine determined for PFAS in the targeted sample. Finally, the fluorine equivalent of the targeted PFAS was compared to the actual organofluorine measured by calculating the percent TOF explained by targeted PFAS.

##### 2.3.5.3 Solids Characteristics

The solid‐phase samples and solid‐phase extracts from liquid samples were characterized as described in Section S2.5. Solid‐phase samples were analyzed for moisture using Standard Method (SM) 2540G (APHA 2017a) and total solids and volatile solids using SM 2540G. Total suspended solids were measured for the Venturi scrubber supply and drain samples using SM 2540D (APHA 2017b).

### 2.4 Data Quality Standards

Data were assessed for quality as outlined in Winchell et al. (2024) using (1) the QA/QC data provided by the analytical laboratory, (2) the Brown and Caldwell data verification and validation guidelines for reporting general chemistry parameters, (3) guidance documents from USEPA (2002) and Interstate Technology & Regulatory Council (ITRC, 2022a and 2022b), (4) though not promulgated at the time, guidance from USEPA Draft Method 1633 (US EPA 2024b) and USEPA Draft Method 1621 (US EPA 2024a) was considered, and (5) the experience/expertise of the project research team.

The data quality review process proceeded through the stages documented by ITRC (2022a). Assessment of data usability was the final step after verification, validation, and establishment of data quality. Eurofins provided extensive quality control information in the form of data qualifiers for the analyses reported. The research team reviewed data to verify and validate results to assess the overall data quality. Finally, the data qualifiers were interpreted by the team to assess data usability. A list of qualifiers and definitions encountered, along with their interpretation for the determination of data usability in this project, is presented in Table S5.

Among the most important data quality parameters is the sensitivity of the analysis. Numerous terms and acronyms have evolved in the literature to describe “detection” and “quantitation” limits (USEPA 1995; ITRC 2022a). In these data, the terms minimum detection limit (MDL) and reporting limit (RL) are used to report the “detection” and “quantitation” limits, respectively. The MDL is defined as the minimum measured concentration of a substance that can be reported with 99% confidence that the measured concentration is distinguishable from method blank results (USEPA 2017). The RL is the lowest non‐zero calibration point in the calibration curve for each analyte. Because of varying properties among samples, i.e., sample size, matrix effects, and dilutions made during analysis, the RL can vary from sample to sample and analyte to analyte (ITRC 2022b). The RL values for each analyte in each sample are provided in Tables S18–S23.

The “J” qualifier represents an estimated analytical result between the MDL and the RL. The project team determined that although the numerical value having a J qualifier as reported by the laboratory was a valid indicator of data “quality,” the J qualifier represents an estimation outside the calibration curve; therefore, its “usability” is unfit. Data having J qualifiers, regardless of other qualifiers listed for the sample, were not reported numerically but were reported as “J.” No numerical values were reported for the data qualifiers I, CI, *+, and *1; the numerical value was replaced by the data qualifier itself to alert the data user that the result could not be confirmed.

The “B” qualifier was assessed differently for the chromatographic quantitative data versus the surrogate qualitative data based on the not‐yet‐promulgated guidance from USEPA Draft Method 1633 (USEPA 2024b) and Method 1621 (USEPA 2024a). This is detailed in Section S2.7. For the B data qualifier associated with the chromatographic quantitative data, no numerical values were reported; the numerical value was replaced by the data qualifier itself to alert the data user that the result could not be confirmed. For the B data qualifier associated with the surrogate qualitative data in TOF analyses, the numerical value was reported accompanied by a B superscript. For the TOF samples, samples and method blanks contain isotope dilution analysis and surrogate standards necessitating the subtraction of the method blank from the sample (USEPA 2024a). Judgment criteria on which data was fundamentally deemed usable are presented in Table S5 while Section S2.7 contains additional details on the screening approach.

### 2.5 Dryer Operating Conditions

**FIGURE 1:**
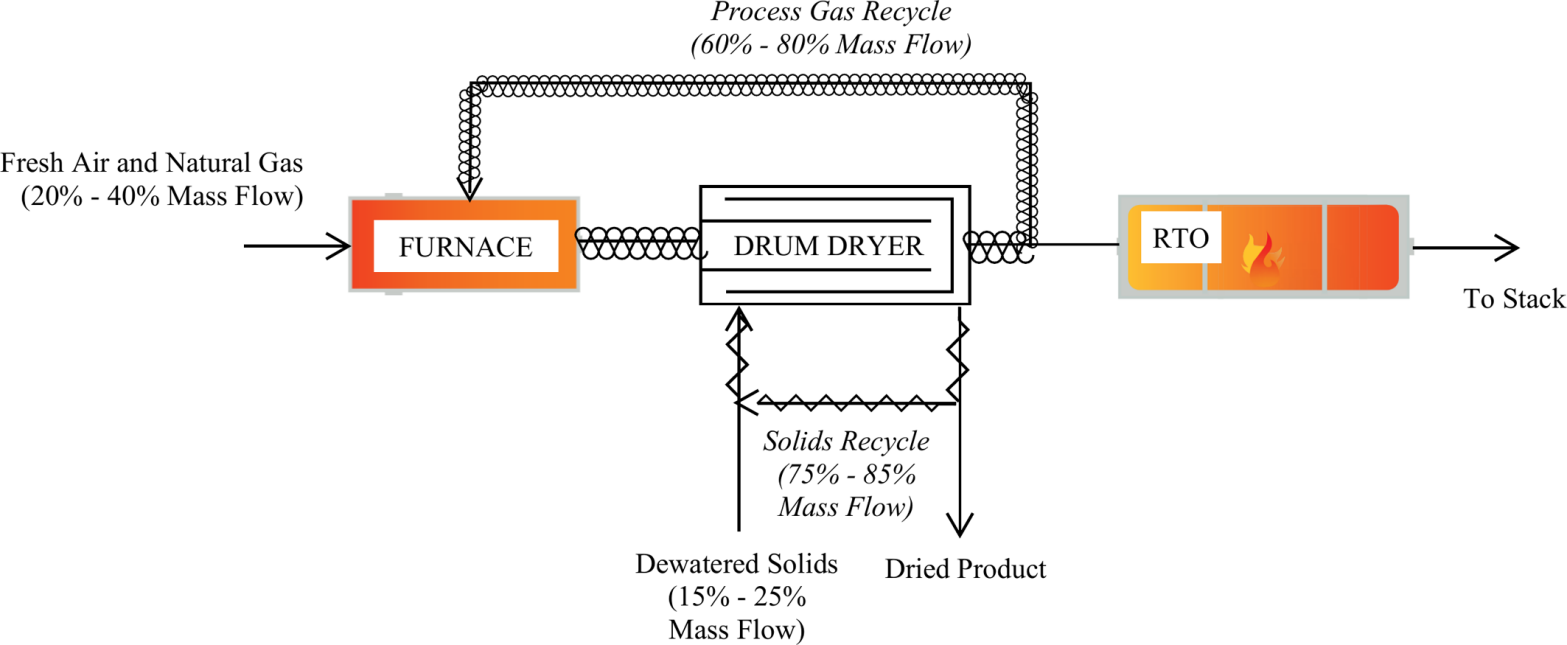
Schematic of process air and solids inputs into a biosolids rotary drum dryer system.

A schematic representation of the primary inputs and outputs to the dryer system, with the relative contribution of internal recycle streams, is presented in Figure 1. More details on the design rates for recycle flows are presented in Section S3.

The test site RTO is a ceramic‐bed type used to oxidize volatile organic compounds for odor control. The RTO passes process gases through an active combustion chamber at a maximum flow rate and with a continuously monitored dryer supply temperature to achieve a uniform operating temperature and turbulence over a minimum residence time. The RTO has a minimum temperature setpoint of 815°C with a minimum residence time of 0.5 s for all process gases. During the test event, the temperature in the two combustion chambers of the RTO ranged between 835°C and 856°C.

## 3 Results

Data presented here have passed the quality review process and are deemed usable for the purposes of this study. Quality control markers found during this process are presented in place of the data in question. An explanation of the data qualifiers is described previously and presented in Table S5.

Overall, 49 PFAS were analyzed; however, not all PFAS analytes were evaluated for each sample. Each analyte tested corresponded to specific methods, which in some instances were not applicable to the entirety of the original suite of PFAS compounds. Liquid and solid samples were not analyzed for the fluorotelomer carboxylic acids, fluorotelomer unsaturated carboxylic acids, three of the five perfluoroether and polyfluoroether carboxylic acids, as well as PFPA, PFEESA/PES, or NMeFOSA analyzed in the gas samples. PFPA and NMeFOSE were analyzed in liquid and solid samples, but not in gas samples.

A comparison of long‐ and short‐chain PFAS compounds is presented for applicable samples. Here, long‐chain PFAS compounds were defined as any PFAS compound with a fully fluorinated chain of eight or more carbons, with exceptions for two PFAS families. Perfluoroalkyl carboxylic acids with chains of seven or more, and perfluoroalkyl sulfonic acids with chains of six or more fully fluorinated carbons, were considered long‐chain PFAS as well (Buck et al. 2011). All other PFAS compounds were considered short‐chain PFAS.

As mentioned, TOF analyses were conducted for this study. Although TOF methods do not distinguish between PFAS and non‐PFAS organofluorine compounds such as pharmaceuticals and pesticides (Han et al. 2021), they can serve as a useful reference when considered in parallel with changes in targeted PFAS profiles. For example, if targeted PFAS levels decrease in the solids‐phase samples but TOF levels do not, then findings may suggest PFAS could be transformed but retained in solids samples. TOF was not performed for the OTM‐45 gaseous samples, as OTM‐45 is configured for semi‐volatile polar PFAS and would not be likely to detect the precursor or transformation products that would represent much of the TOF mass (Winchell et al. 2024).

Comparison of the percent total fluorine‐based compounds explained by targeted PFAS was reported. TOF analyses (either AOF or EOF) and targeted PFAS analyses measure different chemical entities using different types of instrumentation, yielding dissimilarities in anticipated RLs. TOF analyses apply CIC to measure fluoride converted from the organic fluorine present. Polar targeted analyses determine the presence of specific PFAS using LC–MS/MS. The fluoride versus PFAS concentrations of RLs for these analytical methods can be separated by three or four orders of magnitude (powers of 10), that is, micrograms per liter (μg·L^−1^) for AOF (liquids) compared to nanograms per liter (ng·L^−1^) for PFAS in water, or micrograms per gram (μg·g^−1^) for EOF (solids) compared to nanograms per gram (ng·g^−1^) for PFAS in solids. Additionally, the TOF RLs were observed to be sample‐matrix dependent. For example, in more contaminated sources, the AOF RL for the thickened solids was 5–10 times higher than the cooling water supply depending on the source, and the EOF RL for dewatered solids was approximately five times higher than that for the dried solids, making comparisons challenging.

### 3.1 Dryer Inputs: PFAS and Fluorine‐Based Compounds

Reportable PFAS measurements for inputs to the dewatering and drying system are presented in Table 2. Source data, including numerical data not reported in Table 2, are provided in Table S10. An interpretation of these results is provided for each sample point.

**TABLE 2:** Dryer system input samples—reportable results.

		Thickened solids (liquid fraction)		Thickened solids (solid fraction)		Dryer combustion air		Scrubber system cooling water supply (process)		Scrubber system cooling water supply (potable)	
		Concentration	Emission	Concentration	Emission	Concentration	Emission	Concentration	Emission	Concentration	Emission
	Units	ng/L	mg/run	ng/g	mg/run	ng/sample	mg/run	ng/L	mg/run	ng/L	mg/run
PFAS family	Acronym	*(R1/R2/R3)*	*(R1/R2/R3)*	*(R1/R2/R3)*	*(R1/R2/R3)*	*(R1/R2/R3)*	*(R1/R2/R3)*	*(R1/R2/R3/R4)*	*(R1/R2/R3/R4)*	*(R1/R2/R3/R4)*	*(R1/R2/R3/R4)*
Fluorotelomer sulfonic acids	8:2 FTS	ND (RL: 300)/ND (RL: 300)/ND (RL: 300)	—/—/—	ND/ND/ND	—/—/—	< RL (J)/< RL (J)/< RL (J)	—/—/—	ND/ND/ND/ND	—/—/—/Q	ND/ND/ND/ND	—/—/—/Q
	10:2 FTS	ND (RL: 500)/ND (RL: 500)/ND (RL: 500)	—/—/—	ND/ND/< RL (J)	—/—/—	ND/ND/ND	—/—/—	ND/ND/ND/ND	—/—/—/Q	ND/ND/ND/ND	—/—/—/Q
Perfluoroalkyl carboxylic acids	PFBA	ND (RL: 500)/ND (RL: 500)/ND (RL: 500)	—/—/—	ND/ND/ND	—/—/—	CI/CI/CI	—/—/—	7/6.4/6.9/7.4	3.24/2.95/3.17/Q	ND/ND/ND/ND	—/—/—/Q
	PFPeA	ND (RL: 200)/ND (RL: 200)/ND (RL: 200)	—/—/—	ND/ND/ND	—/—/—	ND/ND/ND	—/—/—	21/21/22/23	9.72/9.68/10.1/Q	< RL (J)/< RL (J)/< RL (J)/< RL (J)	—/—/—/—
	PFHxA	ND (RL: 200)/ND (RL: 200)/ND (RL: 200)	—/—/—	ND/ND/< RL (J)	—/—/—	1.27/1.19/1.01	1.27E−06/1.19E−06/1.01E−06	29/25/26/29	13.42/11.52/11.94/Q	< RL (J)/< RL (J)/< RL (J)/J I	—/—/—/Q
	PFHpA	ND (RL: 200)/ND (RL: 200)/ND (RL: 200)	—/—/—	ND/ND/ND	—/—/—	ND/ND/ND	—/—/—	8/7.5/7.7/6.9	3.7/3.46/3.53/Q	< RL (J)/< RL (J)/J I/< RL (J)	—/—/Q/—
	PFOA	ND (RL: 200)/< RL (RL: 200) (J)/< RL (RL: 200) (J)	—/—/—	< RL (J)/< RL (J)/< RL (J)	—/—/—	ND/< RL (J)/ND	—/—/—	16/15/15/15	7.4/6.91/6.89/Q	< RL (J)/< RL (J)/< RL (J)/< RL (J)	—/—/—/—
	PFNA	ND (RL: 200)/ND (RL: 200)/ND (RL: 200)	—/—/—	ND/ND/ND	—/—/—	ND/ND/ND	—/—/—	< 2.3 (J)/< 2.1 (J)/< RL (J)/< RL (J)	Q/Q/—/—	< RL (J)/ND/ND/ND	—/—/—/Q
	PFDA	ND (RL: 200)/ND (RL: 200)/ND (RL: 200)	—/—/—	< RL (J)/< RL (J)/22	—/—/149.38	ND/ND/ND	—/—/—	2.9/< 2.1 (J)/2/< RL (J)	1.34/Q/0.92/—	ND/ND/ND/ND	—/—/—/Q
	PFDoA	ND (RL: 200)/ND (RL: 200)/ND (RL: 200)	—/—/—	ND/ND/< RL (J)	—/—/—	ND/ND/ND	—/—/—	< 2.3 (J)/ND/< RL (J)/ND	Q/—/—/Q	ND/ND/ND/ND	—/—/—/Q
Perfluoroalkyl sulfonic acids	PFBS	ND (RL: 200)/ND (RL: 200)/ND (RL: 200)	—/—/—	ND/ND/ND	—/—/—	ND/ND/ND	—/—/—	4.8/4.7/4/4.9	2.22/2.17/1.84/Q	< RL (J)/< RL (J)/< RL (J)/< RL (J)	—/—/—/—
	PFPeS	ND (RL: 200)/ND (RL: 200)/ND (RL: 200)	—/—/—	ND/ND/ND	—/—/—	ND/ND/ND	—/—/—	ND/< 2.1 (J)/< RL (J)/ND	—/Q/—/Q	ND/ND/ND/ND	—/—/—/Q
	PFHxS	ND (RL: 200)/ND (RL: 200)/ND (RL: 200)	—/—/—	ND/ND/ND	—/—/—	ND/ND/ND	—/—/—	2.7/2.8/2.9/3.2	1.25/1.29/1.33/Q	< RL (J)/< RL (J)/< RL (J)/< RL (J)	—/—/—/—
	PFOS	ND (RL: 200)/ND (RL: 200)/ND (RL: 200)	—/—/—	23/28/49	178.92/209.5/332.71	ND/ND/ND	—/—/—	4.2/4.3/4.3/4.1	1.94/1.98/1.97/Q	< RL (J)/< RL (J)/< RL (J)/< RL (J)	—/—/—/—
	PFDS	ND (RL: 200)/ND (RL: 200)/ND (RL: 200)	—/—/—	ND/ND/< RL (J)	—/—/—	*−/*−/*−	—/—/—	ND/ND/ND/ND	—/—/—/Q	ND/ND/ND/ND	—/—/—/Q
	PFDoS	ND (RL: 300)/ND (RL: 300)/ND (RL: 300)	—/—/—	ND/ND/< RL (J)	—/—/—	*− *1/*− *1/*− *1	—/—/—	ND/ND/ND/ND	—/—/—/Q	ND/ND/ND/ND	—/—/—/Q
Perfluorooctane sulfonamides	FOSA	ND (RL: 200)/ND (RL: 200)/ND (RL: 200)	—/—/—	ND/ND/ND	—/—/—	ND/ND/ND	—/—/—	4.6/< 2.1 (J)/< RL (J)/< RL (J)	2.13/Q/—/—	7.1/4.6/4.8/4.4	0.07/0.04/0.04/Q
	NMeFOSA	ND (RL: 300)/ND (RL: 300)/ND (RL: 300)	—/—/—	ND/ND/ND	—/—/—	ND/ND/ND	—/—/—	ND/ND/ND/ND	—/—/—/Q	< RL (J)/ND/ND/ND	—/—/—/Q
Perfluorooctane sulfonamidoacetic acids	NMeFOSAA	ND (RL: 200)/ND (RL: 200)/ND (RL: 200)	—/—/—	ND/< RL (J)/< RL (J)	—/—/—	ND/ND/ND	—/—/—	ND/ND/ND/ND	—/—/—/Q	ND/ND/ND/ND	—/—/—/Q
	NEtFOSAA	ND (RL: 300)/ND (RL: 300)/ND (RL: 300)	—/—/—	< RL (J)/< RL (J)/< RL (J)	—/—/—	ND/ND/ND	—/—/—	ND/ND/ND/ND	—/—/—/Q	ND/ND/ND/ND	—/—/—/Q
	**Units**	**AOF, EOF: μg F/L, ng F/g**	**mg F/run**	**AOF, EOF: μg F/L, ng F/g**	**mg F/run**	**AOF, EOF: μg F/L, ng F/g**	**mg F/run**	**AOF, EOF: μg F/L, ng F/g**	**mg F/run**	**AOF, EOF: μg F/L, ng F/g**	**mg F/run**
TOF	AOF	220 (RL: 20)/< RL/160 (RL: 20)	63426.66/—/45295.33	NA/NA/NA	NA/—/NA	NA/NA/NA	NA/NA/NA	< RL/< RL/< RL/< RL	—/—/—/—	3.2/2.6/< RL/2.2	29.8/24.11/—/—
	EOF	NA/NA/NA	NA/NA/NA	ND/ND/ND	—/—/—	NA/NA/NA	NA/NA/NA	NA/NA/NA/NA	NA/NA/NA/NA	NA/NA/NA/NA	NA/NA/NA/NA

Abbreviations: *‐: LCS and/or LCSD outside acceptance limits, low biased. Result not reported; *1: LCS and or LCSD relative percent difference outside acceptance limits. Results not reported; AOF: absorbable organic fluorine; CI: chromatographic interference, Result not reported; EOF: extractable organic fluorine; F: fluorine; I: EMPC due to interference. Result not reported;  J: result is less than the reporting limit but greater than or equal to the minimum detection limit. Result not reported; mg/run: milligrams per run; NA: not analyzed; ND: nondetect; ng/g: nanograms per gram; ng/L: nanograms per liter; ng/sample: nanograms per sample; Q: result removed during quality control; R(1/2/3/4): run (1/2/3/4); RL: reporting limit; TOF: total organic fluorine.

#### 3.1.1 Thickened Solids

Thickened solids represent the primary feedstock to the centrifuges and have often been identified as containing PFAS (Oza et al. 2023, 2025). Significant amounts of organic fluorine were found in the liquid fraction of this feedstock (2.86 mol of fluorine/run [mol F/run]), but no reportable targeted PFAS were identified. Reportable PFOS were found in the solid fraction in all three runs, and a reportable amount of PFDA was found during the third run. TOF analysis found no reportable amounts of organic fluorine in the solid fraction. Both the RLs and MDLs for the thickened solids were increased due to sample matrix interference. For EOF, the RL was raised to 0.17 mol F/run and the MDL to 0.06 mol F/run, which represents a 105% increase compared to the centrate samples and a 620% increase compared to the dewatered solids samples. For targeted analysis, the RL was raised from 0.0021 to 0.0044 mol F/run, representing a 91%–900% increase compared to centrate and dewatered solids samples. Results indicate that the bulk of the TOF in the liquid fraction was not comprised of targeted PFAS, and any TOF in the solid fraction, including targeted PFAS analytes, was below the MDL. Given the larger amount of both TOF and specific targeted PFAS analytes in dewatered solids exiting the centrifuges (in Section 3.2.1), it is possible that the raised limits from matrix interferences during the analysis of the solids fraction of these samples caused TOF and targeted PFAS analytes to be underreported.

#### 3.1.2 Combustion Air

In all three runs, only one PFAS was found in reportable amounts: PFHxA (a short‐chain PFAS compound). PFHxA was several orders of magnitude lower than other system inputs and does not represent a substantial contribution to the dryer. It could be a result of PFAS release from the dryer, as it was detected in the dryer exhaust, or could originate from atmospheric deposition (Liu et al. 2019; Wu et al. 2019).

#### 3.1.3 Process Water

Organic fluorine between the RL (10 μg·L^−1^) and MDL (5 μg·L^−1^) was detected in process cooling water fed to the condenser and Venturi scrubber. Six PFAS from the perfluoroalkyl carboxylic acid family and three from the perfluoroalkyl sulfonic acid family were reported above RLs. These included PFBA, PFPeA, PFHxA, PFHpA, PFOA, and PFDA from the carboxylic acid family, and PFBS, PFHxS, and PFOS from the sulfonic acid family. Additionally, one perfluorooctane sulfonamide, FOSA, had a reportable concentration in the first run. TOF RLs were raised for these samples due to matrix interference. On average, 65% of PFAS detected in process water, on a molar basis, were short‐chain compounds.

#### 3.1.4 Potable Water

TOF analysis for potable water for cooling the Venturi scrubber had reportable organic fluorine in the first two runs. FOSA was the only targeted analyte reported in all three runs, accounting for 0.09% of the TOF reported on a molar basis. The TOF analyses may indicate that non‐targeted, non‐identified PFAS or other fluorinated compounds were present in the first two runs.

### 3.2 Intermediate Sampling Points: PFAS and Fluorine‐Based Compounds

Data presented in Table 3 represents PFAS measured in the dewatering and drying systems. This includes the dewatered solids fed to the dryer and the dryer exhaust post‐Venturi scrubber. The reportable results from the analysis are presented in Table 3; all data are presented in Table S11.

**TABLE 3:** Dryer system intermediate samples—reportable results.

		Dewatered sewage sludge		RTO inlet (OTM‐45 front half wash)		RTO inlet (OTM‐45 back half wash)		RTO inlet (OTM‐45 impingers)		RTO inlet (OTM‐45 breakthrough XAD cartridge)	
		Concentration	Emission	Concentration	Emission	Concentration	Emission	Concentration	Emission	Concentration	Emission
Units		ng/g	mg/run	ng/sample	mg/run	ng/sample	mg/run	ng/sample	mg/run	ng/sample	mg/run
PFAS family	Acronym	(R1/R2/R3/R3 duplicate)	(R1/R2/R3/R3 duplicate)	(R1/R2/R3/R4)	(R1/R2/R3/R4)	(R1/R2/R3/R4)	(R1/R2/R3/R4)	(R1/R2/R3/R4)	(R1/R2/R3/R4)	(R1/R2/R3/R4)	(R1/R2/R3/R4)
Cyclic PFAS	PFECHS	NA/NA/NA/NA	NA/NA/NA/NA	ND/ND/ND/ND	—/—/—/—	ND/ND/ND/ND	—/—/—/—	ND/ND/ND/ND	—/—/—/—	ND/ND/ND/ND	—/—/—/—
Ether sulfonic acids	9Cl‐PF3ONS	ND/ND/ND/ND	—/—/—/—	ND/ND/ND/ND	—/—/—/—	ND/ND/ND/ND	—/—/—/—	ND/ND/ND/ND	—/—/—/—	ND/ND/ND/ND	—/—/—/—
	11Cl‐PF3OUdS	ND/ND/ND/ND	—/—/—/—	ND/ND/ND/ND	—/—/—/—	*−/*−/*−/*−	—/—/—/—	*− *1/*− *1/*− *1/*− *1	—/—/—/—	*−/*−/*−/*−	—/—/—/—
Fluorotelomer carboxylic acids	3:3 FTCA	NA/NA/NA/NA	NA/NA/NA/NA	ND/ND/ND/ND	—/—/—/—	ND/ND/ND/ND	—/—/—/—	ND/ND/ND/ND	—/—/—/—	ND/ND/ND/ND	—/—/—/—
	5:3 FTCA	NA/NA/NA/NA	NA/NA/NA/NA	9.34/ND/ND/ND	9.34E−06/—/—/—	*+/*+/*+/*+	Q/Q/—/—	162/221/ND/ND	1.62E−04/2.21E−04/—/—	J *+/J *+/J *+/*+	Q/Q/Q/—
	6:2 FTCA	NA/NA/NA/NA	NA/NA/NA/NA	< RL (J)/ND/ND/ND	—/—/—/—	36.9/24.6/ND/ND	3.69E−05/2.46E−05/—/—	< RL (J)/ND/ND/ND	—/—/—/—	2.94/3.93/6.98/ND	2.94E−06/3.93E−06/6.98E−06/—
	7:3 FTCA	NA/NA/NA/NA	NA/NA/NA/NA	< RL (J)/ND/ND/ND	—/—/—/—	5.08/ND/ND/ND	5.08E−06/—/—/—	51.2/ND/ND/ND	5.12E−05/—/—/—	< RL (J)/ND/ND/ND	—/—/—/—
	8:2 FTCA	NA/NA/NA/NA	NA/NA/NA/NA	ND/ND/ND/ND	—/—/—/—	6.17/ND/ND/ND	6.17E−06/—/—/—	ND/ND/ND/ND	—/—/—/—	ND/ND/ND/ND	—/—/—/—
	10:2 FTCA	NA/NA/NA/NA	NA/NA/NA/NA	ND/ND/ND/ND	—/—/—/—	J I/ND/ND/ND	Q/—/—/—	ND/ND/ND/ND	—/—/—/—	ND/ND/ND/ND	—/—/—/—
Fluorotelomer sulfonic acids	4:2 FTS	ND/ND/ND/ND	—/—/—/—	ND/ND/ND/ND	—/—/—/—	ND/ND/ND/ND	—/—/—/—	ND/ND/ND/ND	—/—/—/—	ND/ND/ND/ND	—/—/—/—
	6:2 FTS	ND/ND/ND/ND	—/—/—/—	ND/ND/ND/ND	—/—/—/—	ND/ND/ND/ND	—/—/—/—	ND/ND/ND/ND	—/—/—/—	ND/ND/ND/ND	—/—/—/—
	8:2 FTS	ND/ND/ND/< RL (J)	—/—/—/—	< RL (J)/ND/ND/ND	—/—/—/—	6.26/< RL (J)/ND/ND	6.26E−06/—/—/—	ND/ND/ND/ND	—/—/—/—	ND/ND/ND/ND	—/—/—/—
	10:2 FTS	< RL (J)/11/< RL (J)/11	—/58.44/—/16.53	< RL (J)/ND/ND/ND	—/—/—/—	2.62/ND/ND/ND	2.62E−06/—/—/—	ND/ND/ND/ND	—/—/—/—	ND/ND/ND/ND	—/—/—/—
Fluorotelomer unsaturated carboxylic acids	6:2 FTUCA	NA/NA/NA/NA	NA/NA/NA/NA	ND/ND/ND/ND	—/—/—/—	21.6/< RL (J)/ND/ND	2.16E−05/—/—/—	ND/ND/ND/ND	—/—/—/—	1.31/1.98/2.65/ND	1.31E−06/1.98E−06/2.65E−06/—
	8:2 FTUCA	NA/NA/NA/NA	NA/NA/NA/NA	ND/ND/ND/ND	—/—/—/—	5.5/ND/ND/ND	5.50E−06/—/—/—	ND/ND/ND/ND	—/—/—/—	ND/ND/ND/ND	—/—/—/—
Perfluoroether and Polyfluoroether carboxylic acids	PFMPA	NA/NA/NA/NA	NA/NA/NA/NA	ND/ND/ND/ND	—/—/—/—	ND/ND/ND/ND	—/—/—/—	ND/ND/ND/ND	—/—/—/—	ND/ND/ND/ND	—/—/—/—
	PFMBA	NA/NA/NA/NA	NA/NA/NA/NA	ND/ND/ND/ND	—/—/—/—	ND/ND/ND/ND	—/—/—/—	ND/ND/ND/ND	—/—/—/—	ND/ND/ND/ND	—/—/—/—
	NFDHA	NA/NA/NA/NA	NA/NA/NA/NA	ND/ND/ND/ND	—/—/—/—	ND/ND/ND/ND	—/—/—/—	ND/ND/ND/ND	—/—/—/—	ND/ND/ND/ND	—/—/—/—
	HFPODA	ND/ND/ND/ND	—/—/—/—	I B/B/B/B	Q/Q/Q/Q	I B/B/B/B	Q/Q/Q/Q	B/B/B/B	Q/Q/Q/Q	ND/21.4/117/33.6	—/2.14E−05/1.17E−04/3.36E−05
	DONA	ND/ND/ND/ND	—/—/—/—	ND/ND/ND/ND	—/—/—/—	ND/ND/ND/ND	—/—/—/—	ND/ND/ND/ND	—/—/—/—	ND/ND/ND/ND	—/—/—/—
Perfluoroalkyl carboxylic acids	PFBA	ND/ND/ND/ND	—/—/—/—	4.05/ND/ND/ND	4.05E−06/—/—/—	36.5/ND/ND/ND	3.65E−05/—/—/—	< RL (J)/ND/ND/ND	—/—/—/—	CI/ND/ND/CI	—/—/—/—
	PFPeA	< RL (J)/ND/ND/ND	—/—/—/—	3.81/< RL (J)/ND/< RL (J)	3.81E−06/—/—/—	30.1/37.6/ND/ND	3.01E−05/3.76E−05/—/—	< RL (J)/< RL (J)/< RL (J)/ND	—/—/—/—	1.64/2.38/2.76/ND	1.64E−06/2.38E−06/2.76E−06/—
	PFHxA	5.6/7.2/5.2/5.8	29.19/38.25/31.78/8.72	7.06/< RL (J)/ND/< RL (J)	7.06E−06/—/—/—	305/366/345/ND	3.05E−04/3.66E−04/3.45E−04/—	< RL (J)/ND/ND/ND	—/—/—/—	11.3/18.3/23.6/ND	1.13E−05/1.83E−05/2.36E−05/—
	PFHpA	ND/ND/ND/ND	—/—/—/—	< RL (J)/ND/ND/ND	—/—/—/—	15.4/ND/ND/ND	1.54E−05/—/—/—	ND/ND/ND/ND	—/—/—/—	ND/ND/ND/ND	—/—/—/—
	PFOA	6.3/4.8/4/5.5	32.84/25.5/24.44/8.26	5.4/ND/ND/ND	5.40E−06/—/—/—	B/B/B/B	Q/Q/—/—	ND/ND/ND/ND	—/—/—/—	1.05/1.57/1.21/1.2	1.05E−06/1.57E−06/1.21E−06/1.20E−06
	PFNA	< RL (J)/< RL (J)/< RL (J)/< RL (J)	—/—/—/—	< RL (J)/ND/ND/ND	—/—/—/—	5.77/ND/ND/ND	5.77E−06/—/—/—	ND/ND/ND/ND	—/—/—/—	ND/ND/ND/ND	—/—/—/—
	PFDA	10/11/10/11	52.12/58.44/61.11/16.53	< RL (J)/ND/ND/ND	—/—/—/—	36.2/31.5/ND/ND	3.62E−05/3.15E−05/—/—	ND/ND/ND/ND	—/—/—/—	ND/ND/ND/ND	—/—/—/—
	PFUnA	< RL (J)/< RL (J)/< RL (J)/< RL (J)	—/—/—/—	ND/ND/ND/ND	—/—/—/—	< RL (J)/ND/ND/ND	—/—/—/—	ND/ND/ND/ND	—/—/—/—	ND/ND/ND/ND	—/—/—/—
	PFDoA	3.7/4.4/4.3/4.1	19.28/23.37/26.28/6.16	< RL (J)/ND/ND/ND	—/—/—/—	8.87/< RL (J)/ND/ND	8.87E−06/—/—/—	ND/ND/ND/ND	—/—/—/—	ND/ND/ND/ND	—/—/—/—
	PFTriA	ND/ND/ND/ND	—/—/—/—	ND/ND/ND/ND	—/—/—/—	*−/*−/*−/*−	—/—/—/—	ND/ND/ND/ND	—/—/—/—	*−/*−/*−/*−	—/—/—/—
	PFTeA	< RL (J)/< RL (J)/< RL (J)/< RL (J)	—/—/—/—	ND/ND/ND/ND	—/—/—/—	ND/ND/ND/ND	—/—/—/—	ND/ND/ND/ND	—/—/—/—	ND/ND/ND/ND	—/—/—/—
	PFHxDA	ND/ND/ND/ND	—/—/—/—	ND/ND/ND/ND	—/—/—/—	ND/ND/ND/ND	—/—/—/—	ND/ND/ND/ND	—/—/—/—	ND/ND/ND/ND	—/—/—/—
	PFODA	ND/ND/ND/ND	—/—/—/—	ND/ND/ND/ND	—/—/—/—	*− *1/*− *1/*− *1/*− *1	—/—/—/—	ND/ND/ND/ND	—/—/—/—	*− *1/*− *1/*− *1/*− *1	—/—/—/—
Perfluoroalkyl sulfonic acids	PFEESA/PES	NA/NA/NA/NA	NA/NA/NA/NA	ND/ND/ND/ND	—/—/—/—	ND/ND/ND/ND	—/—/—/—	ND/ND/ND/ND	—/—/—/—	ND/ND/ND/ND	—/—/—/—
	PFBS	ND/ND/ND/ND	—/—/—/—	ND/ND/ND/ND	—/—/—/—	ND/ND/ND/ND	—/—/—/—	ND/ND/ND/ND	—/—/—/—	ND/ND/ND/ND	—/—/—/—
	PFPeS	ND/ND/ND/ND	—/—/—/—	ND/ND/ND/ND	—/—/—/—	ND/ND/ND/ND	—/—/—/—	ND/ND/ND/ND	—/—/—/—	J I/ND/ND/ND	Q/—/—/—
	PFHxS	ND/ND/ND/ND	—/—/—/—	ND/ND/ND/ND	—/—/—/—	ND/ND/ND/ND	—/—/—/—	ND/ND/ND/ND	—/—/—/—	ND/ND/ND/ND	—/—/—/—
	PFHpS	J I/ND/ND/ND	Q/—/—/—	ND/ND/ND/ND	—/—/—/—	ND/ND/ND/ND	—/—/—/—	ND/ND/ND/ND	—/—/—/—	ND/ND/ND/ND	—/—/—/—
	PFOS	21/25/25/23	109.45/132.81/152.77/34.56	ND/ND/ND/ND	—/—/—/—	I/ND/ND/ND	Q/—/—/—	ND/ND/ND/ND	—/—/—/—	ND/ND/ND/ND	—/—/—/—
	PFNS	ND/ND/ND/ND	—/—/—/—	ND/ND/ND/ND	—/—/—/—	ND/ND/ND/ND	—/—/—/—	ND/ND/ND/ND	—/—/—/—	ND/ND/ND/ND	—/—/—/—
	PFDS	< RL (J)/3.7/3.3/< RL (J)	—/19.66/20.17/—	ND/ND/ND/ND	—/—/—/—	*−/*−/*−/*−	—/—/—/—	ND/ND/ND/ND	—/—/—/—	*−/*−/*−/*−	—/—/—/—
	PFDoS	ND/< RL (J)/< RL (J)/< RL (J)	—/—/—/—	J I/ND/ND/ND	Q/—/—/—	*− *1/*− *1/*− *1/*− *1	—/—/—/—	*−/*−/*−/*−	—/—/—/—	*− *1/*− *1/*− *1/*− *1	—/—/—/—
Perfluorooctane sulfonamide ethanols	NMeFOSE	ND/< RL (J)/< RL (J)/ND	—/—/—/—	NA/NA/NA/NA	NA/NA/NA/NA	NA/NA/NA/NA	NA/NA/NA/NA	NA/NA/NA/NA	NA/NA/NA/NA	NA/NA/NA/NA	NA/NA/NA/NA
	NEtFOSE	ND/ND/ND/ND	—/—/—/—	ND/ND/< RL (J)/ND	—/—/—/—	15.5/ND*/ND/ND	1.55E−05/Q/—/—	ND/ND/ND/ND	—/—/—/—	< RL (J)/ND/ND/ND	—/—/—/—
Perfluorooctane sulfonamides	FOSA	< RL (J)/< RL (J)/< RL (J)/< RL (J)	—/—/—/—	ND/ND/ND/ND	—/—/—/—	< RL (J)/ND/ND/ND	—/—/—/—	ND/ND/ND/ND	—/—/—/—	ND/ND/ND/ND	—/—/—/—
	NMeFOSA	ND/ND/ND/ND	—/—/—/—	ND/ND/ND/ND	—/—/—/—	ND/ND/ND/ND	—/—/—/—	ND/ND/ND/ND	—/—/—/—	ND/ND/ND/ND	—/—/—/—
	NEtFOSA	ND/ND/ND/ND	—/—/—/—	ND/ND/ND/ND	—/—/—/—	ND/ND/ND/ND	—/—/—/—	ND/ND/ND/ND	—/—/—/—	ND/ND/ND/ND	—/—/—/—
Perfluorooctane sulfonamidoacetic acids	NMeFOSAA	12/12/13/12	62.54/63.75/79.44/18.03	< RL (J)/ND/ND/ND	—/—/—/—	ND/ND/ND/ND	—/—/—/—	ND/ND/ND/ND	—/—/—/—	ND/ND/ND/ND	—/—/—/—
	NEtFOSAA	15/16/16/16	78.18/85/97.77/24.04	< RL (J)/ND/ND/ND	—/—/—/—	< RL (J)/ND/ND/ND	—/—/—/—	ND/ND/ND/ND	—/—/—/—	ND/ND/ND/ND	—/—/—/—
	**Units**	**AOF, EOF**: **μg F/L, ng F/g**	**mg F/run**	**AOF, EOF**: **μg F/L, ng F/g**	**mg F/run**	**AOF, EOF**: **μg F/L, ng F/g**	**mg F/run**	**AOF, EOF**: **μg F/L, ng F/g**	**mg F/run**	**AOF, EOF**: **μg F/L, ng F/g**	**mg F/run**
TOF	AOF	NA/NA/NA/NA	NA/NA/NA/NA	NA/NA/NA/NA	NA/NA/NA/NA	NA/NA/NA/NA	NA/NA/NA/NA	NA/NA/NA/NA	NA/NA/NA/NA	NA/NA/NA/NA	NA/NA/NA/NA
	EOF	< RL (J) /< RL (J)/< RL (J)/< RL (J)	—/—/—/—	NA/NA/NA/NA	NA/NA/NA/NA	NA/NA/NA/NA	NA/NA/NA/NA	NA/NA/NA/NA	NA/NA/NA/NA	NA/NA/NA/NA	NA/NA/NA/NA

Abbreviations: *+: LCS and/or LCSD is outside acceptance limits, high biased. Result not reported; *−: LCS and/or LCSD is outside acceptance limits, low biased. Result not reported; *1: LCS/LCSD RPD exceeds control limits. Result not reported; AOF: absorbable organic fluorine; B: compound was found in the blank and sample; CI: the peak identified by the data system exhibited chromatographic interference that could not be resolved. There is reason to suspect there may be a high bias; EOF: extractable organic fluorine, F: fluorine; I: value is estimated maximum possible concentration; J: result is less than the reporting limit but greater than or equal to the minimum detection limit. Result not reported; mg/run: milligrams per run; NA: analyte not analyzed for this sample; ND: nondetect; ND*: isotope dilution analyte did not recover, insufficient sample available to retest. Marked as nondetect; ng/g: nanograms per gram; ng/sample: nanograms per sample; Q: result removed during quality control; R(1/2/3/4): run (1/2/3/4); RL: reporting limit; TOF: total organic fluorine.

#### 3.2.1 Dewatered Solids

Dewatered solids were collected from the dewatering centrifuge outlet before the dryer feed bin. Organic fluorine by TOF was noted between the detection (650 ng·g) and reporting (1400 ng·g) limit, and targeted analyses produced reportable amounts of seven PFAS across three families in all three runs. This included PFHxA, PFOA, PFDA, and PFDoA from the perfluoroalkyl carboxylic acids, PFOS from the perfluoroalkyl sulfonic acids, and NEtFOSAA and NMeFOSAA from the perfluorooctane sulfonamidoacetic acids. Additionally, reportable amounts of 10:2 FTS were found in the second run, and reportable amounts of PFDS were measured in runs two and three, but not the first run. PFHxA was the sole short‐chain compound detected in this sample in reportable amounts.

#### 3.2.2 Dryer Exhaust

Dryer exhaust was collected after the Venturi scrubber and after the internal recycle to the furnace. An accurate summation of targeted PFAS in this sample is difficult to calculate due to laboratory control samples being outside of acceptance limits for several targeted analytes across several sample fractions of the OTM‐45 train. Reportable amounts of targeted PFAS were found throughout the sampling train; PFAS measured in all three runs included 6:2 FTCA, PFPeA, PFHxA, and PFOA. Two analytes, 5:3 FTCA and PFDA, were measured in the first two runs but not the third. Nine analytes were measured in the first run only: 7:3 FTCA, 8:2 FTS, 10:2 FTS, 8:2 FTUCA, PFBA, PFHpA, PFNA, PFDoA, and NEtFOSE. Additionally, HFPO‐DA was measured in all runs and in the field blank in three of the four OTM‐45 fractions. Discounting HFPO‐DA, on average, 62% of the analytes detected in this sample on a molar basis were short‐chain compounds.

### 3.3 Dryer Outputs: PFAS and Fluorine‐Based Compounds

PFAS outputs consisting of dried solids, RTO flue gas, and the process drains from dewatering (referred to as centrate), the condenser, and the Venturi scrubber are presented in Table 4. Centrate drained from the centrifuges and cooling water drained from the condenser and Venturi scrubber are recycled back to the WRRF. The reportable results of these samples are presented in Table 4; all data are presented in Table S12.

**TABLE 4:** Dryer system emissions samples ‐ reportable results.

Emissions samples											

		Centrate (liquid fraction)		Centrate (solid fraction)		Dried solids		Condenser process drain		Scrubber process drain	
Concentration	Emission	Concentration	Emission	Concentration	Emission	Concentration	Emission	Concentration	Emission
Units		ng/L	mg/run	ng/g	mg/run	ng/g	mg/run	ng/L	mg/run	ng/L	mg/run
PFAS family	Acronym	*(R1/R2/R3)*	*(R1/R2/R3)*	*(R1/R2/R3)*	*(R1/R2/R3)*	*(R1/R2/R3)*	*(R1/R2/R3)*	*(R1/R2/R3/R4)*	*(R1/R2/R3/R4)*	*(R1/R2/R3/R4)*	*(R1/R2/R3/R4)*
Fluorotelomer carboxylic acids	5:3 FTCA	NA/NA/NA	NA/NA/NA	NA/NA/NA	NA/NA/NA	NA/NA/NA	NA/NA/NA	NA/NA/NA/NA	NA/NA/NA/NA	NA/NA/NA/NA	NA/NA/NA/NA
Fluorotelomer sulfonic acids	8:2 FTS	ND (RL: 30)/ND (RL: 30)/ND (RL: 30)	—/—/—	ND/ND/ND	—/—/—	< RL (J)/< RL (J)/< RL (J)	—/—/—	ND (RL: 29)/ND/ND/ND	—/—/—/Q	ND/ND/ND/ND	—/—/—/Q
	10:2 FTS	ND (RL: 50)/ND (RL: 50)/ND (RL: 49)	—/—/—	< RL (J)/< RL (J)/< RL (J)	—/—/—	2.4/2.8/2.7	12.17/14.77/16.48	ND (RL: 48)/ND/ND/ND	—/—/—/Q	ND/ND/ND/ND	—/—/—/Q
Perfluoroether and Polyfluoroether carboxylic acids	HFPODA	ND (RL: 30)/ND (RL: 30)/ND (RL: 30)	—/—/—	ND/ND/ND	—/—/—	ND/ND/ND	—/—/—	ND (RL: 29)/ND/ND/ND	—/—/—/Q	ND/ND/ND/ND	—/—/—/Q
	DONA	ND (RL: 20)/ND (RL: 20)/ND (RL: 20)	—/—/—	ND/ND/ND	—/—/—	ND/ND/ND	—/—/—	ND (RL: 19)/ND/ND/ND	—/—/—/Q	ND/ND/ND/ND	—/—/—/Q
Perfluoroalkyl carboxylic acids	PFBA	ND (RL: 50)/ND (RL: 50)/ND (RL: 49)	—/—/—	ND/ND/ND	—/—/—	ND/ND/ND	—/—/—	ND (RL: 48)/8.1/9.1/7.5	—/3.9/4.36/Q	7/7/7.4/7.3	0.07/0.06/0.07/Q
	PFPeA	32 (RL: 20)/32 (RL: 20)/31 (RL: 20)	8.51/8.57/7.98	ND/ND/ND	—/—/—	< RL (J)/< RL (J)/< RL (J)	—/—/—	24 (RL: 19)/21/21/22	11.6/10.11/10.07/Q	19/19/20/22	0.18/0.18/0.18/Q
	PFHxA	61 (RL: 20)/67 (RL: 20)/64 (RL: 20)	16.22/17.94/16.48	< RL (J)/< RL (J)/< RL (J)	—/—/—	0.75/0.86/0.81	3.8/4.54/4.94	29 (RL: 19)/26/29/33	14.02/12.51/13.9/Q	25/25/25/28	0.23/0.23/0.23/Q
	PFHpA	20 (RL: 20)/20 (RL: 20)/20 (RL: 20)	5.32/5.36/5.15	ND/ND/ND	—/—/—	ND/ND/ND	—/—/—	< RL (RL: 19) (J)/8.9/9/9.5	—/4.28/4.31/Q	6.1/7.3/7.8/7.1	0.06/0.07/0.07/Q
	PFOA	71 (RL: 20)/68 (RL: 20)/69 (RL: 20)	18.88/18.21/17.76	< RL (J)/< RL (J)/< RL (J)	—/—/—	1.3/1.3/1.3	6.59/6.86/7.93	< RL (RL: 19) (J)/19/19/23	—/9.15/9.11/Q	15/14/15/14	0.14/0.13/0.14/Q
	PFNA	< RL (RL: 20) (J)/< RL (RL: 20) (J)/< RL (RL: 20) (J)	—/—/—	ND/ND/ND	—/—/—	0.81/0.81/0.81	4.11/4.27/4.94	ND (RL: 19)/2/1.9/2.2	—/0.96/0.91/Q	< RL (J)/< RL (J)/< RL (J)/< RL (J)	—/—/—/—
	PFDA	< RL (RL: 20) (J)/< RL (RL: 20) (J)/< RL (RL: 20) (J)	—/—/—	13/10/14	33.17/21.94/9.72	4.6/4.5/4.3	23.33/23.75/26.24	ND (RL: 19)/2.6/2.4/2.5	—/1.25/1.15/Q	< RL (J)/< RL (J)/1.8/< RL (J)	—/—/0.02/—
	PFUnA	ND (RL: 20)/ND (RL: 20)/ND (RL: 20)	—/—/—	ND/ND/ND	—/—/—	1/1.2/1	5.07/6.33/6.1	ND (RL: 19)/ND/ND/ND	—/—/—/Q	ND/ND/ND/ND	—/—/—/Q
	PFDoA	ND (RL: 20)/ND (RL: 20)/ND (RL: 20)	—/—/—	< RL (J)/< RL (J)/< RL (J)	—/—/—	1.6/1.9/1.8	8.11/10.03/10.98	ND (RL: 19)/ND/ND/ND	—/—/—/Q	< RL (J)/ND/ND/ND	—/—/—/Q
	PFTeA	ND (RL: 20)/ND (RL: 20)/ND (RL: 20)	—/—/—	ND/ND/ND	—/—/—	< RL (J)/< RL (J)/< RL (J)	—/—/—	ND (RL: 19)/ND/ND/ND	—/—/—/Q	ND/ND/ND/ND	—/—/—/Q
	PFHxDA	ND (RL: 30)/ND (RL: 30)/ND (RL: 30)	—/—/—	ND/ND/ND	—/—/—	< RL (J)/< RL (J)/< RL (J)	—/—/—	ND (RL: 29)/ND/ND/ND	—/—/—/Q	ND/ND/ND/ND	—/—/—/Q
Perfluoroalkyl sulfonic acids	PFBS	< RL (RL: 20) (J)/< RL (RL: 20) (J)/< RL (RL: 20) (J)	—/—/—	ND/ND/ND	—/—/—	ND/ND/ND	—/—/—	< RL (RL: 19) (J)/4/3.7/4.7	—/1.93/1.77/Q	5.4/4.4/4.3/4.8	0.05/0.04/0.04/Q
	PFPeS	ND (RL: 20)/ND (RL: 20)/ND (RL: 20)	—/—/—	ND/ND/ND	—/—/—	ND/ND/ND	—/—/—	ND (RL: 19)/< RL (J)/< RL (J)/J I	—/—/—/Q	< RL (J)/ND/< RL (J)/< RL (J)	—/—/—/—
	PFHxS	< RL (RL: 20) (J)/< RL (RL: 20) (J)/< RL (RL: 20) (J)	—/—/—	ND/ND/ND	—/—/—	ND/ND/ND	—/—/—	ND (RL: 19)/3/3/4.1	—/1.44/1.44/Q	2.7/2.3/2.5/2.4	0.03/0.02/0.02/Q
	PFOS	22 (RL: 20)/25 (RL: 20)/22 (RL: 20)	5.85/6.69/5.66	28/22/32	71.44/48.28/22.23	9/10/9.9	45.64/52.77/60.42	ND (RL: 19)/7.2/7.1/7.4	—/3.47/3.4/Q	5.4/4.6/5.1/3.9	0.05/0.04/0.05/Q
	PFDS	ND (RL: 20)/ND (RL: 20)/ND (RL: 20)	—/—/—	ND/< RL (J)/J I	—/—/Q	1.2/1.3/1.1	6.09/6.86/6.71	ND (RL: 19)/ND/ND/ND	—/—/—/Q	ND/ND/ND/ND	—/—/—/Q
	PFDoS	ND (RL: 30)/ND (RL: 30)/ND (RL: 30)	—/—/—	< RL (J)/< RL (J)/< RL (J)	—/—/—	< RL (J)/< RL (J)/< RL (J)	—/—/—	ND (RL: 29)/ND/ND/ND	—/—/—/Q	ND/ND/ND/ND	—/—/—/Q
Perfluorooctane sulfonamide ethanols	NMeFOSE	ND (RL: 30)/ND (RL: 30)/ND (RL: 30)	—/—/—	ND/ND/ND	—/—/—	ND/< RL (J)/ND	—/—/—	ND (RL: 29)/< RL (J)/< RL (J)/ND	—/—/—/Q	4.2/3.9/3.9/ND	0.04/0.04/0.04/Q
	NEtFOSE	ND (RL: 30)/ND (RL: 30)/ND (RL: 30)	—/—/—	ND/ND/ND	—/—/—	ND/< RL (J)/< RL (J)	—/—/—	ND (RL: 29)/< RL (J)/< RL (J)/ND	—/—/—/Q	< RL (J)/< RL (J)/< RL (J)/ND	—/—/—/Q
Perfluorooctane sulfonamides	FOSA	ND (RL: 20)/ND (RL: 20)/ND (RL: 20)	—/—/—	ND/ND/ND	—/—/—	< RL (J)/< RL (J)/< RL (J)	—/—/—	ND (RL: 19)/< RL (J)/< RL (J)/ND	—/—/—/Q	< RL (J)/< RL (J)/< RL (J)/ND	—/—/—/Q
Perfluorooctane sulfonamidoacetic acids	NMeFOSAA	ND (RL: 20)/ND (RL: 20)/ND (RL: 20)	—/—/—	< RL (J)/< RL (J)/< RL (J)	—/—/—	3.2/3.8/4.1	16.23/20.05/25.02	ND (RL: 19)/< RL (J)/< RL (J)/< RL (J)	—/—/—/—	< RL (J)/ND/< RL (J)/ND	—/—/—/Q
	NEtFOSAA	ND (RL: 30)/ND (RL: 30)/ND (RL: 30)	—/—/—	< RL (J)/< RL (J)/< RL (J)	—/—/—	5.6/6.4/5.9	28.4/33.77/36.01	ND (RL: 29)/< RL (J)/< RL (J)/ND	—/—/—/Q	< RL (J)/< RL (J)/< RL (J)/ND	—/—/—/Q
	Units	**AOF, EOF: μg F/L, ng F/g**	**mg F/run**	**AOF, EOF: μg F/L, ng F/g**	**mg F/run**	**AOF, EOF: μg F/L, ng F/g**	**mg F/run**	**AOF, EOF: μg F/L, ng F/g**	**mg F/run**	**AOF, EOF: μg F/L, ng F/g**	**mg F/run**
TOF	AOF	20 (RL: 20)/21 (RL: 20)/< RL	5318.69/5623.32/—	NA/NA/NA	—/—/—	NA/NA/NA	NA/NA/NA	< RL/ND (RL: 10)/ND (RL: 10)/ND (RL: 10)	—/—/—/—	< RL/ND (RL: 10)/< RL/< RL	—/—/—/—
	EOF	NA/NA/NA	NA/NA/NA	ND/ND/ND	—/—/—	320/320/310	1622.78/1688.56/1891.82	NA/NA/NA/NA	NA/NA/NA/NA	NA/NA/NA/NA	NA/NA/NA/NA

Emissions samples									
		RTO outlet (OTM‐45 front half wash)		RTO outlet (OTM‐45 back half wash)		RTO outlet (OTM‐45 impingers)		RTO outlet (OTM‐45 breakthrough XAD cartridge)	
		Concentration	Emission	Concentration	Emission	Concentration	Emission	Concentration	Emission
Units		ng/sample	mg/run	ng/sample	mg/run	ng/sample	mg/run	ng/sample	mg/run
PFAS family	Acronym	*(R1/R2/R3)*	*(R1/R2/R3)*	*(R1/R2/R3)*	*(R1/R2/R3)*	*(R1/R2/R3)*	*(R1/R2/R3)*	*(R1/R2/R3)*	*(R1/R2/R3)*
Fluorotelomer carboxylic acids	5:3 FTCA	ND/ND/ND	—/—/—	*+/*+/J *+	—/—/Q	ND/ND/ND	—/—/—	*+/*+/*+	—/—/—
Fluorotelomer sulfonic acids	8:2 FTS	ND/< RL (J)/< RL (J)	—/—/—	ND/ND/ND	—/—/—	ND/ND/ND	—/—/—	ND/ND/ND	—/—/—
	10:2 FTS	ND/ND/ND	—/—/—	ND/ND/ND	—/—/—	ND/ND/ND	—/—/—	ND/ND/ND	—/—/—
Perfluoroether and Polyfluoroether carboxylic acids	HFPODA	B/B/B	Q/Q/Q	B/B/B	Q/Q/Q	B/B/B	Q/Q/Q	43.8/ND/42.1	4.38E−05/—/4.21E−05
	DONA	ND/ND/ND	—/—/—	ND/ND/ND	—/—/—	ND/ND/ND	—/—/—	ND/ND/ND	—/—/—
Perfluoroalkyl carboxylic acids	PFBA	ND/ND/ND	—/—/—	ND/ND/ND	—/—/—	ND/1.63/ND	—/1.63E−06/—	CI/CI/CI	—/—/—
	PFPeA	ND/< RL (J)/ND	—/—/—	ND/ND/ND	—/—/—	ND/0.739/ND	—/7.39E−07/—	ND/ND/ND	—/—/—
	PFHxA	ND/< RL (J)/ND	—/—/—	ND/ND/ND	—/—/—	ND/ND/ND	—/—/—	ND/< RL (J)/ND	—/—/—
	PFHpA	ND/ND/ND	—/—/—	ND/ND/ND	—/—/—	ND/< RL (J)/ND	—/—/—	ND/ND/ND	—/—/—
	PFOA	ND/ND/ND	—/—/—	ND/ND/< RL (J)	—/—/—	ND/1.05/ND	—/1.05E−06/—	< RL (J)/ND/1.41	—/—/1.41E−06
	PFNA	ND/< RL (J)/ND	—/—/—	ND/ND/ND	—/—/—	ND/< RL (J)/ND	—/—/—	ND/ND/ND	—/—/—
	PFDA	ND/ND/ND	—/—/—	ND/ND/ND	—/—/—	ND/ND/ND	—/—/—	ND/ND/ND	—/—/—
	PFUnA	ND/ND/ND	—/—/—	J I/ND/ND	Q/—/—	ND/ND/ND	—/—/—	ND/ND/ND	—/—/—
	PFDoA	ND/ND/ND	—/—/—	J I/ND/J I	Q/—/Q	ND/ND/ND	—/—/—	ND/ND/ND	—/—/—
	PFTeA	ND/ND/ND	—/—/—	ND/ND/ND	—/—/—	ND/ND/ND	—/—/—	ND/ND/ND	—/—/—
	PFHxDA	ND/ND/ND	—/—/—	ND/ND/ND	—/—/—	ND/ND/ND	—/—/—	ND/ND/ND	—/—/—
Perfluoroalkyl sulfonic acids	PFBS	ND/ND/ND	—/—/—	ND/ND/ND	—/—/—	ND/ND/ND	—/—/—	ND/ND/ND	—/—/—
	PFPeS	ND/ND/ND	—/—/—	ND/ND/ND	—/—/—	ND/ND/ND	—/—/—	ND/ND/ND	—/—/—
	PFHxS	ND/ND/ND	—/—/—	ND/ND/ND	—/—/—	ND/ND/ND	—/—/—	ND/ND/ND	—/—/—
	PFOS	ND/ND/ND	—/—/—	ND/ND/ND	—/—/—	ND/J I/ND	—/Q/—	ND/ND/ND	—/—/—
	PFDS	ND/ND/ND	—/—/—	ND/ND/ND	—/—/—	ND/ND/ND	—/—/—	ND/ND/ND	—/—/—
	PFDoS	ND/ND/ND	—/—/—	*− *1/*− *1/*− *1	—/—/—	*−/*−/*−	—/—/—	*− *1/*− *1/*− *1	—/—/—
Perfluorooctane sulfonamide ethanols	NMeFOSE	NA/NA/NA	NA/NA/NA	NA/NA/NA	NA/NA/NA	NA/NA/NA	NA/NA/NA	NA/NA/NA	NA/NA/NA
	NEtFOSE	ND/< RL (J)/< RL (J)	—/—/—	ND/ND/ND	—/—/—	ND/ND/ND	—/—/—	ND/ND/ND	—/—/—
Perfluorooctane sulfonamides	FOSA	ND/ND/ND	—/—/—	ND/ND/ND	—/—/—	ND/ND/ND	—/—/—	ND/ND/ND	—/—/—
Perfluorooctane sulfonamidoacetic acids	NMeFOSAA	ND/ND/ND	—/—/—	ND/ND/ND	—/—/—	ND/ND/ND	—/—/—	ND/ND/ND	—/—/—
	NEtFOSAA	ND/ND/ND	—/—/—	ND/ND/ND	—/—/—	ND/ND/ND	—/—/—	ND/ND/ND	—/—/—
	Units	**AOF, EOF: μg F/L, ng F/g**	**mg F/run**	**AOF, EOF: μg F/L, ng F/g**	**mg F/run**	**AOF, EOF: μg F/L, ng F/g**	**mg F/run**	**AOF, EOF: μg F/L, ng F/g**	**mg F/run**
TOF	AOF	NA/NA/NA	NA/NA/NA	NA/NA/NA	NA/NA/NA	NA/NA/NA	NA/NA/NA	NA/NA/NA	NA/NA/NA
	EOF	NA/NA/NA	NA/NA/NA	NA/NA/NA	NA/NA/NA	NA/NA/NA	NA/NA/NA	NA/NA/NA	NA/NA/NA

Abbreviations: *+: LCS and/or LCSD is outside acceptance limits, high biased. Result not reported; *−: LCS and/or LCSD is outside acceptance limits, low biased. Result not reported; *1: LCS/LCSD RPD exceeds control limits. Result not reported; AOF: absorbable organic fluorine; B: compound was found in the blank and sample; CI: the peak identified by the data system exhibited chromatographic interference that could not be resolved. There is reason to suspect there may be a high biased; EOF: extractable organic fluorine; F: fluorine; I: value is estimated maximum possible concentration; J: result is less than the reporting limit but greater than or equal to the minimum detection limit. Result not reported; mg/run: milligrams per run; NA: analyte not analyzed for this sample; ND: nondetect; ND*: isotope dilution analyte did not recover, insufficient sample available to retest. Marked as nondetect; ng/g: nanograms per gram; ng/sample: nanograms per sample; Q: result removed during quality control; R(1/2/3/4): run (1/2/3/4); RL: reporting limit; TOF: total organic fluorine.

#### 3.3.1 Centrate

Concentrations (0.29 mol F/run) of organic fluorine were measured in the liquid fraction, but organic fluorine in the solid fraction was not detected. Targeted PFAS analytes found in the liquid fraction included reportable amounts of PFPeA, PFHxA, PFHpA, PFOA, and PFOS, averaging 0.0019 mol F/run, and representing 0.67% of the total organic fluorine measured in the liquid fraction of these samples. Targeted PFAS in the solid fraction included reportable amounts of PFDA and PFOS, averaging 0.0024 mol F/run. On average, only 20% of the analytes detected, on a molar basis, were short‐chain compounds.

#### 3.3.2 Dried Solids

Reportable amounts of organic fluorine were measured in dried solids in all runs. While a direct comparison to dewatered solids entering the dryer is impossible due to non‐reportable TOF results in those samples, TOF levels in dried solids (0.091 mol F/run) were reduced by several orders of magnitude when compared with the levels observed in the thickened solids (2.86 mol F/run). Targeted PFAS were reported for 10:2 FTS, PFHxA, PFOA, PFNA, PFDA, PFUnA, PFDoA, PFOS, PFDS, NEtFOSAA, and NMeFOSAA in all runs, accounting for 5.45% of the TOF, reported on a molar basis. PFHxA was the sole short‐chain compound detected in this sample but represented 25% of the average PFAS compounds detected on a molar basis.

#### 3.3.3 Condenser and Venturi Scrubber Drains

TOF analysis did not produce reportable amounts of organic fluorine in the condenser and Venturi scrubber drain samples. Targeted PFAS analysis revealed several analytes present in reportable amounts. Concentrations of seven PFAS from the perfluoroalkyl carboxylic acid family and two from the perfluoroalkyl sulfonic acid family were above RLs in all runs. These included PFBA, PFPeA, PFHxA, PFHpA, PFOA, PFUnA, and PFDoA from the carboxylic acid family, and PFOS and PFDS from the sulfonic acid family. Additionally, one perfluorooctane sulfonamide ethanol, NMeFOSE, was found in all runs. PFNA was detected only in the second run, and PFDA was found in the second and third runs.

Although a mass balance around the emissions scrubbing system could not be closed, the difference in measured targeted PFAS in the cooling water supplied to the system (on a molar basis) was not statistically significant compared to the drains. This was calculated using a Microsoft Excel paired t‐test (alpha = 0.05, two‐tailed), through which the average sum molar mass of PFAS in the process and potable water feed samples were compared to the average sum molar mass of PFAS in the condenser and Venturi scrubber drains. The dominant PFAS family in both samples was the perfluoroalkyl carboxylic acid group. On a molar basis, 72% of the analytes observed in the condenser drains and 66% in the Venturi scrubber drains were short‐chain compounds.

#### 3.3.4 Flue Gas at RTO Outlet

Samples were collected from the RTO flue gas outlet. Calculating an accurate summation of targeted PFAS analytes in this sample was challenging due to contamination of the OTM‐45 train. However, reportable amounts of two targeted PFAS analytes—PFBA (5.33 × 10^−11^ mol F/run) and PFOA (4.46 × 10^−11^ mol F/run)—were found in the second and third runs in substantially lower concentrations compared to the RTO inlet. HFPO‐DA was detected in all runs, but was also present in the field blank for three out of the four OTM‐45 fractions. Discounting HFPO‐DA, on average, 63% of the analytes detected in this sample on a molar basis were short‐chain compounds.

### 3.4 Quality Control Samples

Section 9 of Method OTM‐45 (USEPA 2021) specifies requirements for method performance. QC samples include a field blank train (FBT); a proof blank train (PBT); an XAD cartridge media check; a filter media check; and XAD, filter, methanol recovery reagent, and deionized water reagent blanks (RB). The OTM‐45 QC results for this study are presented in Table S13. The FBT and the PBT consist of fully assembled trains of the front half, back half, impingers, and the breakthrough XAD‐2 cartridge in series, in that order (Figure S2). The QA objective for the data derived from these blanks is to evaluate the effectiveness of the method cleaning procedures in the laboratory and between runs. The FBT uses glassware previously used at the current site from a completed run. The PBT is set up using glassware that has been prepared for this test but before the glassware has been used for sampling.

Assessment of the OTM‐45 QC samples indicated PFAS measured were below RLs for the media (filter or resin), RB solvents, and the breakthrough XAD. However, the analysis found HPFO‐DA in the first three fractions of the FBT, that is, the front half, back half, and impingers, at levels of 276, 243, and 0.826 ng/sample, respectively, but not detected for the breakthrough XAD fraction.

## 4 Discussion

The primary operating conditions that govern thermal processes are commonly referred to as “the 3Ts: time, temperature, and turbulence.” The duration (time) that material remains in the reactor, temperature of the reactor, and turbulence (or mixing) are all important to ensure that the material is fully and evenly exposed to the high temperatures needed for decomposition.

### 4.1 Residence Time

Biosolids rotary drum dryers require both a relatively dry feedstock to prevent material from adhering to the drum surfaces, as well as a considerable amount of process gas to convey heat. Biosolids drum dryers recycle both dried solids and cooled dryer exhaust to achieve the target feedstock moisture content and process gas mass flow, respectively. The dried solids recycle stream extends the residence time of solids‐laden constituents. Similarly, the dryer exhaust recycle extends the residence time of gas‐phase constituents and introduces these compounds to the dryer furnace.

### 4.2 Temperature and Turbulence

Rotary drum dryers distribute heat throughout the drum at a temperature gradient, with the highest temperature at the inlet. During tests, heat from the furnace ranged from 342°C to 451°C, and the outlet temperature ranged between 88°C and 93°C. Variability in inlet temperatures resulted from a range of evaporative loading applied throughout the test run, with a higher inlet temperature corresponding to a higher evaporative loading. Turbulence is achieved by rotating the drum to mix hot process gas with cascading solids. Heat transfer is controlled by maintaining a target particle size and density of blended feed. The process is operated to limit the maximum product temperature to 77°C at the dryer outlet.

### 4.3 PFAS Data Comparison

**FIGURE 2:**
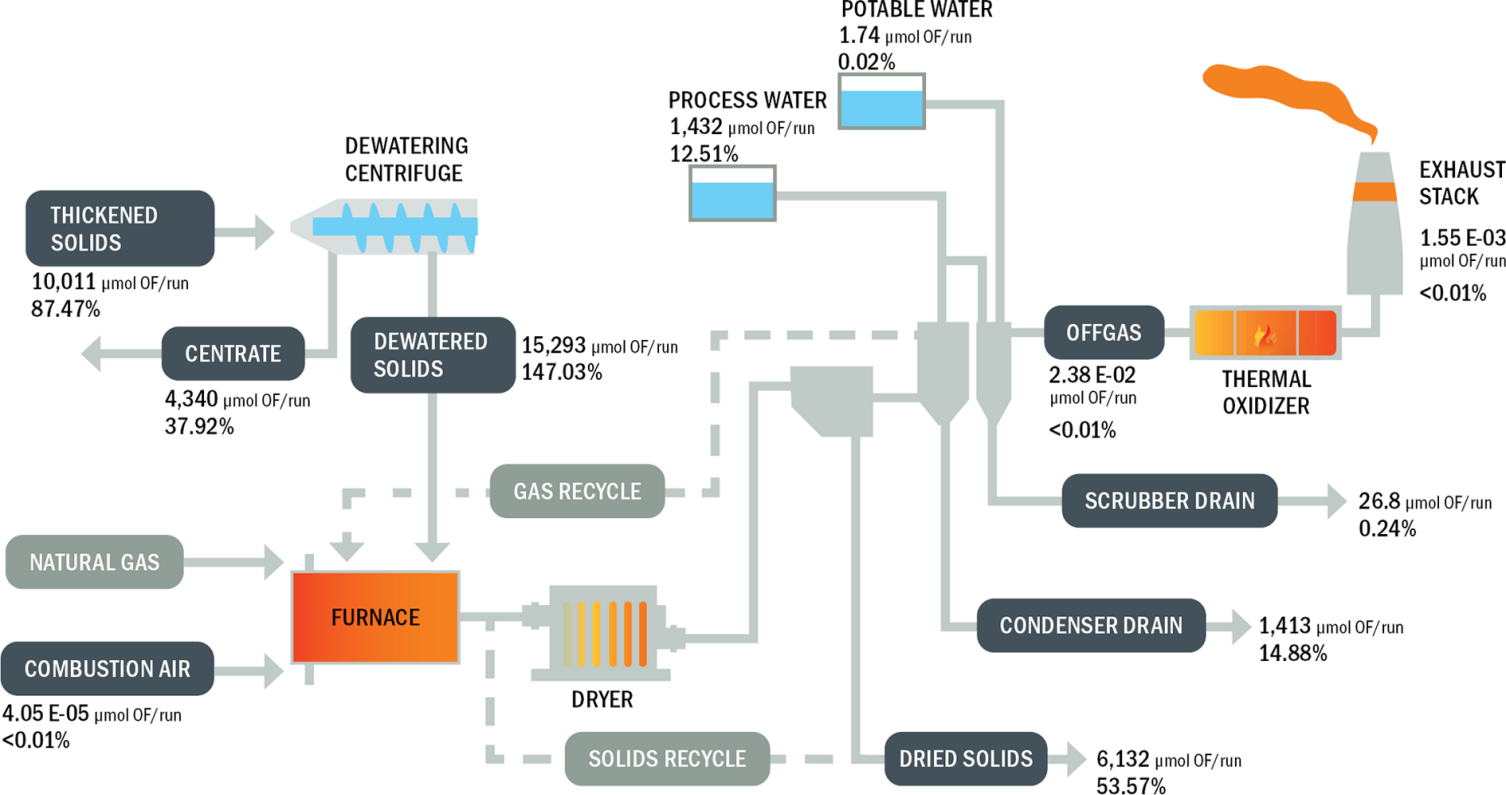
Summary of total average targeted PFAS across three sampling runs.

**FIGURE 3:**
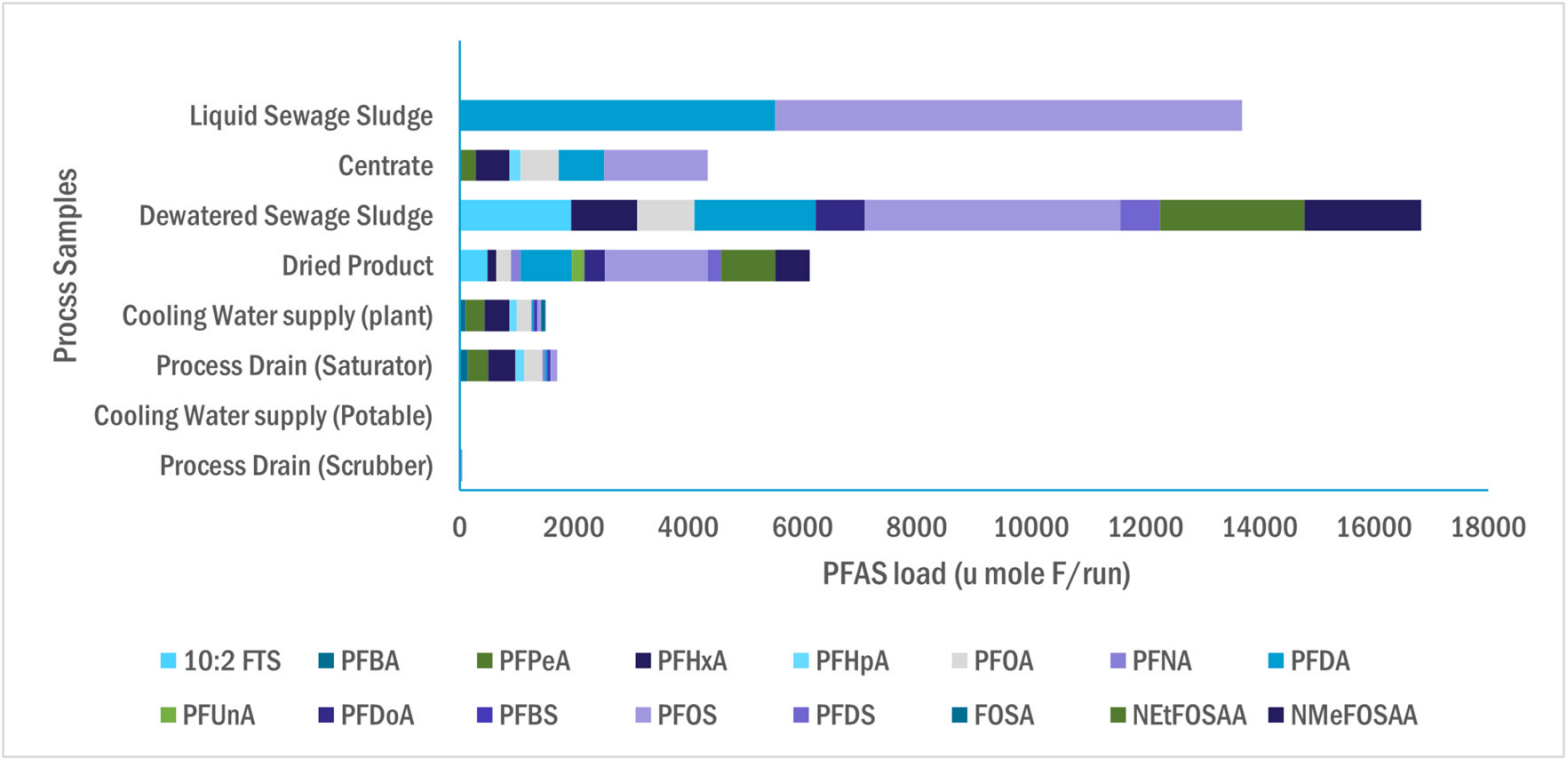
Comparison of PFAS mass reported within process samples.

**FIGURE 4:**
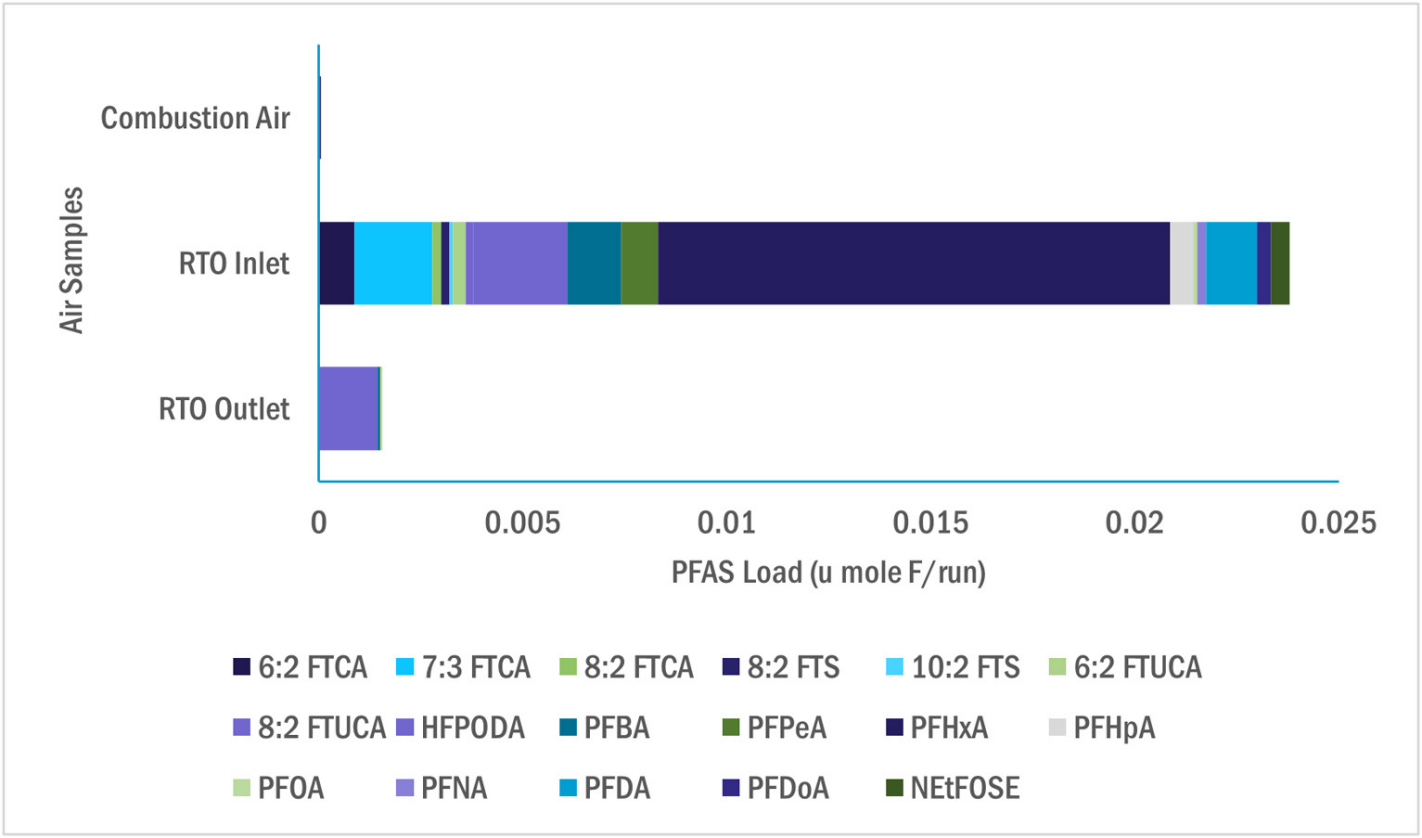
Comparison of PFAS mass reported within air samples.

Figure 2 provides a summary of total targeted PFAS detected at each sample point, represented in molar mass as moles of organic fluorine, and represents the closest approximation toward a targeted PFAS mass balance. Values in Figure 2 were calculated by averaging the summed targeted PFAS molar mass across the three runs. Relative concentrations of individual PFAS are provided for solid and liquid samples on Figure 3 and gas phase samples on Figure 4.

Key findings from the comparative overview are: (a) 60% of the PFAS fed to the dryer in the dewatered solids were absent from dried solids, (b) PFAS were detected in the dryer exhaust at six orders of magnitude less than dewatered solids, (c) PFAS did not appreciably change between the scrubber cooling water supply and drains, and (d) PFAS exhaust from the RTO is called into question given the relatively high contribution of HFPO‐DA. The HFPO‐DA reported was detected in the final fraction of the OTM‐45 sampling train, which was the one fraction out of four not contaminated in the FBT. Consequently, there is no formal justification to screen out its measurement. However, the presence of HFPO‐DA in the other FBT fractions, and the HFPO‐DA propensity to degrade at temperatures substantially lower than those occurring in an RTO, cast doubt on its presence (Blotevogel et al. 2023). When HFPO‐DA data are not considered in RTO exhaust sampling fractions, the RTO achieves a DRE of 99.3%, which may be a more accurate representation of its performance.

### 4.4 Fate of PFAS Through Dewatering

Thickened solids fed to the centrifuge contained few reportable targeted PFAS in either the solid or liquid fraction, and AOF was only found in the liquid fraction. These results are a factor of sample matrix interferences detected by Eurofins that resulted in elevated RLs. The liquid phase of the dewatered solids (mechanically separated) contained reportable PFAS (primarily perfluoroalkyl carboxylic and perfluorooctane sulfonamidoacetic acids), which indicated some PFAS were retained in the liquid phase through dewatering. PFAS were detected in the liquid fraction of the centrate but not in the liquid fraction of thickened solids. The centrifuge operation is analogous to the physical separation of thickened solids into its analyzed solid and liquid fractions, so there should be little difference between PFAS distribution in the thickened solids liquid/solid matrices and the dewatered solids and centrate. Differing RLs (noted previously) for each of these samples may explain the discrepancy; however, it is also possible that using ionic polymers in the dewatering process affected the concentration and partitioning of PFAS into dewatered solids. Hubert et al. (2024) successfully employed cationic and anionic polymers in soil‐washing studies, where the goal was transferring PFAS from contaminated soil into a liquid solution for further PFAS treatment. Various polymers were used in spiked jar tests of soil and Milli‐Q water to test the effectiveness of PFAS removal from a highly turbid organic matrix, which resulted in PFAS removal efficiencies of between 20% and 80% for cationic and anionic polymers (Hubert et al. 2024). While the concentration of organic matter and matrix complexity is presumably higher in biosolids, polymer addition, coupled with the RL variances across samples, may partially explain the variations in PFAS partitioning and increasing levels across the centrifuge.

### 4.5 Fate of PFAS Through Drying

Data shown in Table 3 show targeted PFAS measured in the dryer feed were also detected in the RTO inlet, which suggests some PFAS volatilization, though some discrepancies in the PFAS profile were observed. Differences in the dryer feed rate were assessed over different test runs and showed that the solids loading rate for run 3 was approximately 13% lower than runs 1 and 2, resulting in a lower operating temperature and potentially explaining the lack of detectable PFAS compared to the first two runs (i.e., may have contributed to less volatilization).

Results in Figure 2 demonstrate that PFAS in dried solids accounted for 40% of the total molar mass of targeted PFAS in the dewatered solids feed, while PFAS in the exhaust made up less than 0.002%. This indicates a significant gap in the PFAS mass balance. TOF was not found in reportable ranges in all dewatered solids samples, but a 96% reduction of organic fluorine was observed when comparing the thickened solids and dried solids samples. A possible reason for the lack of PFAS detection in dryer outputs is that PFAS may be transformed within the dryer to end products that were not detected by the analytical techniques used in this study. McNamara et al. (2025) observed PFAS profile shifts through bench‐scale biosolids drying experiments. Findings by Lazcano et al. 2019 and Lakshminarasimman et al. (2021) indicated targeted PFAS concentrations increased though drying. Both referenced studies lend support to the concept of PFAS transformation. Another potential cause for PFAS transformation could be thermal degradation of PFAS volatilized into the dryer exhaust stream which is recycled to the dryer furnace (Shields et al. 2023). PFAS transformation through thermal processes generally results in transformation to smaller, more‐thermally stable compounds that become less polar and more volatile (Blake and Tomlinson 1971; Kissa 2001; Krusic et al. 2005; Krusic and Roe 2004; Winchell et al. 2024). Consequently, further work is recommended to elucidate PFAS transformation products using OTM‐50 in the gas phase. Additionally, further studies should investigate the impact of thermal processing parameters (“3 Ts”) on potential PFAS volatilization as a means of resolving conflicting results between this study and previous findings (Lazcano et al. 2019; Lakshminarasimman et al. 2021).

### 4.6 PFAS Fate Through the Exhaust Scrubbing System

PFAS in the cooling water supply and drains had no statistically significant differences on a mol F/run basis (paired *t*‐test, alpha = 0.05, two tail). Additionally, no reportable increases in AOF or EOF were noted from the supply to drain water samples. Moreover, AOF was not detected in the Venturi scrubber system drain. Results indicate that these scrubbing systems do not act as a sink in capturing gas‐phase PFAS, or release PFAS from the liquid supply streams into the gas phase. This is consistent with Winchell et al. (2024), who found similar results through sewage sludge incinerator exhaust scrubbing systems.

### 4.7 PFAS Fate Through the RTO

The RTO inlet and outlet analytical results were complicated by the detection of HFPO‐DA in three of four FBT fractions and PFOA in a single FBT fraction. This could be due to contamination during bottleware cleaning procedures, use of glassware from a completed sampling run for field blanks, or analytical interferences during sample analyses. HFPO‐DA was not detected in the dewatered solids samples (Table 3). Consequently, the origin of HFPO‐DA in this study is undetermined. Potential reasons for its presence include: (1) HFPO‐DA in the solids feed was below detection limit values, (2) HFPO‐DA exists in a different chemical form in feed solids not measured by the targeted analyses in this study, (3) a reaction that generated HFPO‐DA during processing, and (4) an unknown source of laboratory contamination.

Nevertheless, PFAS reduction through the RTO on a mol F/run basis was statistically significant. This was calculated using Microsoft Excel paired t‐test function (alpha = 0.05, two tail) that compared the average sum molar mass of PFAS in the RTO inlet to the RTO outlet samples. Short‐chain PFAS represented 63% by molar mass of the reportable PFAS in the RTO outlet, and long‐chain PFAS present in the inlet (PFDA, PFNA, and PFDoA) were non‐detect in the outlet. Although a complete mass balance could not be closed, these results indicate thermal degradation, not merely transformation of PFAS. Future work should evaluate stack emissions for PFAS transformation using OTM‐50, as well as inorganic fluoride constituents (i.e., hydrogen fluoride and fluoride salts) to determine to what extent PFAS is degraded or mineralized. HFPO‐DA has been identified as a quality control issue in similar PFAS stationary emissions studies and requires further research to better understand the cause of contamination and verify its presence in OTM‐45 samples (Seay et al. 2023; Winchell et al. 2024).

## 5 Conclusions and Further Research

To the authors' knowledge, this is the first study evaluating the fate of PFAS through a biosolids rotary drum dryer considering gas‐phase emissions. Despite limitations in analytical techniques and contamination of the gas‐phase sampling train that prevented closure of a PFAS mass balance, several key findings were made. First, when targeted PFAS values for the dryer inputs and outputs are considered, it is likely that a considerable amount of PFAS is transferring from dewatered solids to the gas phase in the dryer (60% of targeted analytes). Second, only a small fraction of targeted PFAS was detected in the gas phase (2.37 × 10^−4^% of targeted analytes found in the thickened solids samples on a molar basis), which suggests PFAS is transforming within the dryer or within the exhaust recycle loop through the furnace. Finally, the RTO demonstrates high levels (> 90%) of PFAS destruction or removal, but the actual DRE value is called into question due to HFPO‐DA complications. If the HFPO‐DA values are disregarded, the RTO achieves a DRE of 99.3% for all PFAS on a sum molar mass basis.

Further work is recommended to better understand the drivers behind PFAS phase transfer within the drying system. This includes identifying the extent to which PFAS may be undergoing transformation within solids or being volatilized to the gas stream. Additionally, it is important to determine if PFAS are being transformed within the recycle through the dryer furnace. OTM‐50, in addition to OTM‐45 gas‐phase sampling, will aid in this analysis. Finally, a greater understanding of the cause for HFPO‐DA contamination in this study, as well as similar PFAS stack sampling studies, will be required to advance the state of the science and verify PFAS destruction through high‐temperature processes such as the RTO.

## Author Contributions

**John J. Ross:** conceptualization, investigation, funding acquisition, methodology, writing – original draft, project administration, formal analysis. **Alex Seidel:** formal analysis, data curation, writing – original draft, writing – review and editing. **Embrey Bronstad:** writing – review and editing. **Farokh Kakar:** formal analysis, data curation. **Mary Lou Romero:** writing – review and editing. **Martha J. M. Wells:** conceptualization, investigation, funding acquisition, methodology, validation, visualization, formal analysis, data curation, supervision. **Lloyd J. Winchell:** conceptualization, investigation, methodology, validation, visualization, writing – review and editing, supervision, resources. **Katherine Y. Bell:** conceptualization, investigation, funding acquisition, writing – review and editing, methodology, validation, visualization, formal analysis, supervision, data curation. **Don Song:** conceptualization, investigation, funding acquisition, methodology, project administration, formal analysis.

## Conflicts of Interest

The authors declare no conflicts of interest.

## Supporting information


**Table S1:** Test site operating parameters.
**Figure S2:** Method OTM‐45 sampling train analytical fractions.
**Table S2:** Source air and combustion air sampling methodology.
**Figure S3:** Combustion air sampling scheme.
**Table S3:** Targeted polar PFAS analytes.
**Table S4:** Sample list.
**Table S5:** Judgment criteria on data usability.
**Table S6:** Solid samples flowrates.
**Table S7:** Liquid samples flowrates.
**Table S8:** Gas phase operating conditions.
**Table S9:** Dryer, scrubber, and RTO operating conditions.
**Table S10:** Dryer system input samples—full results.
**Table S11:** Dryer system intermediate samples—full results.
**Table S12:** Dryer system output samples—full results.
**Table S13:** Dryer input flowrates.
**Table S14:** Dryer system intermediate flowrates.
**Table S15:** Dryer system emissions flowrates.
**Table S16:** Summation of targeted analytes molar flows.
**Table S17:** OTM‐45 QC results.
**Table S18:** Combustion air reporting limits.
**Table S19:** RTO inlet reporting limits.
**Table S20:** RTO outlet reporting limits.
**Table S21:** Synagro inputs reporting limits.
**Table S22:** Synagro intermediate reporting limits.
**Table S23:** Synagro emissions reporting limits.

**Figure S1:** Test facility process flow diagram and sample points.

## Data Availability

The data that support the findings of this study are available from the corresponding author upon reasonable request.
