# Supplementary material for: Fate of PFAS Through a Biosolids Drum Dryer With Regenerative Thermal Oxidizer Emissions Control
Source: Water Environ Res. 2025 Oct 20;97(10):e70149. doi: 10.1002/wer.70149 (PMC12536579; doi:10.1002/wer.70149)
Supplement: Supplementary file 1 — Table S1: Test site operating parameters. Figure S2: Method OTM‐45 sampling train analytical fractions. Table S2: Source air and combustion air sampling methodology. Figure S3: Combustion air sampling scheme. Table S3: Targeted polar PFAS analytes. Table S4: Sample list. Table S5: Judgment criteria on data usability. Table S6: Solid samples flowrates. Table S7: Liquid samples flowrates. Table S8: Gas phase operating conditions. Table S9: Dryer, scrubber, and RTO operating conditions. Table S10: Dryer system input samples—full results. Table S11: Dryer system intermediate samples—full results. Table S12: Dryer system output samples—full results. Table S13: Dryer input flowrates. Table S14: Dryer system intermediate flowrates. Table S15: Dryer system emissions flowrates. Table S16: Summation of targeted analytes molar flows. Table S17: OTM‐45 QC results. Table S18: Combustion air reporting limits. Table S19: RTO inlet reporting limits. Table S20: RTO outlet reporting limits. Table S21: Synagro inputs reporting limits. Table S22: Synagro intermediate reporting limits. Table S23: Synagro emissions reporting limits. [file WER-97-e70149-s002.docx]

# **SUPPLEMENTARY INFORMATION**

# Fate of PFAS Through a Biosolids Drum Dryer with Regenerative Thermal Oxidizer Emissions Control

John J. Ross^1*^, Alex Seidel^1^, Embrey Bronstad^1^, Farokh Kakar^1^, Mary Lou Romero^1^, Martha J. M. Wells^2^, Lloyd J. Winchell^1^,Katherine Y. Bell ^1^, Don Song^3^

^1^ Brown and Caldwell, Walnut Creek, California, USA ^2^ EnviroChem Services, Cookeville, Tennessee, US ^3^ Synagro Technologies, Baltimore, Maryland, US

*Corresponding Author

John J. Ross,

Brown and Caldwell

201 North Civic Drive

Walnut Creek, CA

Phone: 978.983.2030

E-mail: [jross@brwncald.com](mailto:jross@brwncald.com)

Table of Contents

**[SUPPLEMENTARY INFORMATION](#_Toc195197644)** [1](#_Toc195197644)

[Fate of PFAS Through a Biosolids Drum Dryer with Regenerative Thermal Oxidizer Emissions Control 1](#_Toc195197645)

[S1. Sample Site Details 3](#_Toc195197647)

[S2. Sampling and Analytical Details 4](#_Toc195197648)

[S2.1 Source Air (Stack) 4](#_Toc195197649)

[USEPA Reference Test Methods 1 and 2 — Volumetric Flow Rate 5](#_Toc195197650)

[USEPA Reference Test Method 3/3A — Oxygen/Carbon Dioxide 5](#_Toc195197651)

[USEPA Reference Test Method 4 — Moisture Content 6](#_Toc195197652)

[USEPA Other Test Method 45 (OTM-45) — PFAS 6](#_Toc195197653)

[S2.2 Combustion Air 7](#_Toc195197654)

[S2.3 Targeted Polar Analytes 7](#_Toc195197655)

[S2.4 TOF 9](#_Toc195197656)

[S2.5 Solids Characteristics 10](#_Toc195197657)

[S2.6 Sampling Event Details 10](#_Toc195197658)

[S2.7 Data Quality Standards 11](#_Toc195197659)

[S3. Operating Data During Sampling Event 13](#_Toc195197660)

[S4. Results 15](#_Toc195197661)

[Flowrates Normalized to Plant Flow 32](#_Toc195197662)

[QC Results 40](#_Toc195197663)

[Reporting Limits 43](#_Toc195197664)

[References 56](#_Toc195197665)

# S1. Sample Site Details

The facility selected for this study utilizes centrifuge dewatering and a conventional rotary drum dryer equipped with a condenser, Venturi scrubber, and regenerative thermal oxidizer (RTO). Figure S-1 shows a schematic of the process, along with sample points.


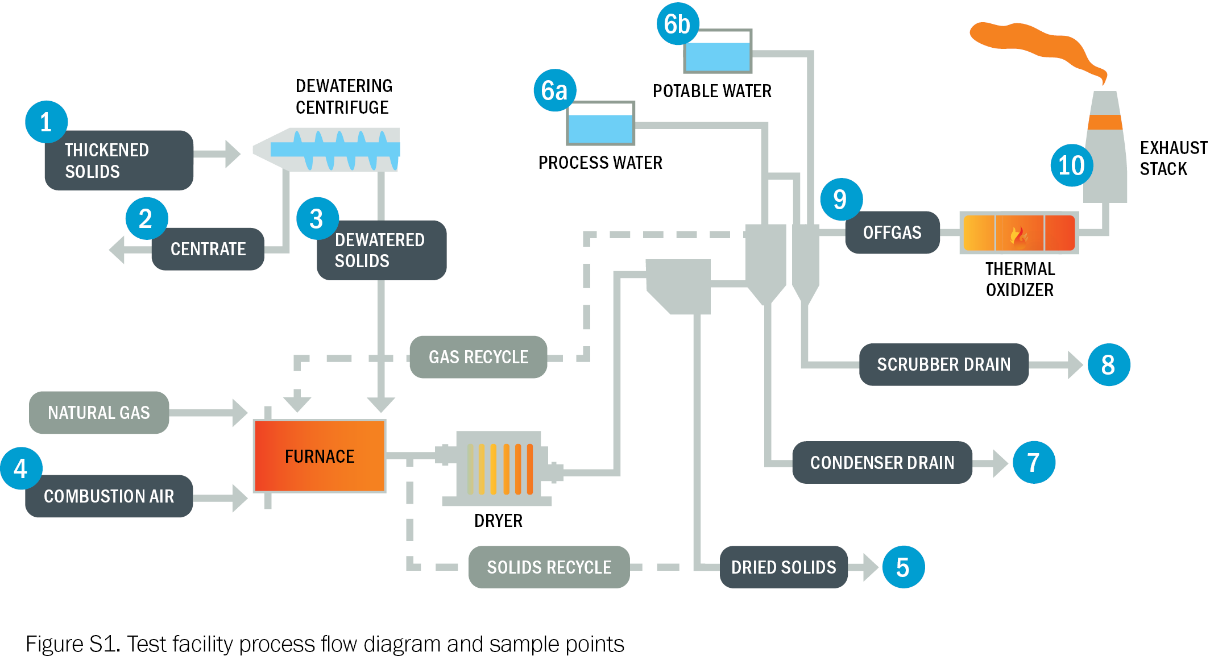


Figure S-1 Test Facility Process Flow Diagram and Sample Points

The process begins with thickened solids (1.4% - 1.7% total solids [TS]), which are conditioned with polymer and then dewatered to 22% - 23% total solids (TS). The dewatered solids are fed to a rotary drum dryer. This facility specifically uses a single Andritz DDS40 dryer train for the drying process, where 95%-99% of the remaining water is evaporated, resulting in dried solids collected as pellets. The dried solids are screened with over and under-sized product recycled internally to improve dryer feed quality. Screened final product is cooled and loaded out via pellet silos. The dryer exhaust is treated through the condenser for moisture, and most of the volume (approximately 80%) is recycled back to the dryer furnace with the balance sent to the Venturi scrubber for particulate removal and RTO for final treatment and discharge. More detail is provided for the emissions control elements as follows:

- Tray condenser/sub-cooler to cool the exhaust gas from 93°C to 49°C, cooling and dropping out evaporated moisture and removing most of the residual fine particulate matter, condensable volatile materials (VOMs) and ammonia.
- Venturi scrubber to remove micron sized particulates.
- RTO for thermal destruction or oxidation of odor compounds, carbon monoxide and remaining VOMs.

Table S-1 summarizes the operating parameters for the air pollution control (APC) devices based on typical operating conditions for a DDS40 dryer.

Table S-1. Test Site Operating Parameters

| Order | APC Device | Operating Temperatures | Inputs | Outputs |
| --- | --- | --- | --- | --- |
| 1 | Condenser | 93°C Inlet 50°C Outlet | ~25,000 ACFM Process Gas  350 gpm plant water | Process Gas  Condenser Drain |
| 2 | Venturi Scrubber | 50°C Inlet and Outlet | ~5,000 ACFM Process Gas  50 gpm plant water  55 gpm potable water | Process Gas  Scrubber Drain |
| 3 | RTO | 815-870°C combustion | ~5,000 ACFM Process Gas  Combustion Air | Exhaust Stack Outlet |

# S2. Sampling and Analytical Details

The physico-chemical properties of PFAS (charge state, polarity, volatility) drive the selection of analytical procedures. The analytical program fully aimed to characterize the PFAS content of each input/output stream from the processing systems. A strict mass balance is not possible with today’s analytical techniques due to the PFAS family including over 8,000 compounds and variety of properties, though a mass balance can be attempted on the quantifiable compounds.

We have used the following analytical techniques to track certain compounds and identify others present in a multi-platform approach:

- Targeted analysis
- Total organic fluorine (TOF)

We have deliberately excluded the total oxidizable precursor (TOP) assay from this program. The technique converts unquantifiable PFAS into the forms that can be quantified using targeted analyses. The method does require the targeted analysis to be conducted twice, once before and once after the conversion step, to identify the unquantifiable PFAS. Furthermore, the conversion process may not fully convert all PFAS to quantifiable form.

## S2.1 Source Air (Stack)

The seven subsamples gathered using the Method OTM-45 sampling train are consolidated into four analytical samples. The segments of the sampling train that correspond to these four aggregated fractions are visually depicted in Figure S-2.

- Filter and front half rinses
- Front XAD with back half rinses
- Impinger content and rinses
- Breakthrough XAD


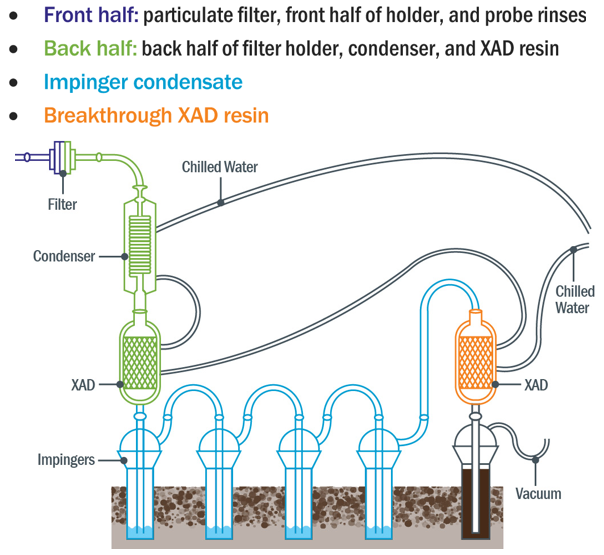


Figure S-2. Method OTM-45 sampling train analytical fractions.

Stack sampling followed several USEPA methods to measure flow rate (USEPA 2020 and USEPA 2017a), oxygen and carbon dioxide (USEPA 2017b), and moisture content (USEPA 2017c). The emission testing program was conducted in accordance with the test methods listed in Table S-2. Method descriptions are provided below as written by Alliance Source Testing (AST).

**Table S-2. Source Air and Combustion Air Sampling Methodology.**

| Parameter | USEPA Reference Test Methods | Notes/Remarks |
| --- | --- | --- |
| Volumetric Flow Rate | 1 & 2 | Full Velocity Traverses |
| Oxygen/Carbon Dioxide | 3A | Instrumental Analysis |
| Moisture Content | 4 | Gravimetric Analysis |
| PFAS | Modified 0010 (OTM-45) | Isokinetic Sampling (Exhaust)  Constant Rate (Combustion Air) |

### USEPA Reference Test Methods 1 and 2 — Volumetric Flow Rate

The sampling location and number of traverse (sampling) points were selected in accordance with USEPA Reference Test Method 1. To determine the minimum number of traverse points, the upstream and downstream distances were equated into equivalent diameters and compared to Figure 1-1 in USEPA Reference Test Method 1.

Full velocity traverses were conducted in accordance with USEPA Reference Test Method 2 to determine the average stack gas velocity pressure, static pressure and temperature. The velocity and static pressure measurement system consisted of a pitot tube and inclined manometer. The stack gas temperature was measured with a K-type thermocouple and pyrometer.

Stack gas velocity pressure and temperature readings were recorded during each test run. The data collected were utilized to calculate the volumetric flow rate in accordance with USEPA Reference Test Method 2.

### USEPA Reference Test Method 3/3A — Oxygen/Carbon Dioxide

The oxygen and carbon dioxide testing were conducted in accordance with USEPA Reference Test Method 3A. Data was collected online and reported in one-minute averages. The sampling system consisted of a stainless-steel probe, Teflon sample line(s), gas conditioning system and the identified gas analyzer. The gas conditioning system was a non-contact condenser used to remove moisture from the stack gas. If an unheated Teflon sample line was used, then a portable non-contact condenser was placed in the system directly after the probe. Otherwise, a heated Teflon sample line was used.

#### USEPA Protocol 1 Calibration Gases

Cylinder calibration gases with known concentrations of CO2 and O2 used met USEPA Protocol 1 (+/- 2 percent) standards. Copies of all calibration gas certificates are available.

#### Direct Calibration & Calibration Error Test

Low Level gas was introduced directly to the analyzer. After adjusting the analyzer to the Low-Level gas concentration and once the analyzer reading was stable, the analyzer value was recorded. This process was repeated for the High-Level gas. For the Calibration Error Test, Low- (less than 20 percent of calibration span), Mid- (between 40 to 60 percent of calibration span), and High (equal to calibration span)-Level calibration gases were sequentially introduced directly to the analyzer. All values were within 2.0 percent of the Calibration Span or 0.5% absolute difference.

#### System Bias and Response Time

High- or Mid-Level gas (whichever was closer to the stack gas concentration) was introduced at the probe and the time required for the analyzer reading to reach 95 percent or 0.5 percent (whichever was less restrictive) of the gas concentration was recorded. The analyzer reading was observed until it reached a stable value, and this value was recorded. Next, Low-Level gas was introduced at the probe and the time required for the analyzer reading to decrease to a value within 5.0 or 0.5 percent (whichever was less restrictive) was recorded. If the Low-Level gas was zero gas, the response was 0.5 or 5.0 percent of the upscale gas concentration (whichever was less restrictive). The analyzer reading was observed until it reached a stable value and this value was recorded. The measurement system response time and initial system bias were determined from these data. The System Bias was within 5.0 percent of the Calibration Span or 0.5 percent absolute difference.

#### Post Test System Bias Checks

High- or Mid-Level gas (whichever was closer to the stack gas concentration) was introduced at the probe. After the analyzer response was stable, the value was recorded. Next, Low-Level gas was introduced at the probe, and the analyzer value recorded once it reached a stable response. The System Bias was within 5.0 percent of the Calibration Span or 0.5 percent absolute difference, or the data were invalidated and the Calibration Error Test and System Bias were repeated.

#### Post Test Drift Checks

Drift between pre- and post-run System Bias was within 3 percent of the Calibration Span or 0.5 percent absolute difference. If the drift exceeded 3 or 0.5 percent, the Calibration Error Test and System Bias were repeated.

#### Data Collection

A Data Acquisition System with battery backup was used to record the instrument response in one (1) minute averages. The data was continuously stored as a *.CSV file in Excel format on the hard drive of a computer. At the completion of testing, the data was also saved to the AST server. All data was reviewed by the Field Team Leader before leaving the facility. Once arriving at AST’s office, all written and electronic data was relinquished to the report coordinator and then a final review was performed by the Project Manager.

### USEPA Reference Test Method 4 — Moisture Content

The stack gas moisture content was determined in accordance with USEPA Reference Test Method 4. The gas conditioning train consisted of a series of chilled impingers. Prior to testing, each impinger was filled with a known quantity of water or silica gel. Each impinger was analyzed gravimetrically before and after each test run on the same balance to determine the amount of moisture condensed.

### USEPA Other Test Method 45 (OTM-45) — PFAS

PFAS emissions were evaluated in accordance with Method OTM-45. Testing followed the work plan developed by Brown and Caldwell.

The sample train consisted of a borosilicate glass nozzle attached directly to a heated borosilicate glass-lined probe. The probe was connected directly to a heated borosilicate glass filter holder containing a solvent-extracted glass fiber filter. To minimize possible thermal degradation of the HFPO-DA, the probe and particulate filter were heated to just above stack temperature to minimize water vapor condensation before the filter. The filter holder exit was connected to a water-cooled coil condenser followed by a water-cooled sorbent module containing approximately 40 grams of XAD-2 resin. The XAD-2 inlet temperature was monitored to ensure that the module was maintained at a temperature below 20°C.

The XAD-2 resin trap was followed by a condensate knockout impinger and a series of two impingers each containing 100-mL of high purity deionized water. The water impingers were followed by another series of two condensate knockout impingers equipped with a second XAD-2 resin trap to account for any sample breakthrough. The final impinger contained approximately 250 grams of dry pre-weighed silica gel. The water impingers and condensate impingers were submerged in an ice bath through the duration of the testing. The water in the ice bath was also used to circulate around the coil condenser and the XAD-2 resin traps.

Exhaust gases were extracted from the sample locations isokinetically using a metering console equipped with a vacuum pump, a calibrated orifice, oil manometer and probe/filter heat controllers.

## S2.2 Combustion Air

The sample train at the combustion air location consisted of a XAD-2 resin trap followed by a condensate knockout impinger as shown in Figure S-3. The samples were collected at a constant rate concurrently with the exhaust samples.


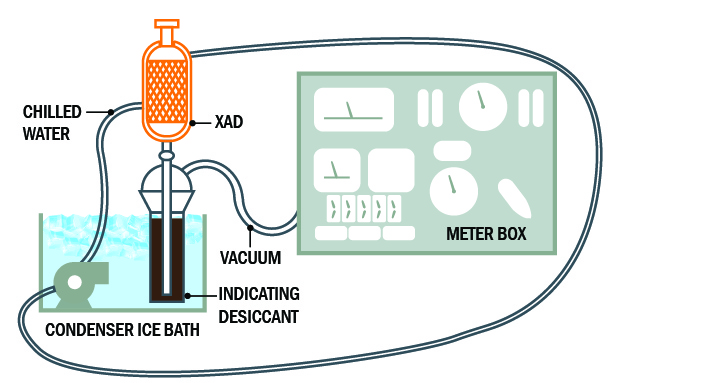


Figure S-3. Combustion air sampling scheme.

## S2.3 Targeted Polar Analytes

Table S-3 contains the polar PFAS targeted/quantified for this project.

Table S-3. Targeted Polar PFAS Analytes**.**

| Family | Full Name | Acronym | CAS | Molecular Weight (g/mol)  of acid form |
| --- | --- | --- | --- | --- |
| Cyclic PFAS Ether sulfonic acids | Perfluoroethylcyclohexane Sulfonate | PFECHS | 646-83-3 | 462.13 |
| Per/poly- fluorinated ether sulfonic acids (PFESA) | 9-chlorohexadecafluoro-3-oxanonane-1-sulfonic acid | 9Cl-PF3ONS (F53B major) | 756426-58-1 | 532.58 |
|  | 11-chloroeicosafluoro-3-oxaundecane-1-sulfonic acid | 11Cl-PF3OUdS (F53B minor) | 763051-92-9 | 632.6 |
| Fluorotelomer carboxylic acids (FTCA) | 3-Perfluorohexanoic acid | 3:3 FTCA | 356-02-5 | 242.1 |
|  | 3-Perfluoropentylpropanoic acid | 5:3 FTCA | 914637-49-3 | 342.11 |
|  | 6:2 Fluorotelomer carboxylic acid | 6:2 FTCA | 53826-12-3 | 378.09 |
|  | 3-Perfluoroheptylpropanoic acid | 7:3 FTCA | 812-70-4 | 442.12 |
|  | 8:2 Fluorotelomer carboxylic acid | 8:2 FTCA | 27854-31-5 | 478.1 |
|  | 10:2 Fluorotelomer carboxylic acid | 10:2 FTCA | 53826-13-4 | 578.12 |
| Fluorotelomer sulfonic acids (FTSA) | 1H,1H,2H,2H-perfluorohexane sulfonic acid | 4:2 FTS | 757124-72-4 | 328.15 |
|  | 1H,1H,2H,2H-perfluorooctane sulfonic acid | 6:2 FTS | 27619-97-2 | 428.17 |
|  | 1H,1H,2H,2H-perfluorodecane sulfonic acid | 8:2 FTS | 39108-34-4 | 528.18 |
|  | 1H,1H,2H,2H-perfluorododecane sulfonic acid | 10:2 FTS | 120226-60-0 | 628.2 |
| Fluorotelomer unsaturated carboxylic acids | 6:2 Fluorotelomer unsaturated carboxylic acid | 6:2 FTUCA | 70887-88-6 | 358.08 |
|  | 8:2 Fluorotelomer unsaturated carboxylic acid | 8:2 FTUCA | 70887-84-2 | 458.1 |
| Per- and Polyfluoroether carboxylic acids | Perfluoro-3-methoxypropanoic acid | PFMPA | 377-73-1 | 230.04 |
|  | Perfluoro-4-methoxybutanoic acid | PFMBA | 863090-89-5 | 280.04 |
|  | Nonafluoro-3,6-dioxaheptanoic acid | NFDHA | 151772-58-6 | 296.04 |
|  | Hexafluoropropylene oxide dimer acid | HFPODA (GenX) | 13252-13-6 | 330.05 |
|  | 4,8-Dioxa-3H-perfluorononanoic acid | DONA | 919005-14-4 | 378.07 |
| Perfluoroalkane carboxylic acids (PFCA) | Perfluorobutanoic acid | PFBA | 375-22-4 | 214.04 |
|  | Perfluoropentanoic acid | PFPeA | 2706-90-3 | 264.05 |
|  | Perfluorohexanoic acid | PFHxA | 307-24-4 | 314.05 |
|  | Perfluoroheptanoic acid | PFHpA | 375-85-9 | 364.06 |
|  | Perfluorooctanoic acid | PFOA | 335-67-1 | 414.07 |
|  | Perfluorononanoic acid | PFNA | 375-95-1 | 464.08 |
|  | Perfluorodecanoic acid | PFDA | 335-76-2 | 514.08 |
|  | Perfluoroundecanoic acid | PFUnA | 2058-94-8 | 564.09 |
|  | Perfluorododecanoic acid | PFDoA | 307-55-1 | 614.1 |
|  | Perfluorotridecanoic acid | PFTrDA | 72629-94-8 | 664.11 |
|  | Perfluorotetradecanoic acid | PFTeDA | 376-06-7 | 714.11 |
|  | Perfluoro-n-hexadecanoic acid | PFHxDA | 67905-19-5 | 814.13 |
|  | Perfluoro-n-octadecanoic acid | PFODA | 16517-11-6 | 914.14 |
| Perfluoroalkane sulfonic acids (PFSA) | Perfluoro-(2-ethoxyethane)-sulfonic acid | PFEESA/PES | 113507-82-7 | 316.1 |
|  | Perfluorobutanesulfonic acid | PFBS | 375-73-5 | 300.1 |
|  | Perfluoropentanesulfonic acid | PFPeS | 2706-91-4 | 350.11 |
|  | Perfluorohexanesulfonic acid | PFHxS | 355-46-4 | 400.12 |
|  | Perfluoroheptanesulfonic acid | PFHpS | 375-92-8 | 450.12 |
|  | Perfluorooctanesulfonic acid | PFOS | 1763-23-1 | 500.13 |
|  | Perfluorononanesulfonic acid | PFNS | 68259-12-1 | 550.14 |
|  | Perfluorodecanesulfonic acid | PFDS | 335-77-3 | 600.15 |
|  | Perfluorododecanesulfonic acid | PFDoS | 79780-39-5 | 700.16 |
| Perfluoroalkane sulfonamido ethanol (FASE) | 2-(N-methylperfluoro-1-octanesulfonamido) ethanol | NMeFOSE | 24448-09-7 | 557.23 |
|  | 2-(N-ethylperfluoro-1-octanesulfonamido) ethanol | NEtFOSE | 1691-99-2 | 571.25 |
| Perfluoroalkane sulfonamide (FASA) | Perfluorooctanesulfonamide | FOSA | 754-91-6 | 499.15 |
|  | N-methylperfluorooctane sulfonamide | NMeFOSA | 31506-32-8 | 513.169 |
|  | N-ethylperfluorooctane sulfonamide | NEtFOSA | 4151-50-2 | 527.2 |
| Perfluoroalkane sulfonamido acetic acid (FASAA) | N-methylperfluoro-1-octanesulfonamido acetic acid | NMeFOSAA | 2355-31-9 | 571.21 |
|  | N-ethylperfluorooctanesulfonamidoacetic acid | NEtFOSAA | 2991-50-6 | 585.24 |

## S2.4 TOF

The analysis using the TOF assay was recommended if the PFAS product composition was unknown; where known PFAS composition extended beyond the standardized LC-MS/MS analytes; where there was likely to be transformation of PFAS; or, where precursors were unknown (HEPA, 2020). TOF was not specific to chain length and did not target specific PFAS precursors (HEPA, 2020). TOF yielded the mass of fluorine present in the sample, which was then compared with the organic fluorine equivalent detected by the USEPA standard method (HEPA, 2020). Results of TOF analyses had indicated that significant amounts of organic fluorine compounds were unaccounted for by targeted analyses (Liu et al., 2019 and Winchell et al., 2024).

The application of surrogate analyses was an attempt to determine the “real” concentration of PFAS in a sample. The disadvantage of TOF analyses was that the identities of specific PFAS or precursors present in a sample remained unknown. In practice, these screening methods were intended to more completely indicate the PFAS in a sample by comparison of TOF results with standardized LC-MS/MS analyses. Similar results indicated the absence of substantial precursors, whereas divergence in results suggested that large quantities of precursors were present that were undetectable by standard quantitative analyses (HEPA, 2020).

The TOF analysis was an indicator of the total fluorinated organic content in a sample. Briefly, PFAS were adsorbed, most often on activated carbon, or other sorbents, after which, combustion was used to release the fluoride ion (F−) (HEPA, 2020; Trojanowicz and Koc, 2013; Mills et al., 2020). Removing the inorganic F− was important so that only organic F− was measured. Combustion at 900 to 1000°C released the fluorine followed by determination of fluoride concentration by ion chromatography and the combined process was termed CIC (Eurofins, 2018; Trojanowicz and Koc, 2013). At the time of these analyses, a standard method did not exist for TOF. Because TOF measured the total mass of fluorine (ng□L^-1^), background inorganic fluoride ion (F−) and organic fluorine compounds other than PFAS (such as pharmaceuticals and herbicides) could influence TOF results (HEPA, 2020; Mills et al., 2020). Using different sample preparation techniques, either total fluorine (organic plus inorganic) or extractable organofluorine (EOF) could be determined (Joudan et al., 2020). Depending on the media sampled, other adaptations of the TOF assay had been tested, such as adsorbable organofluorine (AOF) or EOF analyses (Eurofins, 2018; Nakayama et al., 2019; Trojanowicz and Koc, 2013). The study utilized the proprietary methods developed by ETA. Exact procedures were documented.

The extracts were treated with calcium hydroxide to precipitate hydrogen fluoride as it acted as an interference during the CIC analysis.

ETA analyzed each sample point shown in Figure S-1 for TOF. Reported units matched those listed for the targeted analyses.

## S2.5 Solids Characteristics

TSS, TS, and VS analysis were conducted by Eurofins, using Standard Method (SM) 2540D and G.

## S2.6 Sampling Event Details

General sampling event protocols included the following:

The dryer train and supporting processes were operated “normally” and consistently over the sampling periods. The main goal was to not experience a system shutdown or otherwise change in operation that could impact the representativeness of the samples and subsequent analytical results. A coincidental benefit was the operation of the system at “normal” conditions generally reflects where peer facilities operate, thus providing relatable PFAS performance.

Within the range of “normal” operations, the dewatered solids feed rate was maximized to increase the expected PFAS load onto the system and increase the likelihood of measuring reportable levels in the samples.

The sampling event took three days. The following outline summarizes the schedule:

Day 1 – BC and subcontractors arrived on site to set up and coordinate the subsequent sample event with test site staff.

Day 2 AM – The first sample was collected.

Day 2 PM – The second sample was collected.

Day 3 AM – The third sample was collected.

Day 3 PM – Tear down and coordination of samples took place.

On Day 1 of the sampling event, BC and subcontractors toured the site to identify the sampling locations. At that time, the combustion air sample location was determined. All other sample points had been previously established and test site staff confirmed their operability prior to the sampling event.

Prior to the sampling event, BC coordinated with the WRRF staff to clear any logistical requirements. These included security clearances, health and safety documentation, COVID-19 protocols, or otherwise.

Table S-4 identified the samples collected over the sampling event. These were the samples received by ETA. ETA may have split the samples for distribution to various laboratories supporting specific analyses.

Table S-4. Sample List

| Stream | Sample Point (Figure S-1) | Collection Type | Samples |
| --- | --- | --- | --- |
| Thickened Solids | **1** | **Composited grabs** | **3** |
| Centrate | **2** | **Composited grabs** | **3** |
| Dewatered Solids | **3** | **Composited grabs** | **3 + 1 field duplicate** |
| Combustion air | **4** | **Custom** | **3** |
| Dried Product | **5** | **Composited grabs** | **3 + 1 field duplicate** |
| Cooling water supply (process) | **6** | **Composited grabs** | **3** |
| Cooling water supply (potable) | **6** | **Composited grabs** | **3** |
| Process drain (Condenser) | **7** | **Composited grabs** | **3** |
| Process drain (scrubber) | **8** | **Composited grabs** | **3 + 1 field duplicate** |
| Dryer Exhaust (pre- and post-RTO, single sample train described for clarity) | **9, 10** | **Method OTM-45** | **6 particulate filters**  **6 front half methanol/NH4OH rinse**  **6 front XAD cartridges**  **6 back half methanol/NH4OH rinse**  **6 impinger condensate**  **6 impinger methanol/NH4OH rinse**  **6 breakthrough XAD cartridges**  **1 particulate filter field blank**  **1 front half methanol/NH4OH field blank**  **1 front XAD cartridge field blank**  **1 back half methanol/NH4OH field blank**  **1 impinger condensate field blank**  **1 impinger methanol/NH4OH field blank**  **1 breakthrough XAD cartridge field blank**  **1 particulate filter proof blank**  **1 front half methanol/NH4OH proof blank**  **1 front XAD cartridge proof blank**  **1 back half methanol/NH4OH proof blank**  **1 impinger condensate proof blank**  **1 impinger methanol/NH4OH proof blank**  **1 breakthrough XAD cartridge proof blank** |
| Total Samples | **--** | **--** | **86** |

## S2.7 Data Quality Standards

Project analytical data were assessed for data quality using several means: (1) the Quality Assurance/Quality Control (QA/QC) data provided by the analytical laboratory partners, (2) the Brown and Caldwell data verification and validation guidelines for reporting general chemistry parameters; (3) guidance documents from USEPA (2002) and Interstate Technology & Regulatory Council (ITRC, 2022a and 2022b); (4) though not yet promulgated, guidance from USEPA Draft Method 1633 (USEPA, 2022b) and USEPA Draft Method 1621 (USEPA, 2022a) was considered; and (5) the experience/expertise of the project research team.

The four-step assessment process applied to the data gathered in this project proceeded through the stages documented by ITRC (2022a). Assessment of data usability was the final step after verification, validation, and establishment of data quality. The laboratories used in this project provided extensive quality control information in the form of data qualifiers for the analyses reported. The research team reviewed the data to verify and validate the results to assess the overall data quality. Finally, the data qualifiers were interpreted by the team to assess data usability. A list of qualifiers and definitions encountered and their interpretation for the determination of data usability in this project is presented in Table S-5.

Among the most important data quality parameters is the sensitivity of the analysis. Numerous terms and acronyms have evolved in the literature to describe “detection” and “quantitation” limits (USEPA, 1995 and ITRC, 2022a). In these data, the terms minimum detection limit (MDL) and reporting limit (RL) are used to report the “detection” and “quantitation” limits, respectively. The MDL is defined as the minimum measured concentration of a substance that can be reported with 99 percent confidence that the measured concentration is distinguishable from method blank results (USEPA, 2017d). The RL is the lowest nonzero calibration point in the calibration curve for each analyte. Because of varying properties between samples, that is, sample size, matrix effects, dilutions made during analysis, the RL can vary from sample to sample and analyte to analyte (ITRC, 2022b). The RL values for each analyte in each sample are provided in the data tables within this SI.

The “J” qualifier represents an estimated analytical result between the MDL and the RL. The project team determined that although the numerical value having a J qualifier as reported by the laboratory was a valid indicator of data “quality,” the J qualifier, represents an estimation outside the calibration curve, and therefore, its data “usability” is unfit. Data having J qualifiers, regardless of other qualifiers listed for the sample, were not reported numerically but were reported as “J.” No numerical values were reported for the data qualifiers I, CI, *+, and *1; the numerical value was replaced by the data qualifier itself, alerting the data user that the result could not be confirmed.

The “B” qualifier was assessed differently for the chromatographic quantitative data versus the surrogate qualitative data based on the not yet promulgated, guidance from USEPA Draft Method 1633 (USEPA, 2022b) and Method 1621 (USEPA, 2022a):

- USEPA Draft Method 1633: Analysis of Per- and Polyfluoroalkyl Substances (PFAS) in Aqueous, Solid, Biosolids, and Tissue Samples by LC-MS/MS. “Results associated with blank contamination for an analyte regulated in a discharge cannot be used to demonstrate regulatory compliance.”
- USEPA Draft Method 1621: Screening Method for the Determination of Adsorbable Organic Fluorine (AOF) in Aqueous Matrices by Combustion Ion Chromatography (CIC). “EPA has classified this procedure as a screening method that may be used to estimate the aggregate contributions of the organofluorine compounds in the sample. Because of the common occurrence of background levels of fluoride in the sorption media, the method subtracts the amount of fluoride observed in the method blank from the fluoride in the sample to estimate the AOF attributable to the sample itself.”

For the B data qualifier associated with the chromatographic quantitative data, no numerical values were reported; the numerical value was replaced by the data qualifier itself, alerting the data user that the result could not be confirmed. For the B data qualifier associated with the surrogate qualitative data in TOF analyses, the numerical value was reported accompanied by a B superscript. For the TOF samples, samples and method blank contains isotope dilution analysis (IDA) and surrogate standards necessitating the subtraction of the method blank from the sample.

**Table S-5. Judgement Criteria on Data Usability.**

| Data Qualifiers | Assessment of Data Usability | Data Reported As | Data Determined unusable |
| --- | --- | --- | --- |
| ND | Not detected at the MDL. | <MDL | X |
| J | Result is less than the RL but greater than or equal to the MDL and the concentration is an approximate value. Qualifier indicates quantitation outside the calibration range. The result is estimated/uncertain with indeterminate bias. | J (no numerical value reported) | X |
| I | Value is estimated maximum possible concentration (EMPC). Interference. | I (no numerical value reported) | X |
| CI | The peak identified by the data system exhibited chromatographic interference that could not be resolved. There is reason to suspect there may be a high bias. | CI (no numerical value reported) | X |
| *+ | Lab Control Sample (LCS) and/or Lab Control Sample Duplicate (LCSD) is outside acceptance limits, high biased. | *+ (no numerical value reported) | X |
| *- | Lab Control Sample (LCS) and/or Lab Control Sample Duplicate (LCSD) is outside acceptance limits, low biased. | *+ (no numerical value reported) | X |
| *1 | LCS/LCSD Relative Percent Difference (RPD) exceeds control limits. | *1 (no numerical value reported) | X |
| B (chromatographic quantitative data | Compound was found in the blank and sample. | B (no numerical value reported) | X |
| B (surrogate qualitative data, i.e., TOF) | Compound was found in the blank and sample. Screening analysis. Samples and method blank contains IDA and surrogate standards. Data determined to be usable. | Numerical value reported with B superscript |  |
| H (surrogate qualitative data, i.e., TOF) | Sample was prepped or analyzed beyond the specified holding time. Holding times have not been established. | Numerical value reported with H superscript |  |

# **S3. Operating Data During Sampling Event**

Biosolids dewatering and drying systems include extensive instrumentation throughout the process. The continuous data collected by these systems was logged in the test site's historian system and available in time-based increments. Key, but not all-inclusive, data included:

- Thickened and dewatered solids feed rates.
- Dryer furnace and rotary drum temperature profiles.
- Process air flow rates.
- Cooling water flow rates (supply and drain) and scrubber system pressure drops.
- Natural gas flow rates.
- RTO operating temperatures and oxygen contents.

Average loading rates for the solid streams for each experimental run are provided in Table S-6.

**Table S-6. Solid Samples Flowrates.**

| Parameter | Units | Dewatered Solids | Dried Solids |
| --- | --- | --- | --- |
| Loading Rate | *Wet lb/day* | 349,272 | 116,424 |

Average flow rates for the liquid sample points for each experimental run are provided in Table S-7.

**Table S-7. Liquid Samples Flowrates.**

| Parameter | Units | Thickened Solids | Centrate | Cooling Water (Potable) | Cooling Water (Process) | Process Drain (Condenser) | Process Drain (Scrubber) |
| --- | --- | --- | --- | --- | --- | --- | --- |
| Flowrate | *gpm* | 308.9 | 284.3 | 10 | 497 | 519 | 10 |

Relevant average operating conditions for the gas sample points for each experimental run are provided in Table S-8. Note that the flowrate at the stack is substantially higher than the RTO Inlet as the stack also receives ventilation air from the thickened solids storage tank. It was assumed that PFAS atmospheric contribution from this air stream was negligible.

**Table S-8. Gas Phase Operating Conditions.**

| Sample | Test Duration | Temperature | Flowrate |
| --- | --- | --- | --- |
| *Units* | *Minutes* | *°F* | *DSCFM* |
| Combustion Air | 240 | 74.4 | N/A |
| RTO Inlet | 240 | 105.6 | 4,630 |
| RTO Outlet (Stack) | 240 | 98.7 | 27,746 |

Average operating conditions for the dryer, scrubber system, and RTO are provided in Table S-9.

**Table S-9. Dryer, Scrubber and RTO Operating Conditions.**

| Parameter | *Imperial Unit* | Value | *SI Unit* | Value |
| --- | --- | --- | --- | --- |
| Drum Inlet Temperature | *°F* | 670 | *°C* | 355 |
| Furnace Pressure | *" WC* | -0.82 | *PSI* | -0.03 |
| Dryer Natural Gas Flow | *SCF/hr* | 104,990 | *SCM/hr* | 2,973 |
| Cooling Water Flow | *Gpm* | 273 | *Liter/min* | 1,033 |
| Condenser (Saturator) Differential Pressure | *%* | 18.1 | *%* | 18.1 |
| RTO Burner (1 Through 3) Temperature | *°F* | 1,555 | *°C* | 846 |
| RTO Burners Natural Gas Flow | *SCF/hr* | 20.13 | *SCM/hr* | 0.57 |
| Dewatered Solids Content | *%TS* | 22 | *%TS* | 22 |
| Pellet Solids Content | *%TS* | 94 | *%TS* | 94 |
| Water Evaporation | *Lb/hr* | 8,313 | *kg/hr* | 3770 |

TS data was used for this evaluation and test site staff collected this information daily. BC coordinated with the test site to obtain this data. Physical characteristics of the dryer system were gathered from as-builts and consultation with equipment suppliers as needed to estimate residence times and operating ranges.


# S4. Results

The following summarizes the results of the sample analyses. Full data results are provided in Tables S-10 through S-12, PFAS flows on a mass basis are provided in Tables S-13 through S-11, average sum PFAS detected on a molar basis are presented in Table S-16, OTM-45 QC data is presented in Table S-17, and reporting limit data is presented in Tables S-18 through S-23. More detail and raw data are available upon request to the corresponding author.

| Table S-10: Dryer System Input Samples – Full Results | | | | | | | | | | | |
| --- | --- | --- | --- | --- | --- | --- | --- | --- | --- | --- | --- |
|  |  | **Thickened Solids (Liquid Fraction)** | | **Thickened Solids (Solid Fraction)** | | **Dryer** Combustion **Air** | | **Cooling Water Supply (Process)** | | **Cooling Water Supply (Potable)** | |
|  |  | **Concentration** | **Emission** | **Concentration** | **Emission** | **Concentration** | **Emission** | **Concentration** | **Emission** | **Concentration** | **Emission** |
|  | **Units** | **ng/L** | **mg/run** | **ng/g** | **mg/run** | **ng/Sample** | **mg/run** | **ng/L** | **mg/run** | **ng/L** | **mg/run** |
| **PFAS Family** | **Acronym** | ***(R1/R2/R3)*** | ***(R1/R2/R3)*** | ***(R1/R2/R3)*** | ***(R1/R2/R3)*** | ***(R1/R2/R3)*** | ***(R1/R2/R3)*** | ***(R1/R2/R3/R4)*** | ***(R1/R2/R3/R4)*** | ***(R1/R2/R3/R4)*** | ***(R1/R2/R3/R4)*** |
| **Cyclic PFAS** | **PFECHS** | NA/NA/NA | NA/NA/NA | NA/NA/NA | NA/NA/NA | ND/ND/ND | --/--/-- | NA/NA/NA/NA | NA/NA/NA/NA | NA/NA/NA/NA | NA/NA/NA/NA |
| **Ether sulfonic acids** | **9Cl-PF3ONS** | ND (RL: 200)/ND (RL: 200)/ND (RL: 200) | --/--/-- | ND/ND/ND | --/--/-- | ND/ND/ND | --/--/-- | ND/ND/ND/ND | --/--/--/Q | ND/ND/ND/ND | --/--/--/Q |
|  | **11Cl-PF3OUdS** | ND (RL: 200)/ND (RL: 200)/ND (RL: 200) | --/--/-- | ND/ND/ND | --/--/-- | *-/*-/*- | --/--/-- | ND/ND/ND/ND | --/--/--/Q | ND/ND/ND/ND | --/--/--/Q |
| **Fluorotelomer carboxylic acids** | **3:3 FTCA** | NA/NA/NA | NA/NA/NA | NA/NA/NA | NA/NA/NA | ND/ND/ND | --/--/-- | NA/NA/NA/NA | NA/NA/NA/NA | NA/NA/NA/NA | NA/NA/NA/NA |
|  | **5:3 FTCA** | NA/NA/NA | NA/NA/NA | NA/NA/NA | NA/NA/NA | *+/*+/*+ | --/--/-- | NA/NA/NA/NA | NA/NA/NA/NA | NA/NA/NA/NA | NA/NA/NA/NA |
|  | **6:2 FTCA** | NA/NA/NA | NA/NA/NA | NA/NA/NA | NA/NA/NA | ND/ND/ND | --/--/-- | NA/NA/NA/NA | NA/NA/NA/NA | NA/NA/NA/NA | NA/NA/NA/NA |
|  | **7:3 FTCA** | NA/NA/NA | NA/NA/NA | NA/NA/NA | NA/NA/NA | ND/ND/ND | --/--/-- | NA/NA/NA/NA | NA/NA/NA/NA | NA/NA/NA/NA | NA/NA/NA/NA |
|  | **8:2 FTCA** | NA/NA/NA | NA/NA/NA | NA/NA/NA | NA/NA/NA | ND/ND/ND | --/--/-- | NA/NA/NA/NA | NA/NA/NA/NA | NA/NA/NA/NA | NA/NA/NA/NA |
|  | **10:2 FTCA** | NA/NA/NA | NA/NA/NA | NA/NA/NA | NA/NA/NA | ND/ND/ND | --/--/-- | NA/NA/NA/NA | NA/NA/NA/NA | NA/NA/NA/NA | NA/NA/NA/NA |
| **Fluorotelomer sulfonic acids** | **4:2 FTS** | ND (RL: 200)/ND (RL: 200)/ND (RL: 200) | --/--/-- | ND/ND/ND | --/--/-- | ND/ND/ND | --/--/-- | ND/ND/ND/ND | --/--/--/Q | ND/ND/ND/ND | --/--/--/Q |
|  | **6:2 FTS** | ND (RL: 500)/ND (RL: 500)/ND (RL: 500) | --/--/-- | ND/ND/ND | --/--/-- | ND/ND/ND | --/--/-- | ND/ND/ND/ND | --/--/--/Q | ND/ND/ND/ND | --/--/--/Q |
|  | **8:2 FTS** | ND (RL: 300)/ND (RL: 300)/ND (RL: 300) | --/--/-- | ND/ND/ND | --/--/-- | <RL (J)/<RL (J)/<RL (J) | --/--/-- | ND/ND/ND/ND | --/--/--/Q | ND/ND/ND/ND | --/--/--/Q |
|  | **10:2 FTS** | ND (RL: 500)/ND (RL: 500)/ND (RL: 500) | --/--/-- | ND/ND/<RL (J) | --/--/-- | ND/ND/ND | --/--/-- | ND/ND/ND/ND | --/--/--/Q | ND/ND/ND/ND | --/--/--/Q |
| **Fluorotelomer unsaturated carboxylic acids** | **6:2 FTUCA** | NA/NA/NA | NA/NA/NA | NA/NA/NA | NA/NA/NA | ND/ND/ND | --/--/-- | NA/NA/NA/NA | NA/NA/NA/NA | NA/NA/NA/NA | NA/NA/NA/NA |
|  | **8:2 FTUCA** | NA/NA/NA | NA/NA/NA | NA/NA/NA | NA/NA/NA | ND/ND/ND | --/--/-- | NA/NA/NA/NA | NA/NA/NA/NA | NA/NA/NA/NA | NA/NA/NA/NA |
| **Per- and Polyfluoroether carboxylic acids** | **PFMPA** | NA/NA/NA | NA/NA/NA | NA/NA/NA | NA/NA/NA | ND/ND/ND | --/--/-- | NA/NA/NA/NA | NA/NA/NA/NA | NA/NA/NA/NA | NA/NA/NA/NA |
|  | **PFMBA** | NA/NA/NA | NA/NA/NA | NA/NA/NA | NA/NA/NA | ND/ND/ND | --/--/-- | NA/NA/NA/NA | NA/NA/NA/NA | NA/NA/NA/NA | NA/NA/NA/NA |
|  | **NFDHA** | NA/NA/NA | NA/NA/NA | NA/NA/NA | NA/NA/NA | ND/ND/ND | --/--/-- | NA/NA/NA/NA | NA/NA/NA/NA | NA/NA/NA/NA | NA/NA/NA/NA |
|  | **HFPODA** | ND (RL: 300)/ND (RL: 300)/ND (RL: 300) | --/--/-- | ND/ND/ND | --/--/-- | ND/ND/ND | --/--/-- | ND/ND/ND/ND | --/--/--/Q | ND/ND/ND/ND | --/--/--/Q |
|  | **DONA** | ND (RL: 200)/ND (RL: 200)/ND (RL: 200) | --/--/-- | ND/ND/ND | --/--/-- | ND/ND/ND | --/--/-- | ND/ND/ND/ND | --/--/--/Q | ND/ND/ND/ND | --/--/--/Q |
| **Perfluorinated carboxylic acids** | **PFPA** | NA/NA/NA | NA/NA/NA | NA/NA/NA | NA/NA/NA | NA/NA/NA | NA/NA/NA | NA/NA/NA/NA | NA/NA/NA/NA | NA/NA/NA/NA | NA/NA/NA/NA |
| **Perfluoroalkyl carboxylic acids** | **PFBA** | ND (RL: 500)/ND (RL: 500)/ND (RL: 500) | --/--/-- | ND/ND/ND | --/--/-- | CI/CI/CI | --/--/-- | 7/6.4/6.9/7.4 | 3.24/2.95/3.17/Q | ND/ND/ND/ND | --/--/--/Q |
|  | **PFPeA** | ND (RL: 200)/ND (RL: 200)/ND (RL: 200) | --/--/-- | ND/ND/ND | --/--/-- | ND/ND/ND | --/--/-- | 21/21/22/23 | 9.72/9.68/10.1/Q | <RL (J)/<RL (J)/<RL (J)/<RL (J) | --/--/--/-- |
|  | **PFHxA** | ND (RL: 200)/ND (RL: 200)/ND (RL: 200) | --/--/-- | ND/ND/<RL (J) | --/--/-- | 1.27/1.19/1.01 | 1.27E-06/1.19E-06/1.01E-06 | 29/25/26/29 | 13.42/11.52/11.94/Q | <RL (J)/<RL (J)/<RL (J)/J I | --/--/--/Q |
|  | **PFHpA** | ND (RL: 200)/ND (RL: 200)/ND (RL: 200) | --/--/-- | ND/ND/ND | --/--/-- | ND/ND/ND | --/--/-- | 8/7.5/7.7/6.9 | 3.7/3.46/3.53/Q | <RL (J)/<RL (J)/J I/<RL (J) | --/--/Q/-- |
|  | **PFOA** | ND (RL: 200)/<RL (RL: 200) (J)/<RL (RL: 200) (J) | --/--/-- | <RL (J)/<RL (J)/<RL (J) | --/--/-- | ND/<RL (J)/ND | --/--/-- | 16/15/15/15 | 7.4/6.91/6.89/Q | <RL (J)/<RL (J)/<RL (J)/<RL (J) | --/--/--/-- |
|  | **PFNA** | ND (RL: 200)/ND (RL: 200)/ND (RL: 200) | --/--/-- | ND/ND/ND | --/--/-- | ND/ND/ND | --/--/-- | <2.3 (J)/<2.1 (J)/<RL (J)/<RL (J) | Q/Q/--/-- | <RL (J)/ND/ND/ND | --/--/--/Q |
|  | **PFDA** | ND (RL: 200)/ND (RL: 200)/ND (RL: 200) | --/--/-- | <RL (J)/<RL (J)/22 | --/--/149.38 | ND/ND/ND | --/--/-- | 2.9/<2.1 (J)/2/<RL (J) | 1.34/Q/0.92/-- | ND/ND/ND/ND | --/--/--/Q |
|  | **PFUnA** | ND (RL: 200)/ND (RL: 200)/ND (RL: 200) | --/--/-- | ND/ND/ND | --/--/-- | ND/ND/ND | --/--/-- | ND/ND/ND/ND | --/--/--/Q | ND/ND/ND/ND | --/--/--/Q |
|  | **PFDoA** | ND (RL: 200)/ND (RL: 200)/ND (RL: 200) | --/--/-- | ND/ND/<RL (J) | --/--/-- | ND/ND/ND | --/--/-- | <2.3 (J)/ND/<RL (J)/ND | Q/--/--/Q | ND/ND/ND/ND | --/--/--/Q |
|  | **PFTriA** | ND (RL: 200)/ND (RL: 200)/ND (RL: 200) | --/--/-- | ND/ND/ND | --/--/-- | *-/*-/*- | --/--/-- | ND/ND/ND/ND | --/--/--/Q | ND/ND/ND/ND | --/--/--/Q |
|  | **PFTeA** | ND (RL: 200)/ND (RL: 200)/ND (RL: 200) | --/--/-- | ND/ND/ND | --/--/-- | ND/ND/ND | --/--/-- | ND/ND/ND/ND | --/--/--/Q | ND/ND/ND/ND | --/--/--/Q |
|  | **PFHxDA** | ND (RL: 300)/ND (RL: 300)/ND (RL: 300) | --/--/-- | ND/ND/ND | --/--/-- | ND/ND/ND | --/--/-- | ND/ND/ND/ND | --/--/--/Q | ND/ND/ND/ND | --/--/--/Q |
|  | **PFODA** | ND (RL: 300)/ND (RL: 300)/ND (RL: 300) | --/--/-- | ND/ND/ND | --/--/-- | *- *1/*- *1/*- *1 | --/--/-- | ND/ND/ND/ND | --/--/--/Q | ND/ND/ND/ND | --/--/--/Q |
| **Perfluoroalkyl sulfonic acids** | **PFEESA/PES** | NA/NA/NA | NA/NA/NA | NA/NA/NA | NA/NA/NA | ND/ND/ND | --/--/-- | NA/NA/NA/NA | NA/NA/NA/NA | NA/NA/NA/NA | NA/NA/NA/NA |
|  | **PFBS** | ND (RL: 200)/ND (RL: 200)/ND (RL: 200) | --/--/-- | ND/ND/ND | --/--/-- | ND/ND/ND | --/--/-- | 4.8/4.7/4/4.9 | 2.22/2.17/1.84/Q | <RL (J)/<RL (J)/<RL (J)/<RL (J) | --/--/--/-- |
|  | **PFPeS** | ND (RL: 200)/ND (RL: 200)/ND (RL: 200) | --/--/-- | ND/ND/ND | --/--/-- | ND/ND/ND | --/--/-- | ND/<2.1 (J)/<RL (J)/ND | --/Q/--/Q | ND/ND/ND/ND | --/--/--/Q |
|  | **PFHxS** | ND (RL: 200)/ND (RL: 200)/ND (RL: 200) | --/--/-- | ND/ND/ND | --/--/-- | ND/ND/ND | --/--/-- | 2.7/2.8/2.9/3.2 | 1.25/1.29/1.33/Q | <RL (J)/<RL (J)/<RL (J)/<RL (J) | --/--/--/-- |
|  | **PFHpS** | ND (RL: 200)/ND (RL: 200)/ND (RL: 200) | --/--/-- | ND/ND/ND | --/--/-- | ND/ND/ND | --/--/-- | ND/ND/ND/ND | --/--/--/Q | ND/ND/ND/ND | --/--/--/Q |
|  | **PFOS** | ND (RL: 200)/ND (RL: 200)/ND (RL: 200) | --/--/-- | 23/28/49 | 178.92/209.5/332.71 | ND/ND/ND | --/--/-- | 4.2/4.3/4.3/4.1 | 1.94/1.98/1.97/Q | <RL (J)/<RL (J)/<RL (J)/<RL (J) | --/--/--/-- |
|  | **PFNS** | ND (RL: 200)/ND (RL: 200)/ND (RL: 200) | --/--/-- | ND/ND/ND | --/--/-- | ND/ND/ND | --/--/-- | ND/ND/ND/ND | --/--/--/Q | ND/ND/ND/ND | --/--/--/Q |
|  | **PFDS** | ND (RL: 200)/ND (RL: 200)/ND (RL: 200) | --/--/-- | ND/ND/<RL (J) | --/--/-- | *-/*-/*- | --/--/-- | ND/ND/ND/ND | --/--/--/Q | ND/ND/ND/ND | --/--/--/Q |
|  | **PFDoS** | ND (RL: 300)/ND (RL: 300)/ND (RL: 300) | --/--/-- | ND/ND/<RL (J) | --/--/-- | *- *1/*- *1/*- *1 | --/--/-- | ND/ND/ND/ND | --/--/--/Q | ND/ND/ND/ND | --/--/--/Q |
| **Perfluorooctane sulfonamide ethanols** | **NEtFOSE** | ND (RL: 300)/ND (RL: 300)/ND (RL: 300) | --/--/-- | ND/ND/ND | --/--/-- | ND/ND/ND | --/--/-- | ND/ND/ND/ND | --/--/--/Q | ND/ND/ND/ND | --/--/--/Q |
|  | **NMeFOSA** | NA/NA/NA | NA/NA/NA | NA/NA/NA | NA/NA/NA | ND/ND/ND | --/--/-- | NA/NA/NA/NA | NA/NA/NA/NA | NA/NA/NA/NA | NA/NA/NA/NA |
|  | **NMeFOSE** | ND (RL: 300)/ND (RL: 300)/ND (RL: 300) | --/--/-- | ND/ND/ND | --/--/-- | NA/NA/NA | NA/NA/NA | ND/ND/ND/ND | --/--/--/Q | ND/ND/ND/ND | --/--/--/Q |
| **Perfluorooctane sulfonamides** | **NEtFOSA** | ND (RL: 500)/ND (RL: 500)/ND (RL: 500) | --/--/-- | ND/ND/ND | --/--/-- | ND/ND/ND | --/--/-- | ND/ND/ND/ND | --/--/--/Q | ND/ND/ND/ND | --/--/--/Q |
|  | **NMeFOSA** | ND (RL: 300)/ND (RL: 300)/ND (RL: 300) | --/--/-- | ND/ND/ND | --/--/-- | ND/ND/ND | --/--/-- | ND/ND/ND/ND | --/--/--/Q | <RL (J)/ND/ND/ND | --/--/--/Q |
|  | **FOSA** | ND (RL: 200)/ND (RL: 200)/ND (RL: 200) | --/--/-- | ND/ND/ND | --/--/-- | ND/ND/ND | --/--/-- | 4.6/<2.1 (J)/<RL (J)/<RL (J) | 2.13/Q/--/-- | 7.1/4.6/4.8/4.4 | 0.07/0.04/0.04/Q |
| **Perfluorooctane sulfonamidoacetic acids** | **NEtFOSAA** | ND (RL: 300)/ND (RL: 300)/ND (RL: 300) | --/--/-- | <RL (J)/<RL (J)/<RL (J) | --/--/-- | ND/ND/ND | --/--/-- | ND/ND/ND/ND | --/--/--/Q | ND/ND/ND/ND | --/--/--/Q |
|  | **NMeFOSAA** | ND (RL: 200)/ND (RL: 200)/ND (RL: 200) | --/--/-- | ND/<RL (J)/<RL (J) | --/--/-- | ND/ND/ND | --/--/-- | ND/ND/ND/ND | --/--/--/Q | ND/ND/ND/ND | --/--/--/Q |
|  | **Units** | **AOF, EOF : µg F/L, ng F/g** | **mg F/run** | **AOF, EOF : µg F/L, ng F/g** | **mg F/run** | **AOF, EOF : µg F/L, ng F/g** | **mg F/run** | **AOF, EOF : µg F/L, ng F/g** | **mg F/run** | **AOF, EOF : µg F/L, ng F/g** | **mg F/run** |
| **TOF** | **AOF** | 220 (RL: 20)/<RL/160 (RL: 20) | 63426.66/--/45295.33 | NA/NA/NA | NA/--/NA | NA/NA/NA | NA/NA/NA | <RL/<RL/<RL/<RL | --/--/--/-- | 3.2/2.6/<RL/2.2 | 29.8/24.11/--/-- |
|  | **EOF** | NA/NA/NA | NA/NA/NA | ND/ND/ND | --/--/-- | NA/NA/NA | NA/NA/NA | NA/NA/NA/NA | NA/NA/NA/NA | NA/NA/NA/NA | NA/NA/NA/NA |

| **Table** S-11**:** **Dryer System Intermediate Samples – Full Results** | | | | | | | | | | | |
| --- | --- | --- | --- | --- | --- | --- | --- | --- | --- | --- | --- |
|  |  | **Dewatered Solids** | | **RTO Inlet (OTM-45 Front Half Wash)** | | **RTO Inlet (OTM-45 Back Half Wash)** | | **RTO Inlet (OTM-45 Impingers)** | | **RTO Inlet (OTM-45 Breakthrough XAD Cartridge)** | |
|  |  | **Concentration** | **Emission** | **Concentration** | **Emission** | **Concentration** | **Emission** | **Concentration** | **Emission** | **Concentration** | **Emission** |
| **Units** |  | **ng/g** | **mg/run** | **ng/Sample** | **mg/run** | **ng/Sample** | **mg/run** | **ng/Sample** | **mg/run** | **ng/Sample** | **mg/run** |
| **PFAS Family** | **Acronym** | ***(R1/R2/R3/R3 Duplicate)*** | ***(R1/R2/R3/R3 Duplicate)*** | ***(R1/R2/R3/R4)*** | ***(R1/R2/R3/R4)*** | ***(R1/R2/R3/R4)*** | ***(R1/R2/R3/R4)*** | ***(R1/R2/R3/R4)*** | ***(R1/R2/R3/R4)*** | ***(R1/R2/R3/R4)*** | ***(R1/R2/R3/R4)*** |
| **Cyclic PFAS** | **PFECHS** | NA/NA/NA/NA | NA/NA/NA/NA | ND/ND/ND/ND | --/--/--/-- | ND/ND/ND/ND | --/--/--/-- | ND/ND/ND/ND | --/--/--/-- | ND/ND/ND/ND | --/--/--/-- |
| **Ether sulfonic acids** | **9Cl-PF3ONS** | ND/ND/ND/ND | --/--/--/-- | ND/ND/ND/ND | --/--/--/-- | ND/ND/ND/ND | --/--/--/-- | ND/ND/ND/ND | --/--/--/-- | ND/ND/ND/ND | --/--/--/-- |
|  | **11Cl-PF3OUdS** | ND/ND/ND/ND | --/--/--/-- | ND/ND/ND/ND | --/--/--/-- | *-/*-/*-/*- | --/--/--/-- | *- *1/*- *1/*- *1/*- *1 | --/--/--/-- | *-/*-/*-/*- | --/--/--/-- |
| **Fluorotelomer carboxylic acids** | **3:3 FTCA** | NA/NA/NA/NA | NA/NA/NA/NA | ND/ND/ND/ND | --/--/--/-- | ND/ND/ND/ND | --/--/--/-- | ND/ND/ND/ND | --/--/--/-- | ND/ND/ND/ND | --/--/--/-- |
|  | **5:3 FTCA** | NA/NA/NA/NA | NA/NA/NA/NA | 9.34/ND/ND/ND | 9.34E-06/--/--/-- | *+/*+/*+/*+ | Q/Q/--/-- | 162/221/ND/ND | 1.62E-04/2.21E-04/--/-- | J *+/J *+/J *+/*+ | Q/Q/Q/-- |
|  | **6:2 FTCA** | NA/NA/NA/NA | NA/NA/NA/NA | <RL (J)/ND/ND/ND | --/--/--/-- | 36.9/24.6/ND/ND | 3.69E-05/2.46E-05/--/-- | <RL (J)/ND/ND/ND | --/--/--/-- | 2.94/3.93/6.98/ND | 2.94E-06/3.93E-06/6.98E-06/-- |
|  | **7:3 FTCA** | NA/NA/NA/NA | NA/NA/NA/NA | <RL (J)/ND/ND/ND | --/--/--/-- | 5.08/ND/ND/ND | 5.08E-06/--/--/-- | 51.2/ND/ND/ND | 5.12E-05/--/--/-- | <RL (J)/ND/ND/ND | --/--/--/-- |
|  | **8:2 FTCA** | NA/NA/NA/NA | NA/NA/NA/NA | ND/ND/ND/ND | --/--/--/-- | 6.17/ND/ND/ND | 6.17E-06/--/--/-- | ND/ND/ND/ND | --/--/--/-- | ND/ND/ND/ND | --/--/--/-- |
|  | **10:2 FTCA** | NA/NA/NA/NA | NA/NA/NA/NA | ND/ND/ND/ND | --/--/--/-- | J I/ND/ND/ND | Q/--/--/-- | ND/ND/ND/ND | --/--/--/-- | ND/ND/ND/ND | --/--/--/-- |
| **Fluorotelomer sulfonic acids** | **4:2 FTS** | ND/ND/ND/ND | --/--/--/-- | ND/ND/ND/ND | --/--/--/-- | ND/ND/ND/ND | --/--/--/-- | ND/ND/ND/ND | --/--/--/-- | ND/ND/ND/ND | --/--/--/-- |
|  | **6:2 FTS** | ND/ND/ND/ND | --/--/--/-- | ND/ND/ND/ND | --/--/--/-- | ND/ND/ND/ND | --/--/--/-- | ND/ND/ND/ND | --/--/--/-- | ND/ND/ND/ND | --/--/--/-- |
|  | **8:2 FTS** | ND/ND/ND/<RL (J) | --/--/--/-- | <RL (J)/ND/ND/ND | --/--/--/-- | 6.26/<RL (J)/ND/ND | 6.26E-06/--/--/-- | ND/ND/ND/ND | --/--/--/-- | ND/ND/ND/ND | --/--/--/-- |
|  | **10:2 FTS** | <RL (J)/11/<RL (J)/11 | --/58.44/--/16.53 | <RL (J)/ND/ND/ND | --/--/--/-- | 2.62/ND/ND/ND | 2.62E-06/--/--/-- | ND/ND/ND/ND | --/--/--/-- | ND/ND/ND/ND | --/--/--/-- |
| **Fluorotelomer unsaturated carboxylic acids** | **6:2 FTUCA** | NA/NA/NA/NA | NA/NA/NA/NA | ND/ND/ND/ND | --/--/--/-- | 21.6/<RL (J)/ND/ND | 2.16E-05/--/--/-- | ND/ND/ND/ND | --/--/--/-- | 1.31/1.98/2.65/ND | 1.31E-06/1.98E-06/2.65E-06/-- |
|  | **8:2 FTUCA** | NA/NA/NA/NA | NA/NA/NA/NA | ND/ND/ND/ND | --/--/--/-- | 5.5/ND/ND/ND | 5.50E-06/--/--/-- | ND/ND/ND/ND | --/--/--/-- | ND/ND/ND/ND | --/--/--/-- |
| **Per- and Polyfluoroether carboxylic acids** | **PFMPA** | NA/NA/NA/NA | NA/NA/NA/NA | ND/ND/ND/ND | --/--/--/-- | ND/ND/ND/ND | --/--/--/-- | ND/ND/ND/ND | --/--/--/-- | ND/ND/ND/ND | --/--/--/-- |
|  | **PFMBA** | NA/NA/NA/NA | NA/NA/NA/NA | ND/ND/ND/ND | --/--/--/-- | ND/ND/ND/ND | --/--/--/-- | ND/ND/ND/ND | --/--/--/-- | ND/ND/ND/ND | --/--/--/-- |
|  | **NFDHA** | NA/NA/NA/NA | NA/NA/NA/NA | ND/ND/ND/ND | --/--/--/-- | ND/ND/ND/ND | --/--/--/-- | ND/ND/ND/ND | --/--/--/-- | ND/ND/ND/ND | --/--/--/-- |
|  | **HFPODA** | ND/ND/ND/ND | --/--/--/-- | I B/B/B/B | Q/Q/Q/Q | I B/B/B/B | Q/Q/Q/Q | B/B/B/B | Q/Q/Q/Q | ND/21.4/117/33.6 | --/2.14E-05/1.17E-04/3.36E-05 |
|  | **DONA** | ND/ND/ND/ND | --/--/--/-- | ND/ND/ND/ND | --/--/--/-- | ND/ND/ND/ND | --/--/--/-- | ND/ND/ND/ND | --/--/--/-- | ND/ND/ND/ND | --/--/--/-- |
| **Perfluorinated carboxylic acids** | **PFPA** | NA/NA/NA/NA | NA/NA/NA/NA | NA/NA/NA/NA | NA/NA/NA/NA | NA/NA/NA/NA | NA/NA/NA/NA | NA/NA/NA/NA | NA/NA/NA/NA | NA/NA/NA/NA | NA/NA/NA/NA |
| **Perfluoroalkyl carboxylic acids** | **PFBA** | ND/ND/ND/ND | --/--/--/-- | 4.05/ND/ND/ND | 4.05E-06/--/--/-- | 36.5/ND/ND/ND | 3.65E-05/--/--/-- | <RL (J)/ND/ND/ND | --/--/--/-- | CI/ND/ND/CI | --/--/--/-- |
|  | **PFPeA** | <RL (J)/ND/ND/ND | --/--/--/-- | 3.81/<RL (J)/ND/<RL (J) | 3.81E-06/--/--/-- | 30.1/37.6/ND/ND | 3.01E-05/3.76E-05/--/-- | <RL (J)/<RL (J)/<RL (J)/ND | --/--/--/-- | 1.64/2.38/2.76/ND | 1.64E-06/2.38E-06/2.76E-06/-- |
|  | **PFHxA** | 5.6/7.2/5.2/5.8 | 29.19/38.25/31.78/8.72 | 7.06/<RL (J)/ND/<RL (J) | 7.06E-06/--/--/-- | 305/366/345/ND | 3.05E-04/3.66E-04/3.45E-04/-- | <RL (J)/ND/ND/ND | --/--/--/-- | 11.3/18.3/23.6/ND | 1.13E-05/1.83E-05/2.36E-05/-- |
|  | **PFHpA** | ND/ND/ND/ND | --/--/--/-- | <RL (J)/ND/ND/ND | --/--/--/-- | 15.4/ND/ND/ND | 1.54E-05/--/--/-- | ND/ND/ND/ND | --/--/--/-- | ND/ND/ND/ND | --/--/--/-- |
|  | **PFOA** | 6.3/4.8/4/5.5 | 32.84/25.5/24.44/8.26 | 5.4/ND/ND/ND | 5.40E-06/--/--/-- | B/B/B/B | Q/Q/--/-- | ND/ND/ND/ND | --/--/--/-- | 1.05/1.57/1.21/1.2 | 1.05E-06/1.57E-06/1.21E-06/1.20E-06 |
|  | **PFNA** | <RL (J)/<RL (J)/<RL (J)/<RL (J) | --/--/--/-- | <RL (J)/ND/ND/ND | --/--/--/-- | 5.77/ND/ND/ND | 5.77E-06/--/--/-- | ND/ND/ND/ND | --/--/--/-- | ND/ND/ND/ND | --/--/--/-- |
|  | **PFDA** | 10/11/10/11 | 52.12/58.44/61.11/16.53 | <RL (J)/ND/ND/ND | --/--/--/-- | 36.2/31.5/ND/ND | 3.62E-05/3.15E-05/--/-- | ND/ND/ND/ND | --/--/--/-- | ND/ND/ND/ND | --/--/--/-- |
|  | **PFUnA** | <RL (J)/<RL (J)/<RL (J)/<RL (J) | --/--/--/-- | ND/ND/ND/ND | --/--/--/-- | <RL (J)/ND/ND/ND | --/--/--/-- | ND/ND/ND/ND | --/--/--/-- | ND/ND/ND/ND | --/--/--/-- |
|  | **PFDoA** | 3.7/4.4/4.3/4.1 | 19.28/23.37/26.28/6.16 | <RL (J)/ND/ND/ND | --/--/--/-- | 8.87/<RL (J)/ND/ND | 8.87E-06/--/--/-- | ND/ND/ND/ND | --/--/--/-- | ND/ND/ND/ND | --/--/--/-- |
|  | **PFTriA** | ND/ND/ND/ND | --/--/--/-- | ND/ND/ND/ND | --/--/--/-- | *-/*-/*-/*- | --/--/--/-- | ND/ND/ND/ND | --/--/--/-- | *-/*-/*-/*- | --/--/--/-- |
|  | **PFTeA** | <RL (J)/<RL (J)/<RL (J)/<RL (J) | --/--/--/-- | ND/ND/ND/ND | --/--/--/-- | ND/ND/ND/ND | --/--/--/-- | ND/ND/ND/ND | --/--/--/-- | ND/ND/ND/ND | --/--/--/-- |
|  | **PFHxDA** | ND/ND/ND/ND | --/--/--/-- | ND/ND/ND/ND | --/--/--/-- | ND/ND/ND/ND | --/--/--/-- | ND/ND/ND/ND | --/--/--/-- | ND/ND/ND/ND | --/--/--/-- |
|  | **PFODA** | ND/ND/ND/ND | --/--/--/-- | ND/ND/ND/ND | --/--/--/-- | *- *1/*- *1/*- *1/*- *1 | --/--/--/-- | ND/ND/ND/ND | --/--/--/-- | *- *1/*- *1/*- *1/*- *1 | --/--/--/-- |
| **Perfluoroalkyl sulfonic acids** | **PFEESA/PES** | NA/NA/NA/NA | NA/NA/NA/NA | ND/ND/ND/ND | --/--/--/-- | ND/ND/ND/ND | --/--/--/-- | ND/ND/ND/ND | --/--/--/-- | ND/ND/ND/ND | --/--/--/-- |
|  | **PFBS** | ND/ND/ND/ND | --/--/--/-- | ND/ND/ND/ND | --/--/--/-- | ND/ND/ND/ND | --/--/--/-- | ND/ND/ND/ND | --/--/--/-- | ND/ND/ND/ND | --/--/--/-- |
|  | **PFPeS** | ND/ND/ND/ND | --/--/--/-- | ND/ND/ND/ND | --/--/--/-- | ND/ND/ND/ND | --/--/--/-- | ND/ND/ND/ND | --/--/--/-- | J I/ND/ND/ND | Q/--/--/-- |
|  | **PFHxS** | ND/ND/ND/ND | --/--/--/-- | ND/ND/ND/ND | --/--/--/-- | ND/ND/ND/ND | --/--/--/-- | ND/ND/ND/ND | --/--/--/-- | ND/ND/ND/ND | --/--/--/-- |
|  | **PFHpS** | J I/ND/ND/ND | Q/--/--/-- | ND/ND/ND/ND | --/--/--/-- | ND/ND/ND/ND | --/--/--/-- | ND/ND/ND/ND | --/--/--/-- | ND/ND/ND/ND | --/--/--/-- |
|  | **PFOS** | 21/25/25/23 | 109.45/132.81/152.77/34.56 | ND/ND/ND/ND | --/--/--/-- | I/ND/ND/ND | Q/--/--/-- | ND/ND/ND/ND | --/--/--/-- | ND/ND/ND/ND | --/--/--/-- |
|  | **PFNS** | ND/ND/ND/ND | --/--/--/-- | ND/ND/ND/ND | --/--/--/-- | ND/ND/ND/ND | --/--/--/-- | ND/ND/ND/ND | --/--/--/-- | ND/ND/ND/ND | --/--/--/-- |
|  | **PFDS** | <RL (J)/3.7/3.3/<RL (J) | --/19.66/20.17/-- | ND/ND/ND/ND | --/--/--/-- | *-/*-/*-/*- | --/--/--/-- | ND/ND/ND/ND | --/--/--/-- | *-/*-/*-/*- | --/--/--/-- |
|  | **PFDoS** | ND/<RL (J)/<RL (J)/<RL (J) | --/--/--/-- | J I/ND/ND/ND | Q/--/--/-- | *- *1/*- *1/*- *1/*- *1 | --/--/--/-- | *-/*-/*-/*- | --/--/--/-- | *- *1/*- *1/*- *1/*- *1 | --/--/--/-- |
| **Perfluorooctane sulfonamide ethanols** | **NEtFOSE** | ND/ND/ND/ND | --/--/--/-- | ND/ND/<RL (J)/ND | --/--/--/-- | 15.5/ND*/ND/ND | 1.55E-05/Q/--/-- | ND/ND/ND/ND | --/--/--/-- | <RL (J)/ND/ND/ND | --/--/--/-- |
|  | **NMeFOSA** | NA/NA/NA/NA | NA/NA/NA/NA | ND/ND/ND/ND | --/--/--/-- | ND/ND*/ND/ND | --/Q/--/-- | ND/ND/ND/ND | --/--/--/-- | ND/ND/ND/ND | --/--/--/-- |
|  | **NMeFOSE** | ND/<RL (J)/<RL (J)/ND | --/--/--/-- | NA/NA/NA/NA | NA/NA/NA/NA | NA/NA/NA/NA | NA/NA/NA/NA | NA/NA/NA/NA | NA/NA/NA/NA | NA/NA/NA/NA | NA/NA/NA/NA |
| **Perfluorooctane sulfonamides** | **NEtFOSA** | ND/ND/ND/ND | --/--/--/-- | ND/ND/ND/ND | --/--/--/-- | ND/ND/ND/ND | --/--/--/-- | ND/ND/ND/ND | --/--/--/-- | ND/ND/ND/ND | --/--/--/-- |
|  | **NMeFOSA** | ND/ND/ND/ND | --/--/--/-- | ND/ND/ND/ND | --/--/--/-- | ND/ND/ND/ND | --/--/--/-- | ND/ND/ND/ND | --/--/--/-- | ND/ND/ND/ND | --/--/--/-- |
|  | **FOSA** | <RL (J)/<RL (J)/<RL (J)/<RL (J) | --/--/--/-- | ND/ND/ND/ND | --/--/--/-- | <RL (J)/ND/ND/ND | --/--/--/-- | ND/ND/ND/ND | --/--/--/-- | ND/ND/ND/ND | --/--/--/-- |
| **Perfluorooctane sulfonamidoacetic acids** | **NEtFOSAA** | 15/16/16/16 | 78.18/85/97.77/24.04 | <RL (J)/ND/ND/ND | --/--/--/-- | <RL (J)/ND/ND/ND | --/--/--/-- | ND/ND/ND/ND | --/--/--/-- | ND/ND/ND/ND | --/--/--/-- |
|  | **NMeFOSAA** | 12/12/13/12 | 62.54/63.75/79.44/18.03 | <RL (J)/ND/ND/ND | --/--/--/-- | ND/ND/ND/ND | --/--/--/-- | ND/ND/ND/ND | --/--/--/-- | ND/ND/ND/ND | --/--/--/-- |
|  | **Units** | **AOF, EOF : µg F/L, ng F/g** | **mg F/run** | **AOF, EOF : µg F/L, ng F/g** | **mg F/run** | **AOF, EOF : µg F/L, ng F/g** | **mg F/run** | **AOF, EOF : µg F/L, ng F/g** | **mg F/run** | **AOF, EOF : µg F/L, ng F/g** | **mg F/run** |
| **TOF** | **AOF** | NA/NA/NA/NA | NA/NA/NA/NA | NA/NA/NA/NA | NA/NA/NA/NA | NA/NA/NA/NA | NA/NA/NA/NA | NA/NA/NA/NA | NA/NA/NA/NA | NA/NA/NA/NA | NA/NA/NA/NA |
|  | **EOF** | <RL/<RL/<RL/<RL | --/--/--/-- | NA/NA/NA/NA | NA/NA/NA/NA | NA/NA/NA/NA | NA/NA/NA/NA | NA/NA/NA/NA | NA/NA/NA/NA | NA/NA/NA/NA | NA/NA/NA/NA |

| **Table** S-12**:** **Dryer System Output Samples – Full Results** | | | | | | | | | | | | | | | | | | | |
| --- | --- | --- | --- | --- | --- | --- | --- | --- | --- | --- | --- | --- | --- | --- | --- | --- | --- | --- | --- |
|  |  | **Centrate (Liquid Fraction)** | | **Centrate (Solid Fraction** | | **Dried Solids** | | **Condenser Process Drain** | | **Scrubber Process Drain** | | **RTO Outlet (OTM-45 Front Half Wash)** | | **RTO Outlet (OTM-45 Back Half Wash)** | | **RTO Outlet (OTM-45 Impingers)** | | **RTO Outlet (OTM-45 Breakthrough XAD Cartridge)** | |
|  |  | **Concentration** | **Emission** | **Concentration** | **Emission** | **Concentration** | **Emission** | **Concentration** | **Emission** | **Concentration** | **Emission** | **Concentration** | **Emission** | **Concentration** | **Emission** | **Concentration** | **Emission** | **Concentration** | **Emission** |
| **Units** |  | **ng/L** | **mg/run** | **ng/g** | **mg/run** | **ng/g** | **mg/run** | **ng/L** | **mg/run** | **ng/L** | **mg/run** | **ng/Sample** | **mg/run** | **ng/Sample** | **mg/run** | **ng/Sample** | **mg/run** | **ng/Sample** | **mg/run** |
| **PFAS Family** | **Acronym** | ***(R1/R2/R3)*** | ***(R1/R2/R3)*** | ***(R1/R2/R3)*** | ***(R1/R2/R3)*** | ***(R1/R2/R3)*** | ***(R1/R2/R3)*** | ***(R1/R2/R3/R4)*** | ***(R1/R2/R3/R4)*** | ***(R1/R2/R3/R4)*** | ***(R1/R2/R3/R4)*** | ***(R1/R2/R3)*** | ***(R1/R2/R3)*** | ***(R1/R2/R3)*** | ***(R1/R2/R3)*** | ***(R1/R2/R3)*** | ***(R1/R2/R3)*** | ***(R1/R2/R3)*** | ***(R1/R2/R3)*** |
| **Cyclic PFAS** | **PFECHS** | NA/NA/NA | NA/NA/NA | NA/NA/NA | NA/NA/NA | NA/NA/NA | NA/NA/NA | NA/NA/NA/NA | NA/NA/NA/NA | NA/NA/NA/NA | NA/NA/NA/NA | ND/ND/ND | --/--/-- | ND/ND/ND | --/--/-- | ND/ND/ND | --/--/-- | ND/ND/ND | --/--/-- |
| **Ether sulfonic acids** | **9Cl-PF3ONS** | ND (RL: 20)/ND (RL: 20)/ND (RL: 20) | --/--/-- | ND/ND/ND | --/--/-- | ND/ND/ND | --/--/-- | ND (RL: 19)/ND/ND/ND | --/--/--/Q | ND/ND/ND/ND | --/--/--/Q | ND/ND/ND | --/--/-- | ND/ND/ND | --/--/-- | ND/ND/ND | --/--/-- | ND/ND/ND | --/--/-- |
|  | **11Cl-PF3OUdS** | ND (RL: 20)/ND (RL: 20)/ND (RL: 20) | --/--/-- | ND/ND/ND | --/--/-- | ND/ND/ND | --/--/-- | ND (RL: 19)/ND/ND/ND | --/--/--/Q | ND/ND/ND/ND | --/--/--/Q | ND/ND/ND | --/--/-- | *- *1/*- *1/*- *1 | --/--/-- | *- *1/*- *1/*- *1 | --/--/-- | *- *1/*- *1/*- *1 | --/--/-- |
| **Fluorotelomer carboxylic acids** | **3:3 FTCA** | NA/NA/NA | NA/NA/NA | NA/NA/NA | NA/NA/NA | NA/NA/NA | NA/NA/NA | NA/NA/NA/NA | NA/NA/NA/NA | NA/NA/NA/NA | NA/NA/NA/NA | ND/ND/ND | --/--/-- | ND/ND/ND | --/--/-- | ND/ND/ND | --/--/-- | ND/ND/ND | --/--/-- |
|  | **5:3 FTCA** | NA/NA/NA | NA/NA/NA | NA/NA/NA | NA/NA/NA | NA/NA/NA | NA/NA/NA | NA/NA/NA/NA | NA/NA/NA/NA | NA/NA/NA/NA | NA/NA/NA/NA | ND/ND/ND | --/--/-- | *+/*+/J *+ | --/--/Q | ND/ND/ND | --/--/-- | *+/*+/*+ | --/--/-- |
|  | **6:2 FTCA** | NA/NA/NA | NA/NA/NA | NA/NA/NA | NA/NA/NA | NA/NA/NA | NA/NA/NA | NA/NA/NA/NA | NA/NA/NA/NA | NA/NA/NA/NA | NA/NA/NA/NA | ND/ND/ND | --/--/-- | ND/ND/ND | --/--/-- | ND/ND/ND | --/--/-- | ND/ND/ND | --/--/-- |
|  | **7:3 FTCA** | NA/NA/NA | NA/NA/NA | NA/NA/NA | NA/NA/NA | NA/NA/NA | NA/NA/NA | NA/NA/NA/NA | NA/NA/NA/NA | NA/NA/NA/NA | NA/NA/NA/NA | ND/ND/ND | --/--/-- | *+/*+/*+ | --/--/-- | ND/ND/ND | --/--/-- | *+/*+/*+ | --/--/-- |
|  | **8:2 FTCA** | NA/NA/NA | NA/NA/NA | NA/NA/NA | NA/NA/NA | NA/NA/NA | NA/NA/NA | NA/NA/NA/NA | NA/NA/NA/NA | NA/NA/NA/NA | NA/NA/NA/NA | ND/ND/ND | --/--/-- | ND/ND/ND | --/--/-- | ND/ND/ND | --/--/-- | ND/ND/ND | --/--/-- |
|  | **10:2 FTCA** | NA/NA/NA | NA/NA/NA | NA/NA/NA | NA/NA/NA | NA/NA/NA | NA/NA/NA | NA/NA/NA/NA | NA/NA/NA/NA | NA/NA/NA/NA | NA/NA/NA/NA | ND/ND/ND | --/--/-- | ND/ND/ND | --/--/-- | ND/ND/ND | --/--/-- | ND/ND/ND | --/--/-- |
| **Fluorotelomer sulfonic acids** | **4:2 FTS** | ND (RL: 20)/ND (RL: 20)/ND (RL: 20) | --/--/-- | ND/ND/ND | --/--/-- | ND/ND/ND | --/--/-- | ND (RL: 19)/ND/ND/ND | --/--/--/Q | ND/ND/ND/ND | --/--/--/Q | ND/ND/ND | --/--/-- | ND/ND/ND | --/--/-- | ND/ND/ND | --/--/-- | ND/ND/ND | --/--/-- |
|  | **6:2 FTS** | ND (RL: 50)/ND (RL: 50)/ND (RL: 49) | --/--/-- | ND/ND/ND | --/--/-- | ND/ND/ND | --/--/-- | ND (RL: 48)/ND/ND/ND | --/--/--/Q | ND/ND/ND/ND | --/--/--/Q | ND/ND/ND | --/--/-- | ND/ND/ND | --/--/-- | ND/ND/ND | --/--/-- | ND/ND/ND | --/--/-- |
|  | **8:2 FTS** | ND (RL: 30)/ND (RL: 30)/ND (RL: 30) | --/--/-- | ND/ND/ND | --/--/-- | <RL (J)/<RL (J)/<RL (J) | --/--/-- | ND (RL: 29)/ND/ND/ND | --/--/--/Q | ND/ND/ND/ND | --/--/--/Q | ND/<RL (J)/<RL (J) | --/--/-- | ND/ND/ND | --/--/-- | ND/ND/ND | --/--/-- | ND/ND/ND | --/--/-- |
|  | **10:2 FTS** | ND (RL: 50)/ND (RL: 50)/ND (RL: 49) | --/--/-- | <RL (J)/<RL (J)/<RL (J) | --/--/-- | 2.4/2.8/2.7 | 12.17/14.77/16.48 | ND (RL: 48)/ND/ND/ND | --/--/--/Q | ND/ND/ND/ND | --/--/--/Q | ND/ND/ND | --/--/-- | ND/ND/ND | --/--/-- | ND/ND/ND | --/--/-- | ND/ND/ND | --/--/-- |
| **Fluorotelomer unsaturated carboxylic acids** | **6:2 FTUCA** | NA/NA/NA | NA/NA/NA | NA/NA/NA | NA/NA/NA | NA/NA/NA | NA/NA/NA | NA/NA/NA/NA | NA/NA/NA/NA | NA/NA/NA/NA | NA/NA/NA/NA | ND/ND/ND | --/--/-- | ND/ND/ND | --/--/-- | ND/ND/ND | --/--/-- | ND/ND/ND | --/--/-- |
|  | **8:2 FTUCA** | NA/NA/NA | NA/NA/NA | NA/NA/NA | NA/NA/NA | NA/NA/NA | NA/NA/NA | NA/NA/NA/NA | NA/NA/NA/NA | NA/NA/NA/NA | NA/NA/NA/NA | ND/ND/ND | --/--/-- | ND/ND/ND | --/--/-- | ND/ND/ND | --/--/-- | ND/ND/ND | --/--/-- |
| **Per- and Polyfluoroether carboxylic acids** | **PFMPA** | NA/NA/NA | NA/NA/NA | NA/NA/NA | NA/NA/NA | NA/NA/NA | NA/NA/NA | NA/NA/NA/NA | NA/NA/NA/NA | NA/NA/NA/NA | NA/NA/NA/NA | ND/ND/ND | --/--/-- | ND/ND/ND | --/--/-- | ND/ND/ND | --/--/-- | ND/ND/ND | --/--/-- |
|  | **PFMBA** | NA/NA/NA | NA/NA/NA | NA/NA/NA | NA/NA/NA | NA/NA/NA | NA/NA/NA | NA/NA/NA/NA | NA/NA/NA/NA | NA/NA/NA/NA | NA/NA/NA/NA | ND/ND/ND | --/--/-- | ND/ND/ND | --/--/-- | ND/ND/ND | --/--/-- | ND/ND/ND | --/--/-- |
|  | **NFDHA** | NA/NA/NA | NA/NA/NA | NA/NA/NA | NA/NA/NA | NA/NA/NA | NA/NA/NA | NA/NA/NA/NA | NA/NA/NA/NA | NA/NA/NA/NA | NA/NA/NA/NA | ND/ND/ND | --/--/-- | ND/ND/ND | --/--/-- | ND/ND/ND | --/--/-- | ND/ND/ND | --/--/-- |
|  | **HFPODA** | ND (RL: 30)/ND (RL: 30)/ND (RL: 30) | --/--/-- | ND/ND/ND | --/--/-- | ND/ND/ND | --/--/-- | ND (RL: 29)/ND/ND/ND | --/--/--/Q | ND/ND/ND/ND | --/--/--/Q | B/B/B | Q/Q/Q | B/B/B | Q/Q/Q | B/B/B | Q/Q/Q | 43.8/ND/42.1 | 4.38E-05/--/4.21E-05 |
|  | **DONA** | ND (RL: 20)/ND (RL: 20)/ND (RL: 20) | --/--/-- | ND/ND/ND | --/--/-- | ND/ND/ND | --/--/-- | ND (RL: 19)/ND/ND/ND | --/--/--/Q | ND/ND/ND/ND | --/--/--/Q | ND/ND/ND | --/--/-- | ND/ND/ND | --/--/-- | ND/ND/ND | --/--/-- | ND/ND/ND | --/--/-- |
| **Perfluorinated carboxylic acids** | **PFPA** | NA/NA/NA | NA/NA/NA | NA/NA/NA | NA/NA/NA | NA/NA/NA | NA/NA/NA | NA/NA/NA/NA | NA/NA/NA/NA | NA/NA/NA/NA | NA/NA/NA/NA | NA/NA/NA | NA/NA/NA | NA/NA/NA | NA/NA/NA | NA/NA/NA | NA/NA/NA | NA/NA/NA | NA/NA/NA |
| **Perfluoroalkyl carboxylic acids** | **PFBA** | ND (RL: 50)/ND (RL: 50)/ND (RL: 49) | --/--/-- | ND/ND/ND | --/--/-- | ND/ND/ND | --/--/-- | ND (RL: 48)/8.1/9.1/7.5 | --/3.9/4.36/Q | 7/7/7.4/7.3 | 0.07/0.06/0.07/Q | ND/ND/ND | --/--/-- | ND/ND/ND | --/--/-- | ND/1.63/ND | --/1.63E-06/-- | CI/CI/CI | --/--/-- |
|  | **PFPeA** | 32 (RL: 20)/32 (RL: 20)/31 (RL: 20) | 8.51/8.57/7.98 | ND/ND/ND | --/--/-- | <RL (J)/<RL (J)/<RL (J) | --/--/-- | 24 (RL: 19)/21/21/22 | 11.6/10.11/10.07/Q | 19/19/20/22 | 0.18/0.18/0.18/Q | ND/<RL (J)/ND | --/--/-- | ND/ND/ND | --/--/-- | ND/0.739/ND | --/7.39E-07/-- | ND/ND/ND | --/--/-- |
|  | **PFHxA** | 61 (RL: 20)/67 (RL: 20)/64 (RL: 20) | 16.22/17.94/16.48 | <RL (J)/<RL (J)/<RL (J) | --/--/-- | 0.75/0.86/0.81 | 3.8/4.54/4.94 | 29 (RL: 19)/26/29/33 | 14.02/12.51/13.9/Q | 25/25/25/28 | 0.23/0.23/0.23/Q | ND/<RL (J)/ND | --/--/-- | ND/ND/ND | --/--/-- | ND/ND/ND | --/--/-- | ND/<RL (J)/ND | --/--/-- |
|  | **PFHpA** | 20 (RL: 20)/20 (RL: 20)/20 (RL: 20) | 5.32/5.36/5.15 | ND/ND/ND | --/--/-- | ND/ND/ND | --/--/-- | <RL (RL: 19) (J)/8.9/9/9.5 | --/4.28/4.31/Q | 6.1/7.3/7.8/7.1 | 0.06/0.07/0.07/Q | ND/ND/ND | --/--/-- | ND/ND/ND | --/--/-- | ND/<RL (J)/ND | --/--/-- | ND/ND/ND | --/--/-- |
|  | **PFOA** | 71 (RL: 20)/68 (RL: 20)/69 (RL: 20) | 18.88/18.21/17.76 | <RL (J)/<RL (J)/<RL (J) | --/--/-- | 1.3/1.3/1.3 | 6.59/6.86/7.93 | <RL (RL: 19) (J)/19/19/23 | --/9.15/9.11/Q | 15/14/15/14 | 0.14/0.13/0.14/Q | ND/ND/ND | --/--/-- | ND/ND/<RL (J) | --/--/-- | ND/1.05/ND | --/1.05E-06/-- | <RL (J)/ND/1.41 | --/--/1.41E-06 |
|  | **PFNA** | <RL (RL: 20) (J)/<RL (RL: 20) (J)/<RL (RL: 20) (J) | --/--/-- | ND/ND/ND | --/--/-- | 0.81/0.81/0.81 | 4.11/4.27/4.94 | ND (RL: 19)/2/1.9/2.2 | --/0.96/0.91/Q | <RL (J)/<RL (J)/<RL (J)/<RL (J) | --/--/--/-- | ND/<RL (J)/ND | --/--/-- | ND/ND/ND | --/--/-- | ND/<RL (J)/ND | --/--/-- | ND/ND/ND | --/--/-- |
|  | **PFDA** | <RL (RL: 20) (J)/<RL (RL: 20) (J)/<RL (RL: 20) (J) | --/--/-- | 13/10/14 | 33.17/21.94/9.72 | 4.6/4.5/4.3 | 23.33/23.75/26.24 | ND (RL: 19)/2.6/2.4/2.5 | --/1.25/1.15/Q | <RL (J)/<RL (J)/1.8/<RL (J) | --/--/0.02/-- | ND/ND/ND | --/--/-- | ND/ND/ND | --/--/-- | ND/ND/ND | --/--/-- | ND/ND/ND | --/--/-- |
|  | **PFUnA** | ND (RL: 20)/ND (RL: 20)/ND (RL: 20) | --/--/-- | ND/ND/ND | --/--/-- | 1/1.2/1 | 5.07/6.33/6.1 | ND (RL: 19)/ND/ND/ND | --/--/--/Q | ND/ND/ND/ND | --/--/--/Q | ND/ND/ND | --/--/-- | J I/ND/ND | Q/--/-- | ND/ND/ND | --/--/-- | ND/ND/ND | --/--/-- |
|  | **PFDoA** | ND (RL: 20)/ND (RL: 20)/ND (RL: 20) | --/--/-- | <RL (J)/<RL (J)/<RL (J) | --/--/-- | 1.6/1.9/1.8 | 8.11/10.03/10.98 | ND (RL: 19)/ND/ND/ND | --/--/--/Q | <RL (J)/ND/ND/ND | --/--/--/Q | ND/ND/ND | --/--/-- | J I/ND/J I | Q/--/Q | ND/ND/ND | --/--/-- | ND/ND/ND | --/--/-- |
|  | **PFTriA** | ND (RL: 20)/ND (RL: 20)/ND (RL: 20) | --/--/-- | ND/ND/ND | --/--/-- | ND/ND/ND | --/--/-- | ND (RL: 19)/ND/ND/ND | --/--/--/Q | ND/ND/ND/ND | --/--/--/Q | ND/ND/ND | --/--/-- | ND/ND/ND | --/--/-- | ND/ND/ND | --/--/-- | ND/ND/ND | --/--/-- |
|  | **PFTeA** | ND (RL: 20)/ND (RL: 20)/ND (RL: 20) | --/--/-- | ND/ND/ND | --/--/-- | <RL (J)/<RL (J)/<RL (J) | --/--/-- | ND (RL: 19)/ND/ND/ND | --/--/--/Q | ND/ND/ND/ND | --/--/--/Q | ND/ND/ND | --/--/-- | ND/ND/ND | --/--/-- | ND/ND/ND | --/--/-- | ND/ND/ND | --/--/-- |
|  | **PFHxDA** | ND (RL: 30)/ND (RL: 30)/ND (RL: 30) | --/--/-- | ND/ND/ND | --/--/-- | <RL (J)/<RL (J)/<RL (J) | --/--/-- | ND (RL: 29)/ND/ND/ND | --/--/--/Q | ND/ND/ND/ND | --/--/--/Q | ND/ND/ND | --/--/-- | ND/ND/ND | --/--/-- | ND/ND/ND | --/--/-- | ND/ND/ND | --/--/-- |
|  | **PFODA** | ND (RL: 30)/ND (RL: 30)/ND (RL: 30) | --/--/-- | ND/ND/ND | --/--/-- | ND/ND/ND | --/--/-- | ND (RL: 29)/ND/ND/ND | --/--/--/Q | ND/ND/ND/ND | --/--/--/Q | ND/ND/ND | --/--/-- | *- *1/*- *1/*- *1 | --/--/-- | ND/ND/ND | --/--/-- | *- *1/*- *1/*- *1 | --/--/-- |
| **Perfluoroalkyl sulfonic acids** | **PFEESA/PES** | NA/NA/NA | NA/NA/NA | NA/NA/NA | NA/NA/NA | NA/NA/NA | NA/NA/NA | NA/NA/NA/NA | NA/NA/NA/NA | NA/NA/NA/NA | NA/NA/NA/NA | ND/ND/ND | --/--/-- | ND/ND/ND | --/--/-- | ND/ND/ND | --/--/-- | ND/ND/ND | --/--/-- |
|  | **PFBS** | <RL (RL: 20) (J)/<RL (RL: 20) (J)/<RL (RL: 20) (J) | --/--/-- | ND/ND/ND | --/--/-- | ND/ND/ND | --/--/-- | <RL (RL: 19) (J)/4/3.7/4.7 | --/1.93/1.77/Q | 5.4/4.4/4.3/4.8 | 0.05/0.04/0.04/Q | ND/ND/ND | --/--/-- | ND/ND/ND | --/--/-- | ND/ND/ND | --/--/-- | ND/ND/ND | --/--/-- |
|  | **PFPeS** | ND (RL: 20)/ND (RL: 20)/ND (RL: 20) | --/--/-- | ND/ND/ND | --/--/-- | ND/ND/ND | --/--/-- | ND (RL: 19)/<RL (J)/<RL (J)/J I | --/--/--/Q | <RL (J)/ND/<RL (J)/<RL (J) | --/--/--/-- | ND/ND/ND | --/--/-- | ND/ND/ND | --/--/-- | ND/ND/ND | --/--/-- | ND/ND/ND | --/--/-- |
|  | **PFHxS** | <RL (RL: 20) (J)/<RL (RL: 20) (J)/<RL (RL: 20) (J) | --/--/-- | ND/ND/ND | --/--/-- | ND/ND/ND | --/--/-- | ND (RL: 19)/3/3/4.1 | --/1.44/1.44/Q | 2.7/2.3/2.5/2.4 | 0.03/0.02/0.02/Q | ND/ND/ND | --/--/-- | ND/ND/ND | --/--/-- | ND/ND/ND | --/--/-- | ND/ND/ND | --/--/-- |
|  | **PFHpS** | ND (RL: 20)/ND (RL: 20)/ND (RL: 20) | --/--/-- | ND/ND/ND | --/--/-- | J I/ND/J I | Q/--/Q | ND (RL: 19)/ND/ND/ND | --/--/--/Q | ND/ND/ND/ND | --/--/--/Q | ND/ND/ND | --/--/-- | ND/ND/ND | --/--/-- | ND/ND/ND | --/--/-- | ND/ND/ND | --/--/-- |
|  | **PFOS** | 22 (RL: 20)/25 (RL: 20)/22 (RL: 20) | 5.85/6.69/5.66 | 28/22/32 | 71.44/48.28/22.23 | 9/10/9.9 | 45.64/52.77/60.42 | ND (RL: 19)/7.2/7.1/7.4 | --/3.47/3.4/Q | 5.4/4.6/5.1/3.9 | 0.05/0.04/0.05/Q | ND/ND/ND | --/--/-- | ND/ND/ND | --/--/-- | ND/J I/ND | --/Q/-- | ND/ND/ND | --/--/-- |
|  | **PFNS** | ND (RL: 20)/ND (RL: 20)/ND (RL: 20) | --/--/-- | ND/ND/ND | --/--/-- | ND/ND/ND | --/--/-- | ND (RL: 19)/ND/ND/ND | --/--/--/Q | ND/ND/ND/ND | --/--/--/Q | ND/ND/ND | --/--/-- | ND/ND/ND | --/--/-- | ND/ND/ND | --/--/-- | ND/ND/ND | --/--/-- |
|  | **PFDS** | ND (RL: 20)/ND (RL: 20)/ND (RL: 20) | --/--/-- | ND/<RL (J)/J I | --/--/Q | 1.2/1.3/1.1 | 6.09/6.86/6.71 | ND (RL: 19)/ND/ND/ND | --/--/--/Q | ND/ND/ND/ND | --/--/--/Q | ND/ND/ND | --/--/-- | ND/ND/ND | --/--/-- | ND/ND/ND | --/--/-- | ND/ND/ND | --/--/-- |
|  | **PFDoS** | ND (RL: 30)/ND (RL: 30)/ND (RL: 30) | --/--/-- | <RL (J)/<RL (J)/<RL (J) | --/--/-- | <RL (J)/<RL (J)/<RL (J) | --/--/-- | ND (RL: 29)/ND/ND/ND | --/--/--/Q | ND/ND/ND/ND | --/--/--/Q | ND/ND/ND | --/--/-- | *- *1/*- *1/*- *1 | --/--/-- | *-/*-/*- | --/--/-- | *- *1/*- *1/*- *1 | --/--/-- |
| **Perfluorooctane sulfonamide ethanols** | **NEtFOSE** | ND (RL: 30)/ND (RL: 30)/ND (RL: 30) | --/--/-- | ND/ND/ND | --/--/-- | ND/<RL (J)/<RL (J) | --/--/-- | ND (RL: 29)/<RL (J)/<RL (J)/ND | --/--/--/Q | <RL (J)/<RL (J)/<RL (J)/ND | --/--/--/Q | ND/<RL (J)/<RL (J) | --/--/-- | ND/ND/ND | --/--/-- | ND/ND/ND | --/--/-- | ND/ND/ND | --/--/-- |
|  | **NMeFOSA** | NA/NA/NA | NA/NA/NA | NA/NA/NA | NA/NA/NA | NA/NA/NA | NA/NA/NA | NA/NA/NA/NA | NA/NA/NA/NA | NA/NA/NA/NA | NA/NA/NA/NA | ND/ND/ND | --/--/-- | ND/ND/ND | --/--/-- | ND/ND/ND | --/--/-- | ND/ND/ND | --/--/-- |
|  | **NMeFOSE** | ND (RL: 30)/ND (RL: 30)/ND (RL: 30) | --/--/-- | ND/ND/ND | --/--/-- | ND/<RL (J)/ND | --/--/-- | ND (RL: 29)/<RL (J)/<RL (J)/ND | --/--/--/Q | 4.2/3.9/3.9/ND | 0.04/0.04/0.04/Q | NA/NA/NA | NA/NA/NA | NA/NA/NA | NA/NA/NA | NA/NA/NA | NA/NA/NA | NA/NA/NA | NA/NA/NA |
| **Perfluorooctane sulfonamides** | **NEtFOSA** | ND (RL: 50)/ND (RL: 50)/ND (RL: 49) | --/--/-- | ND/ND/ND | --/--/-- | ND/ND/ND | --/--/-- | ND (RL: 48)/ND/ND/ND | --/--/--/Q | ND/ND/ND/ND | --/--/--/Q | ND/ND/ND | --/--/-- | ND/ND/ND | --/--/-- | ND/ND/ND | --/--/-- | ND/ND/ND | --/--/-- |
|  | **NMeFOSA** | ND (RL: 30)/ND (RL: 30)/ND (RL: 30) | --/--/-- | ND/ND/ND | --/--/-- | ND/ND/ND | --/--/-- | ND (RL: 29)/ND/ND/ND | --/--/--/Q | ND/ND/ND/ND | --/--/--/Q | ND/ND/ND | --/--/-- | ND/ND/ND | --/--/-- | ND/ND/ND | --/--/-- | ND/ND/ND | --/--/-- |
|  | **FOSA** | ND (RL: 20)/ND (RL: 20)/ND (RL: 20) | --/--/-- | ND/ND/ND | --/--/-- | <RL (J)/<RL (J)/<RL (J) | --/--/-- | ND (RL: 19)/<RL (J)/<RL (J)/ND | --/--/--/Q | <RL (J)/<RL (J)/<RL (J)/ND | --/--/--/Q | ND/ND/ND | --/--/-- | ND/ND/ND | --/--/-- | ND/ND/ND | --/--/-- | ND/ND/ND | --/--/-- |
| **Perfluorooctane sulfonamidoacetic acids** | **NEtFOSAA** | ND (RL: 30)/ND (RL: 30)/ND (RL: 30) | --/--/-- | <RL (J)/<RL (J)/<RL (J) | --/--/-- | 5.6/6.4/5.9 | 28.4/33.77/36.01 | ND (RL: 29)/<RL (J)/<RL (J)/ND | --/--/--/Q | <RL (J)/<RL (J)/<RL (J)/ND | --/--/--/Q | ND/ND/ND | --/--/-- | ND/ND/ND | --/--/-- | ND/ND/ND | --/--/-- | ND/ND/ND | --/--/-- |
|  | **NMeFOSAA** | ND (RL: 20)/ND (RL: 20)/ND (RL: 20) | --/--/-- | <RL (J)/<RL (J)/<RL (J) | --/--/-- | 3.2/3.8/4.1 | 16.23/20.05/25.02 | ND (RL: 19)/<RL (J)/<RL (J)/<RL (J) | --/--/--/-- | <RL (J)/ND/<RL (J)/ND | --/--/--/Q | ND/ND/ND | --/--/-- | ND/ND/ND | --/--/-- | ND/ND/ND | --/--/-- | ND/ND/ND | --/--/-- |
|  | **Units** | **AOF, EOF : µg F/L, ng F/g** | **mg F/run** | **AOF, EOF : µg F/L, ng F/g** | **mg F/run** | **AOF, EOF : µg F/L, ng F/g** | **mg F/run** | **AOF, EOF : µg F/L, ng F/g** | **mg F/run** | **AOF, EOF : µg F/L, ng F/g** | **mg F/run** | **AOF, EOF : µg F/L, ng F/g** | **mg F/run** | **AOF, EOF : µg F/L, ng F/g** | **mg F/run** | **AOF, EOF : µg F/L, ng F/g** | **mg F/run** | **AOF, EOF : µg F/L, ng F/g** | **mg F/run** |
| **TOF** | **AOF** | 20 (RL: 20)/21 (RL: 20)/<RL | 5318.69/5623.32/-- | NA/NA/NA | --/--/-- | NA/NA/NA | NA/NA/NA | <RL/ND (RL: 10)/ND (RL: 10)/ND (RL: 10) | --/--/--/-- | <RL/ND (RL: 10)/<RL/<RL | --/--/--/-- | NA/NA/NA | NA/NA/NA | NA/NA/NA | NA/NA/NA | NA/NA/NA | NA/NA/NA | NA/NA/NA | NA/NA/NA |
|  | **EOF** | NA/NA/NA | NA/NA/NA | ND/ND/ND | --/--/-- | 320/320/310 | 1622.78/1688.56/1891.82 | NA/NA/NA/NA | NA/NA/NA/NA | NA/NA/NA/NA | NA/NA/NA/NA | NA/NA/NA | NA/NA/NA | NA/NA/NA | NA/NA/NA | NA/NA/NA | NA/NA/NA | NA/NA/NA | NA/NA/NA |

## Flowrates Normalized to Plant Flow

| **Table** S-13**:** **Dryer Input Flowrates** | | | | | | |
| --- | --- | --- | --- | --- | --- | --- |
|  |  | **Thickened Solids (Liquid Fraction)** | **Thickened Solids (Solid Fraction)** | **Dryer Combustion Air** | **Cooling Water Supply (Process)** | **Cooling Water Supply (Potable)** |
|  | **Units:** | mg/hr | mg/hr | mg/hr | mg/hr | mg/hr |
| **PFAS Family** | **Acronym** | ***(R1/R2/R3)*** | ***(R1/R2/R3)*** | ***(R1/R2/R3)*** | ***(R1/R2/R3/R4)*** | ***(R1/R2/R3/R4)*** |
| **Cyclic PFAS** | **PFECHS** | NA/NA/NA | NA/NA/NA | --/--/-- | NA/NA/NA/NA | NA/NA/NA/NA |
| **Ether sulfonic acids** | **9Cl-PF3ONS** | --/--/-- | --/--/-- | --/--/-- | --/--/--/Q | --/--/--/Q |
|  | **11Cl-PF3OUdS** | --/--/-- | --/--/-- | --/--/-- | --/--/--/Q | --/--/--/Q |
| **Fluorotelomer carboxylic acids** | **3:3 FTCA** | NA/NA/NA | NA/NA/NA | --/--/-- | NA/NA/NA/NA | NA/NA/NA/NA |
|  | **5:3 FTCA** | NA/NA/NA | NA/NA/NA | --/--/-- | NA/NA/NA/NA | NA/NA/NA/NA |
|  | **6:2 FTCA** | NA/NA/NA | NA/NA/NA | --/--/-- | NA/NA/NA/NA | NA/NA/NA/NA |
|  | **7:3 FTCA** | NA/NA/NA | NA/NA/NA | --/--/-- | NA/NA/NA/NA | NA/NA/NA/NA |
|  | **8:2 FTCA** | NA/NA/NA | NA/NA/NA | --/--/-- | NA/NA/NA/NA | NA/NA/NA/NA |
|  | **10:2 FTCA** | NA/NA/NA | NA/NA/NA | --/--/-- | NA/NA/NA/NA | NA/NA/NA/NA |
| **Fluorotelomer sulfonic acids** | **4:2 FTS** | --/--/-- | --/--/-- | --/--/-- | --/--/--/Q | --/--/--/Q |
|  | **6:2 FTS** | --/--/-- | --/--/-- | --/--/-- | --/--/--/Q | --/--/--/Q |
|  | **8:2 FTS** | --/--/-- | --/--/-- | --/--/-- | --/--/--/Q | --/--/--/Q |
|  | **10:2 FTS** | --/--/-- | --/--/-- | --/--/-- | --/--/--/Q | --/--/--/Q |
| **Fluorotelomer unsaturated carboxylic acids** | **6:2 FTUCA** | NA/NA/NA | NA/NA/NA | --/--/-- | NA/NA/NA/NA | NA/NA/NA/NA |
|  | **8:2 FTUCA** | NA/NA/NA | NA/NA/NA | --/--/-- | NA/NA/NA/NA | NA/NA/NA/NA |
| **Per- and Polyfluoroether carboxylic acids** | **PFMPA** | NA/NA/NA | NA/NA/NA | --/--/-- | NA/NA/NA/NA | NA/NA/NA/NA |
|  | **PFMBA** | NA/NA/NA | NA/NA/NA | --/--/-- | NA/NA/NA/NA | NA/NA/NA/NA |
|  | **NFDHA** | NA/NA/NA | NA/NA/NA | --/--/-- | NA/NA/NA/NA | NA/NA/NA/NA |
|  | **HFPODA** | --/--/-- | --/--/-- | --/--/-- | --/--/--/Q | --/--/--/Q |
|  | **DONA** | --/--/-- | --/--/-- | --/--/-- | --/--/--/Q | --/--/--/Q |
| **Perfluoroalkyl carboxylic acids** | **PFBA** | --/--/-- | --/--/-- | --/--/-- | 0.79/0.72/0.78/Q | --/--/--/Q |
|  | **PFPeA** | --/--/-- | --/--/-- | --/--/-- | 2.37/2.37/2.48/Q | --/--/--/-- |
|  | **PFHxA** | --/--/-- | --/--/-- | --/--/-- | 3.27/2.82/2.93/Q | --/--/--/Q |
|  | **PFHpA** | --/--/-- | --/--/-- | --/--/-- | 0.9/0.85/0.87/Q | --/--/Q/-- |
|  | **PFOA** | --/--/-- | --/--/-- | --/--/-- | 1.81/1.69/1.69/Q | --/--/--/-- |
|  | **PFNA** | --/--/-- | --/--/-- | --/--/-- | Q/Q/--/-- | --/--/--/Q |
|  | **PFDA** | --/--/-- | --/--/36.73 | --/--/-- | 0.33/Q/0.23/-- | --/--/--/Q |
|  | **PFUnA** | --/--/-- | --/--/-- | --/--/-- | --/--/--/Q | --/--/--/Q |
|  | **PFDoA** | --/--/-- | --/--/-- | --/--/-- | Q/--/--/Q | --/--/--/Q |
|  | **PFTriA** | --/--/-- | --/--/-- | --/--/-- | --/--/--/Q | --/--/--/Q |
|  | **PFTeA** | --/--/-- | --/--/-- | --/--/-- | --/--/--/Q | --/--/--/Q |
|  | **PFHxDA** | --/--/-- | --/--/-- | --/--/-- | --/--/--/Q | --/--/--/Q |
|  | **PFODA** | --/--/-- | --/--/-- | --/--/-- | --/--/--/Q | --/--/--/Q |
| **Perfluoroalkyl sulfonic acids** | **PFEESA/PES** | NA/NA/NA | NA/NA/NA | --/--/-- | NA/NA/NA/NA | NA/NA/NA/NA |
|  | **PFBS** | --/--/-- | --/--/-- | --/--/-- | 0.54/0.53/0.45/Q | --/--/--/-- |
|  | **PFPeS** | --/--/-- | --/--/-- | --/--/-- | --/Q/--/Q | --/--/--/Q |
|  | **PFHxS** | --/--/-- | --/--/-- | --/--/-- | 0.3/0.32/0.33/Q | --/--/--/-- |
|  | **PFHpS** | --/--/-- | --/--/-- | --/--/-- | --/--/--/Q | --/--/--/Q |
|  | **PFOS** | --/--/-- | 43.64/51.31/81.81 | --/--/-- | 0.47/0.49/0.49/Q | --/--/--/-- |
|  | **PFNS** | --/--/-- | --/--/-- | --/--/-- | --/--/--/Q | --/--/--/Q |
|  | **PFDS** | --/--/-- | --/--/-- | --/--/-- | --/--/--/Q | --/--/--/Q |
|  | **PFDoS** | --/--/-- | --/--/-- | --/--/-- | --/--/--/Q | --/--/--/Q |
| **Perfluorooctane sulfonamide ethanols** | **NMeFOSE** | --/--/-- | --/--/-- | --/--/-- | --/--/--/Q | --/--/--/Q |
|  | **NEtFOSE** | --/--/-- | --/--/-- | --/--/-- | --/--/--/Q | --/--/--/Q |
| **Perfluorooctane sulfonamides** | **FOSA** | --/--/-- | --/--/-- | --/--/-- | 0.52/Q/--/-- | 0.02/0.01/0.01/Q |
|  | **NMeFOSA** | --/--/-- | --/--/-- | --/--/-- | --/--/--/Q | --/--/--/Q |
|  | **NEtFOSA** | --/--/-- | --/--/-- | --/--/-- | --/--/--/Q | --/--/--/Q |
| **Perfluorooctane sulfonamidoacetic acids** | **NMeFOSAA** | --/--/-- | --/--/-- | --/--/-- | --/--/--/Q | --/--/--/Q |
|  | **NEtFOSAA** | --/--/-- | --/--/-- | --/--/-- | --/--/--/Q | --/--/--/Q |

| **Table** S-14**:** **Dryer System Intermediate Flowrates** | | | | | | |
| --- | --- | --- | --- | --- | --- | --- |
|  |  | **Dewatered Solids** | **RTO Inlet (OTM-45 Front Half Wash)** | **RTO Inlet (OTM-45 Back Half Wash)** | **RTO Inlet (OTM-45 Impingers)** | **RTO Inlet (OTM-45 Breakthrough XAD Cartridge)** |
|  | **Units:** | mg/hr | mg/hr | mg/hr | mg/hr | mg/hr |
| **PFAS Family** | **Acronym** | ***(R1/R2/R3/R3 Duplicate)*** | ***(R1/R2/R3/R4)*** | ***(R1/R2/R3/R4)*** | ***(R1/R2/R3/R4)*** | ***(R1/R2/R3/R4)*** |
| **Cyclic PFAS** | **PFECHS** | NA/NA/NA/NA | --/--/--/-- | --/--/--/-- | --/--/--/-- | --/--/--/-- |
| **Ether sulfonic acids** | **9Cl-PF3ONS** | --/--/--/-- | --/--/--/-- | --/--/--/-- | --/--/--/-- | --/--/--/-- |
|  | **11Cl-PF3OUdS** | --/--/--/-- | --/--/--/-- | --/--/--/-- | --/--/--/-- | --/--/--/-- |
| **Fluorotelomer carboxylic acids** | **3:3 FTCA** | NA/NA/NA/NA | --/--/--/-- | --/--/--/-- | --/--/--/-- | --/--/--/-- |
|  | **5:3 FTCA** | NA/NA/NA/NA | 1.40E-02/--/--/-- | Q/Q/--/-- | 2.43E-01/3.31E-01/--/-- | Q/Q/Q/-- |
|  | **6:2 FTCA** | NA/NA/NA/NA | --/--/--/-- | 5.53E-02/3.68E-02/--/-- | --/--/--/-- | 4.40E-03/5.88E-03/1.04E-02/-- |
|  | **7:3 FTCA** | NA/NA/NA/NA | --/--/--/-- | 7.61E-03/--/--/-- | 7.67E-02/--/--/-- | --/--/--/-- |
|  | **8:2 FTCA** | NA/NA/NA/NA | --/--/--/-- | 9.24E-03/--/--/-- | --/--/--/-- | --/--/--/-- |
|  | **10:2 FTCA** | NA/NA/NA/NA | --/--/--/-- | Q/--/--/-- | --/--/--/-- | --/--/--/-- |
| **Fluorotelomer sulfonic acids** | **4:2 FTS** | --/--/--/-- | --/--/--/-- | --/--/--/-- | --/--/--/-- | --/--/--/-- |
|  | **6:2 FTS** | --/--/--/-- | --/--/--/-- | --/--/--/-- | --/--/--/-- | --/--/--/-- |
|  | **8:2 FTS** | --/--/--/-- | --/--/--/-- | 9.37E-03/--/--/-- | --/--/--/-- | --/--/--/-- |
|  | **10:2 FTS** | --/14.31/--/16.53 | --/--/--/-- | 3.92E-03/--/--/-- | --/--/--/-- | --/--/--/-- |
| **Fluorotelomer unsaturated carboxylic acids** | **6:2 FTUCA** | NA/NA/NA/NA | --/--/--/-- | 3.23E-02/--/--/-- | --/--/--/-- | 1.96E-03/2.96E-03/3.94E-03/-- |
|  | **8:2 FTUCA** | NA/NA/NA/NA | --/--/--/-- | 8.24E-03/--/--/-- | --/--/--/-- | --/--/--/-- |
| **Per- and Polyfluoroether carboxylic acids** | **PFMPA** | NA/NA/NA/NA | --/--/--/-- | --/--/--/-- | --/--/--/-- | --/--/--/-- |
|  | **PFMBA** | NA/NA/NA/NA | --/--/--/-- | --/--/--/-- | --/--/--/-- | --/--/--/-- |
|  | **NFDHA** | NA/NA/NA/NA | --/--/--/-- | --/--/--/-- | --/--/--/-- | --/--/--/-- |
|  | **HFPODA** | --/--/--/-- | Q/Q/Q/Q | Q/Q/Q/Q | Q/Q/Q/Q | --/3.20E-02/1.74E-01/5.24E-02 |
|  | **DONA** | --/--/--/-- | --/--/--/-- | --/--/--/-- | --/--/--/-- | --/--/--/-- |
| **Perfluoroalkyl carboxylic acids** | **PFBA** | --/--/--/-- | 6.07E-03/--/--/-- | 5.47E-02/--/--/-- | --/--/--/-- | --/--/--/-- |
|  | **PFPeA** | --/--/--/-- | 5.71E-03/--/--/-- | 4.51E-02/5.62E-02/--/-- | --/--/--/-- | 2.46E-03/3.56E-03/4.10E-03/-- |
|  | **PFHxA** | 7.12/9.37/7.81/8.72 | 1.06E-02/--/--/-- | 4.57E-01/5.47E-01/5.13E-01/-- | --/--/--/-- | 1.69E-02/2.74E-02/3.51E-02/-- |
|  | **PFHpA** | --/--/--/-- | --/--/--/-- | 2.31E-02/--/--/-- | --/--/--/-- | --/--/--/-- |
|  | **PFOA** | 8.01/6.24/6.01/8.26 | 8.09E-03/--/--/-- | Q/Q/--/-- | --/--/--/-- | 1.57E-03/2.35E-03/1.80E-03/1.87E-03 |
|  | **PFNA** | --/--/--/-- | --/--/--/-- | 8.64E-03/--/--/-- | --/--/--/-- | --/--/--/-- |
|  | **PFDA** | 12.71/14.31/15.03/16.53 | --/--/--/-- | 5.42E-02/4.71E-02/--/-- | --/--/--/-- | --/--/--/-- |
|  | **PFUnA** | --/--/--/-- | --/--/--/-- | --/--/--/-- | --/--/--/-- | --/--/--/-- |
|  | **PFDoA** | 4.7/5.72/6.46/6.16 | --/--/--/-- | 1.33E-02/--/--/-- | --/--/--/-- | --/--/--/-- |
|  | **PFTriA** | --/--/--/-- | --/--/--/-- | --/--/--/-- | --/--/--/-- | --/--/--/-- |
|  | **PFTeA** | --/--/--/-- | --/--/--/-- | --/--/--/-- | --/--/--/-- | --/--/--/-- |
|  | **PFHxDA** | --/--/--/-- | --/--/--/-- | --/--/--/-- | --/--/--/-- | --/--/--/-- |
|  | **PFODA** | --/--/--/-- | --/--/--/-- | --/--/--/-- | --/--/--/-- | --/--/--/-- |
| **Perfluoroalkyl sulfonic acids** | **PFEESA/PES** | NA/NA/NA/NA | --/--/--/-- | --/--/--/-- | --/--/--/-- | --/--/--/-- |
|  | **PFBS** | --/--/--/-- | --/--/--/-- | --/--/--/-- | --/--/--/-- | --/--/--/-- |
|  | **PFPeS** | --/--/--/-- | --/--/--/-- | --/--/--/-- | --/--/--/-- | Q/--/--/-- |
|  | **PFHxS** | --/--/--/-- | --/--/--/-- | --/--/--/-- | --/--/--/-- | --/--/--/-- |
|  | **PFHpS** | Q/--/--/-- | --/--/--/-- | --/--/--/-- | --/--/--/-- | --/--/--/-- |
|  | **PFOS** | 26.7/32.52/37.57/34.56 | --/--/--/-- | Q/--/--/-- | --/--/--/-- | --/--/--/-- |
|  | **PFNS** | --/--/--/-- | --/--/--/-- | --/--/--/-- | --/--/--/-- | --/--/--/-- |
|  | **PFDS** | --/4.81/4.96/-- | --/--/--/-- | --/--/--/-- | --/--/--/-- | --/--/--/-- |
|  | **PFDoS** | --/--/--/-- | Q/--/--/-- | --/--/--/-- | --/--/--/-- | --/--/--/-- |
| **Perfluorooctane sulfonamide ethanols** | **NMeFOSE** | --/--/--/-- | --/--/--/-- | --/Q/--/-- | --/--/--/-- | --/--/--/-- |
|  | **NEtFOSE** | --/--/--/-- | --/--/--/-- | 2.32E-02/Q/--/-- | --/--/--/-- | --/--/--/-- |
| **Perfluorooctane sulfonamides** | **FOSA** | --/--/--/-- | --/--/--/-- | --/--/--/-- | --/--/--/-- | --/--/--/-- |
|  | **NMeFOSA** | --/--/--/-- | --/--/--/-- | --/--/--/-- | --/--/--/-- | --/--/--/-- |
|  | **NEtFOSA** | --/--/--/-- | --/--/--/-- | --/--/--/-- | --/--/--/-- | --/--/--/-- |
| **Perfluorooctane sulfonamidoacetic acids** | **NMeFOSAA** | 15.25/15.61/19.53/18.03 | --/--/--/-- | --/--/--/-- | --/--/--/-- | --/--/--/-- |
|  | **NEtFOSAA** | 19.07/20.82/24.04/24.04 | --/--/--/-- | --/--/--/-- | --/--/--/-- | --/--/--/-- |

| **Table** S-15 **Dryer System Emissions Flowrates** | | | | | | | | | | |
| --- | --- | --- | --- | --- | --- | --- | --- | --- | --- | --- |
|  |  | **Centrate (Liquid Fraction)** | **Centrate (Solid Fraction** | **Dried Solids** | **Condenser Process Drain** | **Scrubber Process Drain** | **RTO Outlet (OTM-45 Front Half Wash)** | **RTO Outlet (OTM-45 Back Half Wash)** | **RTO Outlet (OTM-45 Impingers)** | **RTO Outlet (OTM-45 Breakthrough XAD Cartridge)** |
|  | **Units:** | mg/hr | mg/hr | mg/hr | mg/hr | mg/hr | mg/hr | mg/hr | mg/hr |  |
| **PFAS Family** | **Acronym** | ***(R1/R2/R3)*** | ***(R1/R2/R3)*** | ***(R1/R2/R3)*** | ***(R1/R2/R3/R4)*** | ***(R1/R2/R3/R4)*** | ***(R1/R2/R3)*** | ***(R1/R2/R3)*** | ***(R1/R2/R3)*** | ***(R1/R2/R3)*** |
| **Cyclic PFAS** | **PFECHS** | NA/NA/NA | NA/NA/NA | NA/NA/NA | NA/NA/NA/NA | NA/NA/NA/NA | --/--/-- | --/--/-- | --/--/-- | --/--/-- |
| **Ether sulfonic acids** | **9Cl-PF3ONS** | --/--/-- | --/--/-- | --/--/-- | --/--/--/Q | --/--/--/Q | --/--/-- | --/--/-- | --/--/-- | --/--/-- |
|  | **11Cl-PF3OUdS** | --/--/-- | --/--/-- | --/--/-- | --/--/--/Q | --/--/--/Q | --/--/-- | --/--/-- | --/--/-- | --/--/-- |
| **Fluorotelomer carboxylic acids** | **3:3 FTCA** | NA/NA/NA | NA/NA/NA | NA/NA/NA | NA/NA/NA/NA | NA/NA/NA/NA | --/--/-- | --/--/-- | --/--/-- | --/--/-- |
|  | **5:3 FTCA** | NA/NA/NA | NA/NA/NA | NA/NA/NA | NA/NA/NA/NA | NA/NA/NA/NA | --/--/-- | --/--/Q | --/--/-- | --/--/-- |
|  | **6:2 FTCA** | NA/NA/NA | NA/NA/NA | NA/NA/NA | NA/NA/NA/NA | NA/NA/NA/NA | --/--/-- | --/--/-- | --/--/-- | --/--/-- |
|  | **7:3 FTCA** | NA/NA/NA | NA/NA/NA | NA/NA/NA | NA/NA/NA/NA | NA/NA/NA/NA | --/--/-- | --/--/-- | --/--/-- | --/--/-- |
|  | **8:2 FTCA** | NA/NA/NA | NA/NA/NA | NA/NA/NA | NA/NA/NA/NA | NA/NA/NA/NA | --/--/-- | --/--/-- | --/--/-- | --/--/-- |
|  | **10:2 FTCA** | NA/NA/NA | NA/NA/NA | NA/NA/NA | NA/NA/NA/NA | NA/NA/NA/NA | --/--/-- | --/--/-- | --/--/-- | --/--/-- |
| **Fluorotelomer sulfonic acids** | **4:2 FTS** | --/--/-- | --/--/-- | --/--/-- | --/--/--/Q | --/--/--/Q | --/--/-- | --/--/-- | --/--/-- | --/--/-- |
|  | **6:2 FTS** | --/--/-- | --/--/-- | --/--/-- | --/--/--/Q | --/--/--/Q | --/--/-- | --/--/-- | --/--/-- | --/--/-- |
|  | **8:2 FTS** | --/--/-- | --/--/-- | --/--/-- | --/--/--/Q | --/--/--/Q | --/--/-- | --/--/-- | --/--/-- | --/--/-- |
|  | **10:2 FTS** | --/--/-- | --/--/-- | 2.97/3.62/4.05 | --/--/--/Q | --/--/--/Q | --/--/-- | --/--/-- | --/--/-- | --/--/-- |
| **Fluorotelomer unsaturated carboxylic acids** | **6:2 FTUCA** | NA/NA/NA | NA/NA/NA | NA/NA/NA | NA/NA/NA/NA | NA/NA/NA/NA | --/--/-- | --/--/-- | --/--/-- | --/--/-- |
|  | **8:2 FTUCA** | NA/NA/NA | NA/NA/NA | NA/NA/NA | NA/NA/NA/NA | NA/NA/NA/NA | --/--/-- | --/--/-- | --/--/-- | --/--/-- |
| **Per- and Polyfluoroether carboxylic acids** | **PFMPA** | NA/NA/NA | NA/NA/NA | NA/NA/NA | NA/NA/NA/NA | NA/NA/NA/NA | --/--/-- | --/--/-- | --/--/-- | --/--/-- |
|  | **PFMBA** | NA/NA/NA | NA/NA/NA | NA/NA/NA | NA/NA/NA/NA | NA/NA/NA/NA | --/--/-- | --/--/-- | --/--/-- | --/--/-- |
|  | **NFDHA** | NA/NA/NA | NA/NA/NA | NA/NA/NA | NA/NA/NA/NA | NA/NA/NA/NA | --/--/-- | --/--/-- | --/--/-- | --/--/-- |
|  | **HFPODA** | --/--/-- | --/--/-- | --/--/-- | --/--/--/Q | --/--/--/Q | Q/Q/Q | Q/Q/Q | Q/Q/Q | 4.61E-01/--/4.38E-01 |
|  | **DONA** | --/--/-- | --/--/-- | --/--/-- | --/--/--/Q | --/--/--/Q | --/--/-- | --/--/-- | --/--/-- | --/--/-- |
| **Perfluoroalkyl carboxylic acids** | **PFBA** | --/--/-- | --/--/-- | --/--/-- | --/0.95/1.07/Q | 0.02/0.02/0.02/Q | --/--/-- | --/--/-- | --/1.69E-02/-- | --/--/-- |
|  | **PFPeA** | 2.08/2.1/1.96 | --/--/-- | --/--/-- | 2.83/2.48/2.48/Q | 0.04/0.04/0.05/Q | --/--/-- | --/--/-- | --/7.67E-03/-- | --/--/-- |
|  | **PFHxA** | 3.96/4.39/4.05 | --/--/-- | 0.93/1.11/1.22 | 3.42/3.06/3.42/Q | 0.06/0.06/0.06/Q | --/--/-- | --/--/-- | --/--/-- | --/--/-- |
|  | **PFHpA** | 1.3/1.31/1.27 | --/--/-- | --/--/-- | --/1.05/1.06/Q | 0.01/0.02/0.02/Q | --/--/-- | --/--/-- | --/--/-- | --/--/-- |
|  | **PFOA** | 4.61/4.46/4.37 | --/--/-- | 1.61/1.68/1.95 | --/2.24/2.24/Q | 0.03/0.03/0.03/Q | --/--/-- | --/--/-- | --/1.09E-02/-- | --/--/1.47E-02 |
|  | **PFNA** | --/--/-- | --/--/-- | 1/1.05/1.22 | --/0.24/0.22/Q | --/--/--/-- | --/--/-- | --/--/-- | --/--/-- | --/--/-- |
|  | **PFDA** | --/--/-- | 8.09/5.37/2.39 | 5.69/5.82/6.45 | --/0.31/0.28/Q | --/--/0/-- | --/--/-- | --/--/-- | --/--/-- | --/--/-- |
|  | **PFUnA** | --/--/-- | --/--/-- | 1.24/1.55/1.5 | --/--/--/Q | --/--/--/Q | --/--/-- | Q/--/-- | --/--/-- | --/--/-- |
|  | **PFDoA** | --/--/-- | --/--/-- | 1.98/2.46/2.7 | --/--/--/Q | --/--/--/Q | --/--/-- | Q/--/Q | --/--/-- | --/--/-- |
|  | **PFTriA** | --/--/-- | --/--/-- | --/--/-- | --/--/--/Q | --/--/--/Q | --/--/-- | --/--/-- | --/--/-- | --/--/-- |
|  | **PFTeA** | --/--/-- | --/--/-- | --/--/-- | --/--/--/Q | --/--/--/Q | --/--/-- | --/--/-- | --/--/-- | --/--/-- |
|  | **PFHxDA** | --/--/-- | --/--/-- | --/--/-- | --/--/--/Q | --/--/--/Q | --/--/-- | --/--/-- | --/--/-- | --/--/-- |
|  | **PFODA** | --/--/-- | --/--/-- | --/--/-- | --/--/--/Q | --/--/--/Q | --/--/-- | --/--/-- | --/--/-- | --/--/-- |
| **Perfluoroalkyl sulfonic acids** | **PFEESA/PES** | NA/NA/NA | NA/NA/NA | NA/NA/NA | NA/NA/NA/NA | NA/NA/NA/NA | --/--/-- | --/--/-- | --/--/-- | --/--/-- |
|  | **PFBS** | --/--/-- | --/--/-- | --/--/-- | --/0.47/0.44/Q | 0.01/0.01/0.01/Q | --/--/-- | --/--/-- | --/--/-- | --/--/-- |
|  | **PFPeS** | --/--/-- | --/--/-- | --/--/-- | --/--/--/Q | --/--/--/-- | --/--/-- | --/--/-- | --/--/-- | --/--/-- |
|  | **PFHxS** | --/--/-- | --/--/-- | --/--/-- | --/0.35/0.35/Q | 0.01/0.01/0.01/Q | --/--/-- | --/--/-- | --/--/-- | --/--/-- |
|  | **PFHpS** | --/--/-- | --/--/-- | Q/--/Q | --/--/--/Q | --/--/--/Q | --/--/-- | --/--/-- | --/--/-- | --/--/-- |
|  | **PFOS** | 1.43/1.64/1.39 | 17.42/11.82/5.47 | 11.13/12.92/14.86 | --/0.85/0.84/Q | 0.01/0.01/0.01/Q | --/--/-- | --/--/-- | --/Q/-- | --/--/-- |
|  | **PFNS** | --/--/-- | --/--/-- | --/--/-- | --/--/--/Q | --/--/--/Q | --/--/-- | --/--/-- | --/--/-- | --/--/-- |
|  | **PFDS** | --/--/-- | --/--/Q | 1.48/1.68/1.65 | --/--/--/Q | --/--/--/Q | --/--/-- | --/--/-- | --/--/-- | --/--/-- |
|  | **PFDoS** | --/--/-- | --/--/-- | --/--/-- | --/--/--/Q | --/--/--/Q | --/--/-- | --/--/-- | --/--/-- | --/--/-- |
| **Perfluorooctane sulfonamide ethanols** | **NMeFOSE** | --/--/-- | --/--/-- | --/--/-- | --/--/--/Q | 0.01/0.01/0.01/Q | --/--/-- | --/--/-- | --/--/-- | --/--/-- |
|  | **NEtFOSE** | --/--/-- | --/--/-- | --/--/-- | --/--/--/Q | --/--/--/Q | --/--/-- | --/--/-- | --/--/-- | --/--/-- |
| **Perfluorooctane sulfonamides** | **FOSA** | --/--/-- | --/--/-- | --/--/-- | --/--/--/Q | --/--/--/Q | --/--/-- | --/--/-- | --/--/-- | --/--/-- |
|  | **NMeFOSA** | --/--/-- | --/--/-- | --/--/-- | --/--/--/Q | --/--/--/Q | --/--/-- | --/--/-- | --/--/-- | --/--/-- |
|  | **NEtFOSA** | --/--/-- | --/--/-- | --/--/-- | --/--/--/Q | --/--/--/Q | --/--/-- | --/--/-- | --/--/-- | --/--/-- |
| **Perfluorooctane sulfonamidoacetic acids** | **NMeFOSAA** | --/--/-- | --/--/-- | 3.96/4.91/6.15 | --/--/--/-- | --/--/--/Q | --/--/-- | --/--/-- | --/--/-- | --/--/-- |
|  | **NEtFOSAA** | --/--/-- | --/--/-- | 6.93/8.27/8.85 | --/--/--/Q | --/--/--/Q | --/--/-- | --/--/-- | --/--/-- | --/--/-- |

| **Table** S-16**:** **Summation of Targeted Analytes Molar Flows** | | | | | |
| --- | --- | --- | --- | --- | --- |
| **Sample Location** | **Sample Point** | **Run** | **Liquid Fraction** | **Solid Fraction** | **Total** |
|  |  |  | ***µmol/run*** | ***µmol/run*** | ***µmol/run*** |
| Thickened Solids | 1 | 1 | ND | 6081.71 | 6081.71 |
|  |  | 2 | ND | 7121.26 | 7121.26 |
|  |  | 3 | ND | 16829.92 | 16829.92 |
|  |  | AVG | ND | 10010.96 | 10010.96 |
| Centrate | 2 | 1 | 1931.03 | 3654.06 | 5585.09 |
|  |  | 2 | 1998.89 | 2451.96 | 4450.86 |
|  |  | 3 | 1868.96 | 1114.99 | 2983.95 |
|  |  | AVG | 1932.96 | 2407.00 | 4339.97 |
| Dewatered Solids | 3 | 1 | -- | -- | 12713.21 |
|  |  | 2 | -- | -- | 16820.60 |
|  |  | 3 | -- | -- | 16344.21 |
|  |  | AVG | -- | -- | 15292.67 |
| Combustion Air | 4 | 1 | -- | -- | 4.45E-05 |
|  |  | 2 | -- | -- | 4.17E-05 |
|  |  | 3 | -- | -- | 3.54E-05 |
|  |  | AVG | -- | -- | 4.05E-05 |
| Dried Solids | 5 | 1 | -- | -- | 5356.41 |
|  |  | 2 | -- | -- | 6158.18 |
|  |  | 3 | -- | -- | 6879.96 |
|  |  | AVG | -- | -- | 6131.52 |
| Cooling Water - Process | 6a | 1 | -- | -- | 1562.60 |
|  |  | 2 | -- | -- | 1336.26 |
|  |  | 3 | -- | -- | 1397.61 |
|  |  | AVG | -- | -- | 1432.16 |
| Cooling Water - Plant | 6b | 1 | -- | -- | 2.25 |
|  |  | 2 | -- | -- | 1.45 |
|  |  | 3 | -- | -- | 1.51 |
|  |  | AVG | -- | -- | 1.74 |
| Condenser Drain | 7 | 1 | -- | -- | 886.27 |
|  |  | 2 | -- | -- | 1651.70 |
|  |  | 3 | -- | -- | 1701.48 |
|  |  | AVG | -- | -- | 1413.15 |
| Scrubber Drain | 8 | 1 | -- | -- | 26.62 |
|  |  | 2 | -- | -- | 26.05 |
|  |  | 3 | -- | -- | 27.62 |
|  |  | AVG | -- | -- | 26.76 |
| Dryer Offgas | 9 | 1 | -- | -- | 2.17E-02 |
|  |  | 2 | -- | -- | 1.78E-02 |
|  |  | 3 | -- | -- | 1.73E-02 |
|  |  | AVG | -- | -- | 2.38E-02 |
| Exhaust Stack | 10 | 1 | -- | -- | 1.46E-03 |
|  |  | 2 | -- | -- | 1.17E-04 |
|  |  | 3 | -- | -- | 1.45E-03 |
|  |  | AVG | -- | -- | 1.55E-03 |

# QC Results

| **Table** S-17**:** **OTM-45 QC Results** | | | | | | | | | | | | | | | |
| --- | --- | --- | --- | --- | --- | --- | --- | --- | --- | --- | --- | --- | --- | --- | --- |
|  |  | **XAD Cartridge (Media Check)** | **Filter (Media Check)** | **Front Half Wash FBT** | **Back Half Wash FBT** | **Impingers 1,2&3** | **Breakthrough XAD FBT** | **Front Half Wash PBT** | **Back Half Wash PBT** | **Impingers 1,2&3 Condensate PBT** | **Breakthrough XAD-2 PBT** | **XAD-2 RB** | **Methanol/5% Ammonium Hydroxide RB** | **Filter RB** | **DI Water RB** |
| **Units** |  | **ng/Sample** | **ng/Sample** | **ng/Sample** | **ng/Sample** | **ng/Sample** | **ng/Sample** | **ng/Sample** | **ng/Sample** | **ng/Sample** | **ng/Sample** | **ng/Sample** | **ng/Sample** | **ng/Sample** | **ng/Sample** |
| **PFAS Family** | **Acronym** |  |  |  |  |  |  |  |  |  |  |  |  |  |  |
| **Cyclic PFAS** | **PFECHS** | ND | ND | ND | ND | ND | ND | ND | ND | ND | ND | ND | ND | ND | ND |
| **Ether sulfonic acids** | **9Cl-PF3ONS** | ND | ND | ND | ND | ND | ND | ND | ND | ND | ND | ND | ND | ND | ND |
|  | **11Cl-PF3OUdS** | *- *1 | ND | ND | *- *1 | *- *1 | *- *1 | ND | *- *1 | *- *1 | *- *1 | *- *1 | *- *1 | ND | *- *1 |
| **Fluorotelomer carboxylic acids** | **3:3 FTCA** | ND | ND | ND | ND | ND | ND | ND | ND | ND | ND | ND | ND | ND | ND |
|  | **5:3 FTCA** | *+ | ND | ND | *+ | ND | *+ | ND | *+ | ND | *+ | *+ | *+ | ND | ND |
|  | **6:2 FTCA** | ND | ND | ND | ND | ND | ND | ND | ND | ND | ND | ND | ND | ND | ND |
|  | **7:3 FTCA** | *+ | ND | ND | *+ | ND | *+ | ND | *+ | ND | *+ | *+ | *+ | ND | ND |
|  | **8:2 FTCA** | ND | ND | ND | ND | ND | ND | ND | ND | ND | ND | ND | ND | ND | ND |
|  | **10:2 FTCA** | ND | ND | ND | ND | ND | ND | ND | ND | ND | ND | ND | ND | ND | ND |
| **Fluorotelomer sulfonic acids** | **4:2 FTS** | ND | ND | ND | ND | ND | ND | ND | ND | ND | ND | ND | ND | ND | ND |
|  | **6:2 FTS** | ND | ND | ND | ND | ND | ND | ND | ND | ND | ND | ND | ND | ND | J |
|  | **8:2 FTS** | ND | ND | ND | ND | ND | ND | ND | ND | ND | ND | ND | ND | ND | ND |
|  | **10:2 FTS** | ND | ND | ND | ND | ND | ND | ND | ND | ND | ND | ND | ND | ND | ND |
| **Fluorotelomer unsaturated carboxylic acids** | **6:2 FTUCA** | ND | ND | ND | ND | ND | ND | ND | ND | ND | ND | ND | ND | ND | ND |
|  | **8:2 FTUCA** | ND | ND | ND | ND | ND | ND | ND | ND | ND | ND | ND | ND | ND | ND |
| **Per- and Polyfluoroether carboxylic acids** | **PFMPA** | ND | ND | ND | ND | ND | ND | ND | ND | ND | ND | ND | ND | ND | ND |
|  | **PFMBA** | ND | ND | ND | ND | ND | ND | ND | ND | ND | ND | ND | ND | ND | ND |
|  | **NFDHA** | ND | ND | ND | ND | ND | ND | ND | ND | ND | ND | ND | ND | ND | ND |
|  | **HFPODA** | ND | ND | 276 | 243 | 0.826 | ND | ND | ND | ND | ND | ND | ND | ND | ND |
|  | **DONA** | ND | ND | ND | ND | ND | ND | ND | ND | ND | ND | ND | ND | ND | ND |
| **Perfluoroalkyl carboxylic acids** | **PFBA** | ND | ND | ND | ND | J | CI | ND | CI | ND | CI | CI | ND | ND | ND |
|  | **PFPeA** | ND | ND | J | J | ND | ND | ND | ND | ND | ND | ND | ND | ND | ND |
|  | **PFHxA** | ND | ND | J | J | ND | ND | ND | ND | ND | ND | ND | ND | ND | ND |
|  | **PFHpA** | ND | ND | ND | ND | ND | ND | ND | ND | ND | ND | ND | ND | ND | ND |
|  | **PFOA** | ND | ND | ND | 7.57 | ND | ND | ND | ND | ND | ND | ND | ND | ND | ND |
|  | **PFNA** | ND | ND | ND | ND | ND | ND | ND | ND | ND | ND | ND | ND | ND | ND |
|  | **PFDA** | ND | ND | ND | ND | ND | ND | ND | ND | ND | ND | ND | ND | ND | ND |
|  | **PFUnA** | ND | ND | ND | ND | ND | ND | ND | ND | ND | ND | ND | ND | ND | ND |
|  | **PFDoA** | ND | ND | ND | ND | ND | ND | ND | ND | ND | ND | ND | ND | ND | ND |
|  | **PFTriA** | ND | ND | ND | ND | ND | ND | ND | ND | ND | ND | ND | ND | ND | ND |
|  | **PFTeA** | ND | ND | ND | ND | ND | ND | ND | ND | ND | ND | ND | ND | ND | ND |
|  | **PFHxDA** | ND | ND | ND | ND | ND | ND | ND | ND | ND | ND | ND | ND | ND | ND |
|  | **PFODA** | *- *1 | ND | ND | *- *1 | ND | *- *1 | ND | *- *1 | ND | *- *1 | *- *1 | *- *1 | ND | ND |
| **Perfluoroalkyl sulfonic acids** | **PFEESA/PES** | ND | ND | ND | ND | ND | ND | ND | ND | ND | ND | ND | ND | ND | ND |
|  | **PFBS** | ND | ND | ND | ND | ND | ND | ND | ND | ND | ND | ND | ND | ND | ND |
|  | **PFPeS** | ND | ND | ND | ND | ND | ND | ND | ND | ND | ND | ND | ND | ND | ND |
|  | **PFHxS** | ND | ND | ND | ND | ND | ND | ND | ND | ND | ND | ND | ND | ND | ND |
|  | **PFHpS** | ND | ND | ND | ND | ND | ND | ND | ND | ND | ND | ND | ND | ND | ND |
|  | **PFOS** | ND | ND | ND | ND | ND | ND | ND | ND | ND | ND | ND | ND | ND | ND |
|  | **PFNS** | ND | ND | ND | ND | ND | ND | ND | ND | ND | ND | ND | ND | ND | ND |
|  | **PFDS** | ND | ND | ND | ND | ND | ND | ND | ND | ND | ND | ND | ND | ND | ND |
|  | **PFDoS** | *- *1 | ND | ND | *- *1 | *- | *- *1 | ND | *- *1 | *- | *- *1 | *- *1 | *- *1 | ND | *- |
| **Perfluorooctane sulfonamide ethanols** | **NMeFOSE** | ND | ND | ND | ND | ND | ND | ND | ND | ND | ND | ND | ND | ND | ND |
|  | **NEtFOSE** | ND | ND | ND | ND | ND | ND | ND | ND | ND | ND | ND | ND | ND | ND |
| **Perfluorooctane sulfonamides** | **FOSA** | ND | ND | ND | ND | ND | ND | ND | ND | ND | ND | ND | ND | ND | ND |
|  | **NMeFOSA** | ND | ND | ND | ND | ND | ND | ND | ND | ND | ND | ND | ND | ND | ND |
|  | **NEtFOSA** | ND | ND | ND | ND | ND | ND | ND | ND | ND | ND | ND | ND | ND | ND |
| **Perfluorooctane sulfonamidoacetic acids** | **NMeFOSAA** | ND | ND | ND | ND | ND | ND | ND | ND | ND | ND | ND | ND | ND | ND |
|  | **NEtFOSAA** | ND | ND | ND | ND | ND | ND | ND | ND | ND | ND | ND | ND | ND | ND |

## Reporting Limits

| **Table** S-18**:** **Combustion Air Reporting Limits** | | | | |
| --- | --- | --- | --- | --- |
|  |  | **Run 1** | **Run 2** | **Run 3** |
|  | **Units:** | **ng/Sample** | **ng/Sample** | **ng/Sample** |
| **PFAS Family** | **Acronym** |  |  |  |
| **Cyclic PFAS** | **PFECHS** | 1 | 1 | 1 |
| **Ether sulfonic acids** | **9Cl-PF3ONS** | 1 | 1 | 1 |
|  | **11Cl-PF3OUdS** | 1 | 1 | 1 |
| **Fluorotelomer carboxylic acids** | **3:3 FTCA** | 1 | 1 | 1 |
|  | **5:3 FTCA** | 1 | 1 | 1 |
|  | **6:2 FTCA** | 1 | 1 | 1 |
|  | **7:3 FTCA** | 1 | 1 | 1 |
|  | **8:2 FTCA** | 1 | 1 | 1 |
|  | **10:2 FTCA** | 1 | 1 | 1 |
| **Fluorotelomer sulfonic acids** | **4:2 FTS** | 1 | 1 | 1 |
|  | **6:2 FTS** | 10 | 10 | 10 |
|  | **8:2 FTS** | 1 | 1 | 1 |
|  | **10:2 FTS** | 1 | 1 | 1 |
| **Fluorotelomer unsaturated carboxylic acids** | **6:2 FTUCA** | 1 | 1 | 1 |
|  | **8:2 FTUCA** | 1 | 1 | 1 |
| **Per- and Polyfluoroether carboxylic acids** | **PFMPA** | 1 | 1 | 1 |
|  | **PFMBA** | 1 | 1 | 1 |
|  | **NFDHA** | 1 | 1 | 1 |
|  | **HFPODA** | 20 | 20 | 20 |
|  | **DONA** | 2 | 2 | 2 |
| **Perfluoroalkyl carboxylic acids** | **PFBA** | 10 | 10 | 10 |
|  | **PFPeA** | 1 | 1 | 1 |
|  | **PFHxA** | 1 | 1 | 1 |
|  | **PFHpA** | 3 | 3 | 3 |
|  | **PFOA** | 1 | 1 | 1 |
|  | **PFNA** | 1 | 1 | 1 |
|  | **PFDA** | 1 | 1 | 1 |
|  | **PFUnA** | 1 | 1 | 1 |
|  | **PFDoA** | 1 | 1 | 1 |
|  | **PFTriA** | 1 | 1 | 1 |
|  | **PFTeA** | 1 | 1 | 1 |
|  | **PFHxDA** | 1 | 1 | 1 |
|  | **PFODA** | 1 | 1 | 1 |
| **Perfluoroalkyl sulfonic acids** | **PFEESA/PES** | 1 | 1 | 1 |
|  | **PFBS** | 1 | 1 | 1 |
|  | **PFPeS** | 1 | 1 | 1 |
|  | **PFHxS** | 1 | 1 | 1 |
|  | **PFHpS** | 1 | 1 | 1 |
|  | **PFOS** | 1 | 1 | 1 |
|  | **PFNS** | 1 | 1 | 1 |
|  | **PFDS** | 1 | 1 | 1 |
|  | **PFDoS** | 1 | 1 | 1 |
| **Perfluorooctane sulfonamide ethanols** | **NMeFOSE** | 20 | 20 | 20 |
|  | **NEtFOSE** | 1 | 1 | 1 |
| **Perfluorooctane sulfonamides** | **FOSA** | 1 | 1 | 1 |
|  | **NMeFOSA** | 1 | 1 | 1 |
|  | **NEtFOSA** | 1 | 1 | 1 |
| **Perfluorooctane sulfonamidoacetic acids** | **NMeFOSAA** | 1 | 1 | 1 |
|  | **NEtFOSAA** | 1 | 1 | 1 |

| **Table** S-19**:** **RTO Inlet Reporting Limits** | | | | | | | | | | | | | | | | | |
| --- | --- | --- | --- | --- | --- | --- | --- | --- | --- | --- | --- | --- | --- | --- | --- | --- | --- |
|  |  | **OTM-45 Front Half Wash** | | | | **OTM-45 Back Half Wash** | | | | **OTM-45 IMPINGERS** | | | | **OTM-45 Breakthrough XAD** | | | |
|  |  | **Run 1** | **Run 2** | **Run 3** | **Run 4** | **Run 1** | **Run 2** | **Run 3** | **Run 4** | **Run 1** | **Run 2** | **Run 3** | **Run 4** | **Run 1** | **Run 2** | **Run 3** | **Run 4** |
|  | **Units:** | **ng/Sample** | **ng/Sample** | **ng/Sample** | **ng/Sample** | **ng/Sample** | **ng/Sample** | **ng/Sample** | **ng/Sample** | **ng/Sample** | **ng/Sample** | **ng/Sample** | **ng/Sample** | **ng/Sample** | **ng/Sample** | **ng/Sample** | **ng/Sample** |
| **PFAS Family** | **Acronym** |  |  |  |  |  |  |  |  |  |  |  |  |  |  |  |  |
| **Cyclic PFAS** | **PFECHS** | 2 | 9.82 | 5 | 5 | 2 | 20 | 250 | 10 | 11.4 | 115 | 290 | 36.5 | 1 | 1 | 1 | 1 |
| **Ether sulfonic acids** | **9Cl-PF3ONS** | 2 | 9.82 | 5 | 5 | 2 | 20 | 250 | 10 | 11.4 | 115 | 290 | 36.5 | 1 | 1 | 1 | 1 |
|  | **11Cl-PF3OUdS** | 2 | 9.82 | 5 | 5 | 2 | 20 | 250 | 10 | 11.4 | 115 | 290 | 36.5 | 1 | 1 | 1 | 1 |
| **Fluorotelomer carboxylic acids** | **3:3 FTCA** | 2 | 9.82 | 5 | 5 | 2 | 20 | 250 | 10 | 11.4 | 115 | 290 | 36.5 | 1 | 1 | 1 | 1 |
|  | **5:3 FTCA** | 2 | 9.82 | 5 | 5 | 2 | 20 | 250 | 10 | 11.4 | 115 | 290 | 36.5 | 1 | 1 | 1 | 1 |
|  | **6:2 FTCA** | 2 | 9.82 | 5 | 5 | 2 | 20 | 250 | 10 | 11.4 | 115 | 290 | 36.5 | 1 | 1 | 1 | 1 |
|  | **7:3 FTCA** | 2 | 9.82 | 5 | 5 | 2 | 20 | 250 | 10 | 11.4 | 115 | 290 | 36.5 | 1 | 1 | 1 | 1 |
|  | **8:2 FTCA** | 2 | 9.82 | 5 | 5 | 2 | 20 | 250 | 10 | 11.4 | 115 | 290 | 36.5 | 1 | 1 | 1 | 1 |
|  | **10:2 FTCA** | 2 | 9.82 | 5 | 5 | 2 | 20 | 250 | 10 | 11.4 | 115 | 290 | 36.5 | 1 | 1 | 1 | 1 |
| **Fluorotelomer sulfonic acids** | **4:2 FTS** | 2 | 9.82 | 5 | 5 | 2 | 20 | 250 | 10 | 11.4 | 115 | 290 | 36.5 | 1 | 1 | 1 | 1 |
|  | **6:2 FTS** | 10 | 49.1 | 25 | 25 | 20 | 200 | 2500 | 100 | 11.4 | 115 | 290 | 36.5 | 10 | 10 | 10 | 10 |
|  | **8:2 FTS** | 2 | 9.82 | 5 | 5 | 2 | 20 | 250 | 10 | 11.4 | 115 | 290 | 36.5 | 1 | 1 | 1 | 1 |
|  | **10:2 FTS** | 2 | 9.82 | 5 | 5 | 2 | 20 | 250 | 10 | 11.4 | 115 | 290 | 36.5 | 1 | 1 | 1 | 1 |
| **Fluorotelomer unsaturated carboxylic acids** | **6:2 FTUCA** | 2 | 9.82 | 5 | 5 | 2 | 20 | 250 | 10 | 11.4 | 115 | 290 | 36.5 | 1 | 1 | 1 | 1 |
|  | **8:2 FTUCA** | 2 | 9.82 | 5 | 5 | 2 | 20 | 250 | 10 | 11.4 | 115 | 290 | 36.5 | 1 | 1 | 1 | 1 |
| **Per- and Polyfluoroether carboxylic acids** | **PFMPA** | 2 | 9.82 | 5 | 5 | 2 | 20 | 250 | 10 | 11.4 | 115 | 290 | 36.5 | 1 | 1 | 1 | 1 |
|  | **PFMBA** | 2 | 9.82 | 5 | 5 | 2 | 20 | 250 | 10 | 11.4 | 115 | 290 | 36.5 | 1 | 1 | 1 | 1 |
|  | **NFDHA** | 2 | 9.82 | 5 | 5 | 2 | 20 | 250 | 10 | 11.4 | 115 | 290 | 36.5 | 1 | 1 | 1 | 1 |
|  | **HFPODA** | 10 | 49.1 | 25 | 25 | 40 | 400 | 5000 | 200 | 11.4 | 115 | 290 | 36.5 | 20 | 20 | 20 | 20 |
|  | **DONA** | 2 | 9.82 | 5 | 5 | 4 | 40 | 500 | 20 | 11.4 | 115 | 290 | 36.5 | 2 | 2 | 2 | 2 |
| **Perfluoroalkyl carboxylic acids** | **PFBA** | 4 | 19.6 | 10 | 10 | 20 | 200 | 2500 | 100 | 11.4 | 115 | 290 | 36.5 | 10 | 10 | 10 | 10 |
|  | **PFPeA** | 2 | 9.82 | 5 | 5 | 2 | 20 | 250 | 10 | 11.4 | 115 | 290 | 36.5 | 1 | 1 | 1 | 1 |
|  | **PFHxA** | 2 | 9.82 | 5 | 5 | 2 | 20 | 250 | 10 | 11.4 | 115 | 290 | 36.5 | 1 | 1 | 1 | 1 |
|  | **PFHpA** | 2 | 9.82 | 5 | 5 | 6 | 60 | 750 | 30 | 11.4 | 115 | 290 | 36.5 | 3 | 3 | 3 | 3 |
|  | **PFOA** | 2 | 9.82 | 5 | 5 | 2 | 20 | 250 | 10 | 11.4 | 115 | 290 | 36.5 | 1 | 1 | 1 | 1 |
|  | **PFNA** | 2 | 9.82 | 5 | 5 | 2 | 20 | 250 | 10 | 11.4 | 115 | 290 | 36.5 | 1 | 1 | 1 | 1 |
|  | **PFDA** | 2 | 9.82 | 5 | 5 | 2 | 20 | 250 | 10 | 11.4 | 115 | 290 | 36.5 | 1 | 1 | 1 | 1 |
|  | **PFUnA** | 2 | 9.82 | 5 | 5 | 2 | 20 | 250 | 10 | 11.4 | 115 | 290 | 36.5 | 1 | 1 | 1 | 1 |
|  | **PFDoA** | 2 | 9.82 | 5 | 5 | 2 | 20 | 250 | 10 | 11.4 | 115 | 290 | 36.5 | 1 | 1 | 1 | 1 |
|  | **PFTriA** | 2 | 9.82 | 5 | 5 | 2 | 20 | 250 | 10 | 11.4 | 115 | 290 | 36.5 | 1 | 1 | 1 | 1 |
|  | **PFTeA** | 2 | 9.82 | 5 | 5 | 2 | 20 | 250 | 10 | 11.4 | 115 | 290 | 36.5 | 1 | 1 | 1 | 1 |
|  | **PFHxDA** | 2 | 9.82 | 5 | 5 | 2 | 20 | 250 | 10 | 11.4 | 115 | 290 | 36.5 | 1 | 1 | 1 | 1 |
|  | **PFODA** | 2 | 9.82 | 5 | 5 | 2 | 20 | 250 | 10 | 11.4 | 115 | 290 | 36.5 | 1 | 1 | 1 | 1 |
| **Perfluoroalkyl sulfonic acids** | **PFEESA/PES** | 2 | 9.82 | 5 | 5 | 2 | 20 | 250 | 10 | 11.4 | 115 | 290 | 36.5 | 1 | 1 | 1 | 1 |
|  | **PFBS** | 2 | 9.82 | 5 | 5 | 2 | 20 | 250 | 10 | 11.4 | 115 | 290 | 36.5 | 1 | 1 | 1 | 1 |
|  | **PFPeS** | 2 | 9.82 | 5 | 5 | 2 | 20 | 250 | 10 | 11.4 | 115 | 290 | 36.5 | 1 | 1 | 1 | 1 |
|  | **PFHxS** | 2 | 9.82 | 5 | 5 | 2 | 20 | 250 | 10 | 11.4 | 115 | 290 | 36.5 | 1 | 1 | 1 | 1 |
|  | **PFHpS** | 2 | 9.82 | 5 | 5 | 2 | 20 | 250 | 10 | 11.4 | 115 | 290 | 36.5 | 1 | 1 | 1 | 1 |
|  | **PFOS** | 2 | 9.82 | 5 | 5 | 2 | 20 | 250 | 10 | 11.4 | 115 | 290 | 36.5 | 1 | 1 | 1 | 1 |
|  | **PFNS** | 2 | 9.82 | 5 | 5 | 2 | 20 | 250 | 10 | 11.4 | 115 | 290 | 36.5 | 1 | 1 | 1 | 1 |
|  | **PFDS** | 2 | 9.82 | 5 | 5 | 2 | 20 | 250 | 10 | 11.4 | 115 | 290 | 36.5 | 1 | 1 | 1 | 1 |
|  | **PFDoS** | 2 | 9.82 | 5 | 5 | 2 | 20 | 250 | 10 | 11.4 | 115 | 290 | 36.5 | 1 | 1 | 1 | 1 |
| **Perfluorooctane sulfonamide ethanols** | **NMeFOSE** | 10 | 49.1 | 25 | 25 | 40 | 400^a^ | 5000 | 200 | 11.4 | 115 | 290 | 36.5 | 20 | 20 | 20 | 20 |
|  | **NEtFOSE** | 2 | 9.82 | 5 | 5 | 2 | 20^a^ | 250 | 10 | 11.4 | 115 | 290 | 36.5 | 1 | 1 | 1 | 1 |
| **Perfluorooctane sulfonamides** | **FOSA** | 2 | 9.82 | 5 | 5 | 2 | 20 | 250 | 10 | 11.4 | 115 | 290 | 36.5 | 1 | 1 | 1 | 1 |
|  | **NMeFOSA** | 2 | 9.82 | 5 | 5 | 2 | 20 | 250 | 10 | 11.4 | 115 | 290 | 36.5 | 1 | 1 | 1 | 1 |
|  | **NEtFOSA** | 2 | 9.82 | 5 | 5 | 2 | 20 | 250 | 10 | 11.4 | 115 | 290 | 36.5 | 1 | 1 | 1 | 1 |
| **Perfluorooctane sulfonamidoacetic acids** | **NMeFOSAA** | 2 | 9.82 | 5 | 5 | 2 | 20 | 250 | 10 | 11.4 | 115 | 290 | 36.5 | 1 | 1 | 1 | 1 |
|  | **NEtFOSAA** | 2 | 9.82 | 5 | 5 | 2 | 20 | 250 | 10 | 11.4 | 115 | 290 | 36.5 | 1 | 1 | 1 | 1 |

^a^ ­Units are ng/L

| **Table** S-20**: RTO Outlet Reporting Limits** | | | | | | | | | | | | | |
| --- | --- | --- | --- | --- | --- | --- | --- | --- | --- | --- | --- | --- | --- |
|  |  | **OTM-45 Front Half Wash** | | | **OTM-45 Back Half Wash** | | | **OTM-45 IMPINGERS** | | | **OTM-45 Breakthrough XAD** | | |
|  |  | **Run 1** | **Run 2** | **Run 3** | **Run 1** | **Run 2** | **Run 3** | **Run 1** | **Run 2** | **Run 3** | **Run 1** | **Run 2** | **Run 3** |
|  | **Units:** | **ng/Sample** | **ng/Sample** | **ng/Sample** | **ng/Sample** | **ng/Sample** | **ng/Sample** | **ng/Sample** | **ng/Sample** | **ng/Sample** | **ng/Sample** | **ng/Sample** | **ng/Sample** |
| **PFAS Family** | **Acronym** |  |  |  |  |  |  |  |  |  |  |  |  |
| **Cyclic PFAS** | **PFECHS** | 10 | 2 | 5 | 20 | 50 | 10 | 35 | 0.71 | 34.5 | 1 | 1 | 1 |
| **Ether sulfonic acids** | **9Cl-PF3ONS** | 10 | 2 | 5 | 20 | 50 | 10 | 35 | 0.71 | 34.5 | 1 | 1 | 1 |
|  | **11Cl-PF3OUdS** | 10 | 2 | 5 | 20 | 50 | 10 | 35 | 0.71 | 34.5 | 1 | 1 | 1 |
| **Fluorotelomer carboxylic acids** | **3:3 FTCA** | 10 | 2 | 5 | 20 | 50 | 10 | 35 | 0.71 | 34.5 | 1 | 1 | 1 |
|  | **5:3 FTCA** | 10 | 2 | 5 | 20 | 50 | 10 | 35 | 0.71 | 34.5 | 1 | 1 | 1 |
|  | **6:2 FTCA** | 10 | 2 | 5 | 20 | 50 | 10 | 35 | 0.71 | 34.5 | 1 | 1 | 1 |
|  | **7:3 FTCA** | 10 | 2 | 5 | 20 | 50 | 10 | 35 | 0.71 | 34.5 | 1 | 1 | 1 |
|  | **8:2 FTCA** | 10 | 2 | 5 | 20 | 50 | 10 | 35 | 0.71 | 34.5 | 1 | 1 | 1 |
|  | **10:2 FTCA** | 10 | 2 | 5 | 20 | 50 | 10 | 35 | 0.71 | 34.5 | 1 | 1 | 1 |
| **Fluorotelomer sulfonic acids** | **4:2 FTS** | 10 | 2 | 5 | 20 | 50 | 10 | 35 | 0.71 | 34.5 | 1 | 1 | 1 |
|  | **6:2 FTS** | 50 | 10 | 25 | 200 | 500 | 100 | 35 | 0.71 | 34.5 | 10 | 10 | 10 |
|  | **8:2 FTS** | 10 | 2 | 5 | 20 | 50 | 10 | 35 | 0.71 | 34.5 | 1 | 1 | 1 |
|  | **10:2 FTS** | 10 | 2 | 5 | 20 | 50 | 10 | 35 | 0.71 | 34.5 | 1 | 1 | 1 |
| **Fluorotelomer unsaturated carboxylic acids** | **6:2 FTUCA** | 10 | 2 | 5 | 20 | 50 | 10 | 35 | 0.71 | 34.5 | 1 | 1 | 1 |
|  | **8:2 FTUCA** | 10 | 2 | 5 | 20 | 50 | 10 | 35 | 0.71 | 34.5 | 1 | 1 | 1 |
| **Per- and Polyfluoroether carboxylic acids** | **PFMPA** | 10 | 2 | 5 | 20 | 50 | 10 | 35 | 0.71 | 34.5 | 1 | 1 | 1 |
|  | **PFMBA** | 10 | 2 | 5 | 20 | 50 | 10 | 35 | 0.71 | 34.5 | 1 | 1 | 1 |
|  | **NFDHA** | 10 | 2 | 5 | 20 | 50 | 10 | 35 | 0.71 | 34.5 | 1 | 1 | 1 |
|  | **HFPODA** | 50 | 10 | 25 | 400 | 1000 | 200 | 35 | 0.71 | 34.5 | 20 | 20 | 20 |
|  | **DONA** | 10 | 2 | 5 | 40 | 100 | 20 | 35 | 0.71 | 34.5 | 2 | 2 | 2 |
| **Perfluoroalkyl carboxylic acids** | **PFBA** | 20 | 4 | 10 | 200 | 500 | 100 | 35 | 0.71 | 34.5 | 10 | 10 | 10 |
|  | **PFPeA** | 10 | 2 | 5 | 20 | 50 | 10 | 35 | 0.71 | 34.5 | 1 | 1 | 1 |
|  | **PFHxA** | 10 | 2 | 5 | 20 | 50 | 10 | 35 | 0.71 | 34.5 | 1 | 1 | 1 |
|  | **PFHpA** | 10 | 2 | 5 | 60 | 150 | 30 | 35 | 0.71 | 34.5 | 3 | 3 | 3 |
|  | **PFOA** | 10 | 2 | 5 | 20 | 50 | 10 | 35 | 0.71 | 34.5 | 1 | 1 | 1 |
|  | **PFNA** | 10 | 2 | 5 | 20 | 50 | 10 | 35 | 0.71 | 34.5 | 1 | 1 | 1 |
|  | **PFDA** | 10 | 2 | 5 | 20 | 50 | 10 | 35 | 0.71 | 34.5 | 1 | 1 | 1 |
|  | **PFUnA** | 10 | 2 | 5 | 20 | 50 | 10 | 35 | 0.71 | 34.5 | 1 | 1 | 1 |
|  | **PFDoA** | 10 | 2 | 5 | 20 | 50 | 10 | 35 | 0.71 | 34.5 | 1 | 1 | 1 |
|  | **PFTriA** | 10 | 2 | 5 | 20 | 50 | 10 | 35 | 0.71 | 34.5 | 1 | 1 | 1 |
|  | **PFTeA** | 10 | 2 | 5 | 20 | 50 | 10 | 35 | 0.71 | 34.5 | 1 | 1 | 1 |
|  | **PFHxDA** | 10 | 2 | 5 | 20 | 50 | 10 | 35 | 0.71 | 34.5 | 1 | 1 | 1 |
|  | **PFODA** | 10 | 2 | 5 | 20 | 50 | 10 | 35 | 0.71 | 34.5 | 1 | 1 | 1 |
| **Perfluoroalkyl sulfonic acids** | **PFEESA/PES** | 10 | 2 | 5 | 20 | 50 | 10 | 35 | 0.71 | 34.5 | 1 | 1 | 1 |
|  | **PFBS** | 10 | 2 | 5 | 20 | 50 | 10 | 35 | 0.71 | 34.5 | 1 | 1 | 1 |
|  | **PFPeS** | 10 | 2 | 5 | 20 | 50 | 10 | 35 | 0.71 | 34.5 | 1 | 1 | 1 |
|  | **PFHxS** | 10 | 2 | 5 | 20 | 50 | 10 | 35 | 0.71 | 34.5 | 1 | 1 | 1 |
|  | **PFHpS** | 10 | 2 | 5 | 20 | 50 | 10 | 35 | 0.71 | 34.5 | 1 | 1 | 1 |
|  | **PFOS** | 10 | 2 | 5 | 20 | 50 | 10 | 35 | 0.71 | 34.5 | 1 | 1 | 1 |
|  | **PFNS** | 10 | 2 | 5 | 20 | 50 | 10 | 35 | 0.71 | 34.5 | 1 | 1 | 1 |
|  | **PFDS** | 10 | 2 | 5 | 20 | 50 | 10 | 35 | 0.71 | 34.5 | 1 | 1 | 1 |
|  | **PFDoS** | 10 | 2 | 5 | 20 | 50 | 10 | 35 | 0.71 | 34.5 | 1 | 1 | 1 |
| **Perfluorooctane sulfonamide ethanols** | **NMeFOSE** | 50 | 10 | 25 | 400 | 1000 | 200 | 35 | 0.71 | 34.5 | 20 | 20 | 20 |
|  | **NEtFOSE** | 10 | 2 | 5 | 20 | 50 | 10 | 35 | 0.71 | 34.5 | 1 | 1 | 1 |
| **Perfluorooctane sulfonamides** | **FOSA** | 10 | 2 | 5 | 20 | 50 | 10 | 35 | 0.71 | 34.5 | 1 | 1 | 1 |
|  | **NMeFOSA** | 10 | 2 | 5 | 20 | 50 | 10 | 35 | 0.71 | 34.5 | 1 | 1 | 1 |
|  | **NEtFOSA** | 10 | 2 | 5 | 20 | 50 | 10 | 35 | 0.71 | 34.5 | 1 | 1 | 1 |
| **Perfluorooctane sulfonamidoacetic acids** | **NMeFOSAA** | 10 | 2 | 5 | 20 | 50 | 10 | 35 | 0.71 | 34.5 | 1 | 1 | 1 |
|  | **NEtFOSAA** | 10 | 2 | 5 | 20 | 50 | 10 | 35 | 0.71 | 34.5 | 1 | 1 | 1 |

| **Table** S-21**:** **Synagro Inputs Reporting Limits** | | | | | | | | | | | | | | | |
| --- | --- | --- | --- | --- | --- | --- | --- | --- | --- | --- | --- | --- | --- | --- | --- |
|  |  | **Thickened Solids (Liquid Fraction)** | | | | | | **Cooling Water supply (plant)** | | | | **Cooling Water supply (Potable)** | | | |
|  |  | **Run 1** | **Run 2** | **Run 3** | **Run 1** | **Run 2** | **Run 3** | **Run 1** | **Run 2** | **Run 3** | **Run 4** | **Run 1** | **Run 2** | **Run 3** | **Run 4** |
|  | **Units:** | **ng/L** | **ng/L** | **ng/L** | **ng/g** | **ng/g** | **ng/g** | **ng/L** | **ng/L** | **ng/L** | **ng/L** | **ng/L** | **ng/L** | **ng/L** | **ng/L** |
| **PFAS Family** | **Acronym** |  |  |  |  |  |  |  |  |  |  |  |  |  |  |
| **Cyclic PFAS** | **PFECHS** | NA | NA | NA | NA | NA | NA | NA | NA | NA | NA | NA | NA | NA | NA |
| **Ether sulfonic acids** | **9Cl-PF3ONS** | 200 | 200 | 200 | 72 | 76 | 63 | 2 | 2 | 2 | 2 | 2 | 2 | 2 | 2 |
|  | **11Cl-PF3OUdS** | 200 | 200 | 200 | 21 | 23 | 19 | 2 | 2 | 2 | 2 | 2 | 2 | 2 | 2 |
| **Fluorotelomer carboxylic acids** | **3:3 FTCA** | NA | NA | NA | NA | NA | NA | NA | NA | NA | NA | NA | NA | NA | NA |
|  | **5:3 FTCA** | NA | NA | NA | NA | NA | NA | NA | NA | NA | NA | NA | NA | NA | NA |
|  | **6:2 FTCA** | NA | NA | NA | NA | NA | NA | NA | NA | NA | NA | NA | NA | NA | NA |
|  | **7:3 FTCA** | NA | NA | NA | NA | NA | NA | NA | NA | NA | NA | NA | NA | NA | NA |
|  | **8:2 FTCA** | NA | NA | NA | NA | NA | NA | NA | NA | NA | NA | NA | NA | NA | NA |
|  | **10:2 FTCA** | NA | NA | NA | NA | NA | NA | NA | NA | NA | NA | NA | NA | NA | NA |
| **Fluorotelomer sulfonic acids** | **4:2 FTS** | 200 | 200 | 200 | 72 | 76 | 63 | 2 | 2 | 2 | 2 | 2 | 2 | 2 | 2 |
|  | **6:2 FTS** | 500 | 500 | 500 | 72 | 76 | 63 | 6 | 5 | 4 | 5 | 2 | 2 | 2 | 2 |
|  | **8:2 FTS** | 300 | 300 | 300 | 110 | 110 | 94 | 4 | 3 | 3 | 3 | 2 | 2 | 2 | 2 |
|  | **10:2 FTS** | 500 | 500 | 500 | 72 | 76 | 63 | 6 | 5 | 4 | 5 | 2 | 2 | 2 | 2 |
| **Fluorotelomer unsaturated carboxylic acids** | **6:2 FTUCA** | NA | NA | NA | NA | NA | NA | NA | NA | NA | NA | NA | NA | NA | NA |
|  | **8:2 FTUCA** | NA | NA | NA | NA | NA | NA | NA | NA | NA | NA | NA | NA | NA | NA |
| **Per- and Polyfluoroether carboxylic acids** | **PFMPA** | NA | NA | NA | NA | NA | NA | NA | NA | NA | NA | NA | NA | NA | NA |
|  | **PFMBA** | NA | NA | NA | NA | NA | NA | NA | NA | NA | NA | NA | NA | NA | NA |
|  | **NFDHA** | NA | NA | NA | NA | NA | NA | NA | NA | NA | NA | NA | NA | NA | NA |
|  | **HFPODA** | 300 | 300 | 300 | 72 | 76 | 63 | 4 | 3 | 3 | 3 | 2 | 2 | 2 | 2 |
|  | **DONA** | 200 | 200 | 200 | 110 | 110 | 94 | 2 | 2 | 2 | 2 | 2 | 2 | 2 | 2 |
| **Perfluoroalkyl carboxylic acids** | **PFBA** | 500 | 500 | 500 | 72 | 76 | 63 | 5.9 | 5.1 | 4.5 | 5 | 2 | 2 | 2 | 2 |
|  | **PFPeA** | 200 | 200 | 200 | 21 | 23 | 19 | 2.3 | 2.1 | 1.8 | 2 | 1.8 | 1.8 | 2.5 | 1.9 |
|  | **PFHxA** | 200 | 200 | 200 | 21 | 23 | 19 | 2.3 | 2.1 | 1.8 | 2 | 1.8 | 1.8 | 2.5 | 1.9 |
|  | **PFHpA** | 200 | 200 | 200 | 21 | 23 | 19 | 2.3 | 2.1 | 1.8 | 2 | 1.8 | 1.8 | 2.5 | 1.9 |
|  | **PFOA** | 200 | 200 | 200 | 21 | 23 | 19 | 2.3 | 2.1 | 1.8 | 2 | 1.8 | 1.8 | 2.5 | 1.9 |
|  | **PFNA** | 200 | 200 | 200 | 21 | 23 | 19 | 2.3 | 2.1 | 1.8 | 2 | 1.8 | 2 | 2 | 2 |
|  | **PFDA** | 200 | 200 | 200 | 21 | 23 | 19 | 2.3 | 2.1 | 1.8 | 2 | 2 | 2 | 2 | 2 |
|  | **PFUnA** | 200 | 200 | 200 | 21 | 23 | 19 | 2 | 2 | 2 | 2 | 2 | 2 | 2 | 2 |
|  | **PFDoA** | 200 | 200 | 200 | 21 | 23 | 19 | 2.3 | 2 | 1.8 | 2 | 2 | 2 | 2 | 2 |
|  | **PFTriA** | 200 | 200 | 200 | 21 | 23 | 19 | 2 | 2 | 2 | 2 | 2 | 2 | 2 | 2 |
|  | **PFTeA** | 200 | 200 | 200 | 21 | 23 | 19 | 2 | 2 | 2 | 2 | 2 | 2 | 2 | 2 |
|  | **PFHxDA** | 300 | 300 | 300 | 21 | 23 | 19 | 4 | 3 | 3 | 3 | 2 | 2 | 2 | 2 |
|  | **PFODA** | 300 | 300 | 300 | 21 | 23 | 19 | 4 | 3 | 3 | 3 | 2 | 2 | 2 | 2 |
| **Perfluoroalkyl sulfonic acids** | **PFEESA/PES** | NA | NA | NA | NA | NA | NA | NA | NA | NA | NA | NA | NA | NA | NA |
|  | **PFBS** | 200 | 200 | 200 | 72 | 76 | 63 | 2.3 | 2.1 | 1.8 | 2 | 1.8 | 1.8 | 2.5 | 1.9 |
|  | **PFPeS** | 200 | 200 | 200 | 21 | 23 | 19 | 2 | 2.1 | 1.8 | 2 | 2 | 2 | 2 | 2 |
|  | **PFHxS** | 200 | 200 | 200 | 21 | 23 | 19 | 2.3 | 2.1 | 1.8 | 2 | 1.8 | 1.8 | 2.5 | 1.9 |
|  | **PFHpS** | 200 | 200 | 200 | 21 | 23 | 19 | 2 | 2 | 2 | 2 | 2 | 2 | 2.5 | 2 |
|  | **PFOS** | 200 | 200 | 200 | 21 | 23 | 19 | 2.3 | 2.1 | 1.8 | 2 | 1.8 | 1.8 | 2.5 | 1.9 |
|  | **PFNS** | 200 | 200 | 200 | 21 | 23 | 19 | 2 | 2 | 2 | 2 | 2 | 2 | 2.5 | 2 |
|  | **PFDS** | 200 | 200 | 200 | 21 | 23 | 19 | 2 | 2 | 2 | 2 | 2 | 2 | 2.5 | 2 |
|  | **PFDoS** | 300 | 300 | 300 | 72 | 76 | 63 | 4 | 3 | 3 | 3 | 2 | 2 | 2.5 | 2 |
| **Perfluorooctane sulfonamide ethanols** | **NMeFOSE** | 300 | 300 | 300 | 72 | 76 | 63 | 4 | 3 | 3 | 3 | 2 | 2 | 2 | 2 |
|  | **NEtFOSE** | 300 | 300 | 300 | 72 | 76 | 63 | 4 | 3 | 3 | 3 | 2 | 2 | 2 | 2 |
| **Perfluorooctane sulfonamides** | **FOSA** | 200 | 200 | 200 | 21 | 23 | 19 | 2.3 | 2.1 | 1.8 | 2 | 1.8 | 1.8 | 2.5 | 1.9 |
|  | **NMeFOSA** | 300 | 300 | 300 | 72 | 76 | 63 | 4 | 3 | 3 | 3 | 1.8 | 2 | 2 | 2 |
|  | **NEtFOSA** | 500 | 500 | 500 | 72 | 76 | 63 | 6 | 5 | 4 | 5 | 2 | 2 | 2 | 2 |
| **Perfluorooctane sulfonamidoacetic acids** | **NMeFOSAA** | 200 | 200 | 200 | 72 | 76 | 63 | 2 | 2 | 2 | 2 | 2 | 2 | 2 | 2 |
|  | **NEtFOSAA** | 300 | 300 | 300 | 72 | 76 | 63 | 4 | 3 | 3 | 3 | 2 | 2 | 2 | 2 |
|  | **Units:** |  |  |  |  |  |  |  |  |  |  |  |  |  |  |
| **AOF** | ***µg/L*** | 20 | 20 | 20 | NA | NA | NA | 10 | 10 | 10 | 10 | 2 | 2 | 2 | 2 |
| **EOF** | ***ng/g*** | NA | NA | NA | 11000 | 11000 | 9500 | NA | NA | NA | NA | NA | NA | NA | NA |

| **Table** S-22**:** **Synagro Intermediate Reporting Limits** | | | | | | | | | | | |
| --- | --- | --- | --- | --- | --- | --- | --- | --- | --- | --- | --- |
|  |  | **Centrate (Liquid Fraction)** | | | **Centrate (Solid Fraction)** | | | **Dewatered Solids** | | | |
|  |  | **Run 1** | **Run 2** | **Run 3** | **Run 1** | **Run 2** | **Run 3** | **Run 1** | **Run 2** | **Run 3** | **Run 3 (Duplicate)** |
|  | **Units:** | ng/L | **ng/L** | **ng/L** | **ng/g** | **ng/g** | **ng/g** | **ng/g** | **ng/g** | **ng/g** | **ng/g** |
| **PFAS Family** | **Acronym** |  |  |  |  |  |  |  |  |  |  |
| **Cyclic PFAS** | **PFECHS** | NA | NA | NA | NA | NA | NA | NA | NA | NA | NA |
| **Ether sulfonic acids** | **9Cl-PF3ONS** | 20 | 20 | 20 | 37 | 32 | 39 | 10 | 10 | 10 | 10 |
|  | **11Cl-PF3OUdS** | 20 | 20 | 20 | 11 | 10 | 12 | 3 | 3 | 3 | 3 |
| **Fluorotelomer carboxylic acids** | **3:3 FTCA** | NA | NA | NA | NA | NA | NA | NA | NA | NA | NA |
|  | **5:3 FTCA** | NA | NA | NA | NA | NA | NA | NA | NA | NA | NA |
|  | **6:2 FTCA** | NA | NA | NA | NA | NA | NA | NA | NA | NA | NA |
|  | **7:3 FTCA** | NA | NA | NA | NA | NA | NA | NA | NA | NA | NA |
|  | **8:2 FTCA** | NA | NA | NA | NA | NA | NA | NA | NA | NA | NA |
|  | **10:2 FTCA** | NA | NA | NA | NA | NA | NA | NA | NA | NA | NA |
| **Fluorotelomer sulfonic acids** | **4:2 FTS** | 20 | 20 | 20 | 37 | 32 | 39 | 10 | 10 | 10 | 10 |
|  | **6:2 FTS** | 50 | 50 | 49 | 37 | 32 | 39 | 10 | 10 | 10 | 10 |
|  | **8:2 FTS** | 30 | 30 | 30 | 56 | 47 | 58 | 14 | 15 | 14 | 14 |
|  | **10:2 FTS** | 50 | 50 | 49 | 37 | 32 | 39 | 9.6 | 9.7 | 9.5 | 9.6 |
| **Fluorotelomer unsaturated carboxylic acids** | **6:2 FTUCA** | NA | NA | NA | NA | NA | NA | NA | NA | NA | NA |
|  | **8:2 FTUCA** | NA | NA | NA | NA | NA | NA | NA | NA | NA | NA |
| **Per- and Polyfluoroether carboxylic acids** | **PFMPA** | NA | NA | NA | NA | NA | NA | NA | NA | NA | NA |
|  | **PFMBA** | NA | NA | NA | NA | NA | NA | NA | NA | NA | NA |
|  | **NFDHA** | NA | NA | NA | NA | NA | NA | NA | NA | NA | NA |
|  | **HFPODA** | 30 | 30 | 30 | 37 | 32 | 39 | 10 | 10 | 10 | 10 |
|  | **DONA** | 20 | 20 | 20 | 56 | 47 | 58 | 14 | 15 | 14 | 14 |
| **Perfluoroalkyl carboxylic acids** | **PFBA** | 50 | 50 | 49 | 37 | 32 | 39 | 10 | 10 | 10 | 10 |
|  | **PFPeA** | 20 | 20 | 20 | 11 | 10 | 12 | 2.9 | 3 | 3 | 3 |
|  | **PFHxA** | 20 | 20 | 20 | 11 | 9.5 | 12 | 2.9 | 2.9 | 2.9 | 2.9 |
|  | **PFHpA** | 20 | 20 | 20 | 11 | 10 | 12 | 3 | 3 | 3 | 3 |
|  | **PFOA** | 20 | 20 | 20 | 11 | 9.5 | 12 | 2.9 | 2.9 | 2.9 | 2.9 |
|  | **PFNA** | 20 | 20 | 20 | 11 | 10 | 12 | 2.9 | 2.9 | 2.9 | 2.9 |
|  | **PFDA** | 20 | 20 | 20 | 11 | 9.5 | 12 | 2.9 | 2.9 | 2.9 | 2.9 |
|  | **PFUnA** | 20 | 20 | 20 | 11 | 10 | 12 | 2.9 | 2.9 | 2.9 | 2.9 |
|  | **PFDoA** | 20 | 20 | 20 | 11 | 9.5 | 12 | 2.9 | 2.9 | 2.9 | 2.9 |
|  | **PFTriA** | 20 | 20 | 20 | 11 | 10 | 12 | 3 | 3 | 3 | 3 |
|  | **PFTeA** | 20 | 20 | 20 | 11 | 10 | 12 | 2.9 | 2.9 | 2.9 | 2.9 |
|  | **PFHxDA** | 30 | 30 | 30 | 11 | 10 | 12 | 3 | 3 | 3 | 3 |
|  | **PFODA** | 30 | 30 | 30 | 11 | 10 | 12 | 3 | 3 | 3 | 3 |
| **Perfluoroalkyl sulfonic acids** | **PFEESA/PES** | NA | NA | NA | NA | NA | NA | NA | NA | NA | NA |
|  | **PFBS** | 20 | 20 | 20 | 37 | 32 | 39 | 10 | 10 | 10 | 10 |
|  | **PFPeS** | 20 | 20 | 20 | 11 | 10 | 12 | 3 | 3 | 3 | 3 |
|  | **PFHxS** | 20 | 20 | 20 | 11 | 10 | 12 | 3 | 3 | 3 | 3 |
|  | **PFHpS** | 20 | 20 | 20 | 11 | 10 | 12 | 2.9 | 3 | 3 | 3 |
|  | **PFOS** | 20 | 20 | 20 | 11 | 9.5 | 12 | 2.9 | 2.9 | 2.9 | 2.9 |
|  | **PFNS** | 20 | 20 | 20 | 11 | 10 | 12 | 3 | 3 | 3 | 3 |
|  | **PFDS** | 20 | 20 | 20 | 11 | 9.5 | 12 | 2.9 | 2.9 | 2.9 | 2.9 |
|  | **PFDoS** | 30 | 30 | 30 | 37 | 32 | 39 | 10 | 9.7 | 9.5 | 9.6 |
| **Perfluorooctane sulfonamide ethanols** | **NMeFOSE** | 30 | 30 | 30 | 37 | 32 | 39 | 10 | 9.7 | 9.5 | 10 |
|  | **NEtFOSE** | 30 | 30 | 30 | 37 | 32 | 39 | 10 | 10 | 10 | 10 |
| **Perfluorooctane sulfonamides** | **FOSA** | 20 | 20 | 20 | 11 | 10 | 12 | 2.9 | 2.9 | 2.9 | 2.9 |
|  | **NMeFOSA** | 30 | 30 | 30 | 37 | 32 | 39 | 10 | 10 | 10 | 10 |
|  | **NEtFOSA** | 50 | 50 | 49 | 37 | 32 | 39 | 10 | 10 | 10 | 10 |
| **Perfluorooctane sulfonamidoacetic acids** | **NMeFOSAA** | 20 | 20 | 20 | 37 | 32 | 39 | 9.6 | 9.7 | 9.5 | 9.6 |
|  | **NEtFOSAA** | 30 | 30 | 30 | 37 | 32 | 39 | 9.6 | 9.7 | 9.5 | 9.6 |
|  | **Units:** |  |  |  |  |  |  |  |  |  |  |
| **AOF** | ***µg/L*** | 20 | 20 | 20 | NA | NA | NA | NA | NA | NA | NA |
| **EOF** | ***ng/g*** | NA | NA | NA | 5500 | 4500 | 5300 | 1400 | 1500 | 1500 | 1500 |

| **Table** S-23**:** **Synagro Emissions Reporting Limits** | | | | | | | | | | | | | | |
| --- | --- | --- | --- | --- | --- | --- | --- | --- | --- | --- | --- | --- | --- | --- |
|  |  | **Dried Solids** | | | | **Process Drain (Condenser)** | | | | | **Process Drain (Scrubber)** | | | |
|  |  | **Run 1** | **Run 2** | **Run 3** | **Run 3 (Duplicate)** | **Run 1** | **Run 2** | **Run 3** | **Run 4** | **Run 3 (Duplicate)** | **Run 1** | **Run 2** | **Run 3** | **Run 4** |
|  | **Units:** | **ng/g** | **ng/g** | **ng/g** | **ng/g** | **ng/L** | **ng/L** | **ng/L** | **ng/L** | **ng/L** | **ng/L** | **ng/L** | **ng/L** | **ng/L** |
| **PFAS Family** | **Acronym** | |  |  |  |  |  |  |  |  |  |  |  |  |
| **Cyclic PFAS** | **PFECHS** | NA | NA | NA | NA | NA | NA | NA | NA | NA | NA | NA | NA | NA |
| **Ether sulfonic acids** | **9Cl-PF3ONS** | 2 | 2 | 2 | 2 | 2 | 2 | 2 | 2 | 2 | 19 | 2 | 2 | 2 |
|  | **11Cl-PF3OUdS** | 1 | 1 | 1 | 1 | 2 | 2 | 2 | 2 | 2 | 19 | 2 | 2 | 2 |
| **Fluorotelomer carboxylic acids** | **3:3 FTCA** | NA | NA | NA | NA | NA | NA | NA | NA | NA | NA | NA | NA | NA |
|  | **5:3 FTCA** | NA | NA | NA | NA | NA | NA | NA | NA | NA | NA | NA | NA | NA |
|  | **6:2 FTCA** | NA | NA | NA | NA | NA | NA | NA | NA | NA | NA | NA | NA | NA |
|  | **7:3 FTCA** | NA | NA | NA | NA | NA | NA | NA | NA | NA | NA | NA | NA | NA |
|  | **8:2 FTCA** | NA | NA | NA | NA | NA | NA | NA | NA | NA | NA | NA | NA | NA |
|  | **10:2 FTCA** | NA | NA | NA | NA | NA | NA | NA | NA | NA | NA | NA | NA | NA |
| **Fluorotelomer sulfonic acids** | **4:2 FTS** | 2 | 2 | 2 | 2 | 2 | 2 | 2 | 2 | 2 | 19 | 2 | 2 | 2 |
|  | **6:2 FTS** | 2 | 2 | 2 | 2 | 4 | 4 | 5 | 5 | 5 | 48 | 4 | 5 | 4 |
|  | **8:2 FTS** | 3 | 3.3 | 3.3 | 3.3 | 3 | 3 | 3 | 3 | 3 | 29 | 3 | 3 | 3 |
|  | **10:2 FTS** | 2 | 2.2 | 2.2 | 2.2 | 4 | 4 | 5 | 5 | 5 | 48 | 4 | 5 | 4 |
| **Fluorotelomer unsaturated carboxylic acids** | **6:2 FTUCA** | NA | NA | NA | NA | NA | NA | NA | NA | NA | NA | NA | NA | NA |
|  | **8:2 FTUCA** | NA | NA | NA | NA | NA | NA | NA | NA | NA | NA | NA | NA | NA |
| **Per- and Polyfluoroether carboxylic acids** | **PFMPA** | NA | NA | NA | NA | NA | NA | NA | NA | NA | NA | NA | NA | NA |
|  | **PFMBA** | NA | NA | NA | NA | NA | NA | NA | NA | NA | NA | NA | NA | NA |
|  | **NFDHA** | NA | NA | NA | NA | NA | NA | NA | NA | NA | NA | NA | NA | NA |
|  | **HFPODA** | 2 | 2 | 2 | 2 | 3 | 3 | 3 | 3 | 3 | 29 | 3 | 3 | 3 |
|  | **DONA** | 3 | 3 | 3 | 3 | 2 | 2 | 2 | 2 | 2 | 19 | 2 | 2 | 2 |
| **Perfluoroalkyl carboxylic acids** | **PFBA** | 2 | 2 | 2 | 2 | 4.5 | 4.5 | 4.6 | 4.7 | 4.6 | 48 | 4.5 | 4.6 | 4.5 |
|  | **PFPeA** | 0.6 | 0.66 | 0.66 | 0.65 | 1.8 | 1.8 | 1.8 | 1.9 | 1.8 | 19 | 1.8 | 1.8 | 1.8 |
|  | **PFHxA** | 0.6 | 0.66 | 0.66 | 0.65 | 1.8 | 1.8 | 1.8 | 1.9 | 1.8 | 19 | 1.8 | 1.8 | 1.8 |
|  | **PFHpA** | 1 | 1 | 1 | 1 | 1.8 | 1.8 | 1.8 | 1.9 | 1.8 | 19 | 1.8 | 1.8 | 1.8 |
|  | **PFOA** | 0.6 | 0.66 | 0.66 | 0.65 | 1.8 | 1.8 | 1.8 | 1.9 | 1.8 | 19 | 1.8 | 1.8 | 1.8 |
|  | **PFNA** | 0.6 | 0.66 | 0.66 | 0.65 | 1.8 | 1.8 | 1.8 | 1.9 | 1.8 | 19 | 1.8 | 1.8 | 1.8 |
|  | **PFDA** | 0.6 | 0.66 | 0.66 | 0.65 | 1.8 | 1.8 | 1.8 | 1.9 | 1.8 | 19 | 1.8 | 1.8 | 1.8 |
|  | **PFUnA** | 0.6 | 0.66 | 0.66 | 0.65 | 2 | 2 | 2 | 2 | 2 | 19 | 2 | 2 | 2 |
|  | **PFDoA** | 0.6 | 0.66 | 0.66 | 0.65 | 1.8 | 2 | 2 | 2 | 2 | 19 | 2 | 2 | 2 |
|  | **PFTriA** | 1 | 1 | 1 | 1 | 2 | 2 | 2 | 2 | 2 | 19 | 2 | 2 | 2 |
|  | **PFTeA** | 0.6 | 0.66 | 0.66 | 0.65 | 2 | 2 | 2 | 2 | 2 | 19 | 2 | 2 | 2 |
|  | **PFHxDA** | 0.6 | 0.66 | 0.66 | 0.65 | 3 | 3 | 3 | 3 | 3 | 29 | 3 | 3 | 3 |
|  | **PFODA** | 1 | 1 | 1 | 1 | 3 | 3 | 3 | 3 | 3 | 29 | 3 | 3 | 3 |
| **Perfluoroalkyl sulfonic acids** | **PFEESA/PES** | NA | NA | NA | NA | NA | NA | NA | NA | NA | NA | NA | NA | NA |
|  | **PFBS** | 2 | 2 | 2 | 2 | 1.8 | 1.8 | 1.8 | 1.9 | 1.8 | 19 | 1.8 | 1.8 | 1.8 |
|  | **PFPeS** | 1 | 1 | 1 | 1 | 1.8 | 2 | 1.8 | 1.9 | 1.8 | 19 | 1.8 | 1.8 | 1.8 |
|  | **PFHxS** | 1 | 1 | 1 | 1 | 1.8 | 1.8 | 1.8 | 1.9 | 1.8 | 19 | 1.8 | 1.8 | 1.8 |
|  | **PFHpS** | 0.6 | 1 | 0.66 | 1 | 2 | 2 | 2 | 2 | 2 | 19 | 2 | 2 | 2 |
|  | **PFOS** | 0.6 | 0.66 | 0.66 | 0.65 | 1.8 | 1.8 | 1.8 | 1.9 | 1.8 | 19 | 1.8 | 1.8 | 1.8 |
|  | **PFNS** | 1 | 1 | 1 | 1 | 2 | 2 | 2 | 2 | 2 | 19 | 2 | 2 | 2 |
|  | **PFDS** | 0.6 | 0.66 | 0.66 | 0.65 | 2 | 2 | 2 | 2 | 2 | 19 | 2 | 2 | 2 |
|  | **PFDoS** | 2 | 2.2 | 2.2 | 2.2 | 3 | 3 | 3 | 3 | 3 | 29 | 3 | 3 | 3 |
| **Perfluorooctane sulfonamide ethanols** | **NMeFOSE** | 2 | 2.2 | 2 | 2 | 2.7 | 2.7 | 2.7 | 3 | 2.8 | 29 | 2.7 | 2.8 | 3 |
|  | **NEtFOSE** | 2 | 2.2 | 2.2 | 2.2 | 2.7 | 2.7 | 2.7 | 3 | 2.8 | 29 | 2.7 | 2.8 | 3 |
| **Perfluorooctane sulfonamides** | **FOSA** | 0.6 | 0.66 | 0.66 | 0.65 | 1.8 | 1.8 | 1.8 | 2 | 1.8 | 19 | 1.8 | 1.8 | 2 |
|  | **NMeFOSA** | 2 | 2 | 2 | 2 | 3 | 3 | 3 | 3 | 3 | 29 | 3 | 3 | 3 |
|  | **NEtFOSA** | 2 | 2 | 2 | 2 | 4 | 4 | 5 | 5 | 5 | 48 | 4 | 5 | 4 |
| **Perfluorooctane sulfonamidoacetic acids** | **NMeFOSAA** | 2 | 2.2 | 2.2 | 2.2 | 1.8 | 2 | 1.8 | 2 | 1.8 | 19 | 1.8 | 1.8 | 1.8 |
|  | **NEtFOSAA** | 2 | 2.2 | 2.2 | 2.2 | 2.7 | 2.7 | 2.7 | 3 | 2.8 | 29 | 2.7 | 2.8 | 3 |
|  | **Units:** |  |  |  |  |  |  |  |  |  |  |  |  |  |
| **AOF** | ***µg/L*** | NA | NA | NA | NA | 10 | 10 | 10 | 10 | 10 | 20 | 10 | 10 | 10 |
| **EOF** | ***ng/g*** | 320 | 320 | 310 | 340 | NA | NA | NA | NA | NA | NA | NA | NA | NA |

# References

ITRC (Interstate Technology & Regulatory Council). (2022a). Environmental Data Management Best 1620 Practices (EDMBP) Team Materials. Washington, D.C.: Interstate Technology & Regulatory Council, 1621 EDMBP Team. https://edm-1.itrcweb.org/ 1622

ITRC (Interstate Technology & Regulatory Council). (2022b). PFAS Technical and Regulatory Guidance 1623 Document and Fact Sheets PFAS-1. Section 11.3. Washington, D.C.: Interstate Technology & 1624 Regulatory Council, PFAS Team. https://pfas-1.itrcweb.org/

Joudan, S., Liu, R., D'eon, J.C., Mabury, S.A., 2020. Unique analytical considerations for laboratory studies identifying metabolic products of per- and polyfluoroalkyl substances (PFASs). TrAC - Trends Anal. Chem. 124, 115431. https://doi.org/10.1016/j.trac.2019.02.032.

Liu, Y., D’Agostino, L.A., Qu, G., Jiang, G., Martin, J.W., 2019. High-resolution mass spectrometry (HRMS) methods for nontarget discovery and characterization of poly- and per-fluoroalkyl substances (PFASs) in environmental and human samples. TrAC - Trends Anal. Chem. 121, 115420. https://doi.org/10.1016/j.trac.2019.02.021.

Mills, M.A., Impellitteri, C.A., Gillespie, A., 2020. Update on PFAS analytical methods. Emerging Contaminants Summit, Westminster, Colorado, March 10 - 11, 2020. https://www.contaminantssummit.com/ext/resources/images/Presentations_SponsBrochure/Marc-Mills.pdf.

Nakayama, S.F., Yoshikane, M., Onoda, Y., Nishihama, Y., Iwai-Shimada, M., Takagi, M., Kobayashi, Y., Isobe, T., 2019. Worldwide trends in tracing poly- and perfluoroalkyl substances (PFAS) in the environment. TrAC - Trends Anal. Chem. 121, 115410. https://doi.org/10.1016/j.trac.2019.02.011.

Trojanowicz, M., Koc, M., 2013. Recent developments in methods for analysis of perfluorinated persistent pollutants. Microchim. Acta 180, 957–971. https://doi.org/10.1007/s00604-013-1046-z.

USEPA. (1995). Development of compliance levels from analytical detection and quantitation levels, EPA 1658 Document Number: PB95-216321. Retrieved from 1659 https://nepis.epa.gov/Exe/ZyPDF.cgi?Dockey=9101IMKK.PDF

USEPA. (2017a). Method 2 — Determination of Stack Gas Velocity and Volumetric Flow Rate (Type S 1661 Pitot Tube). Retrieved from https://www.epa.gov/sites/default/files/2017- 1662 08/documents/method_2.pdf 1663

USEPA. (2017b). Method 3A — Determination of Oxygen and Carbon Dioxide Concentrations in 1664 Emissions from Stationary Sources (Instrumental Analyzer Procedure). Retrieved from 1665 https://www.epa.gov/sites/default/files/2017-08/documents/method_3a.pdf 1666

USEPA. (2017c). Method 4 — Determination of Moisture Content in Stack Gases. Retrieved from 1667 https://www.epa.gov/sites/default/files/2017-08/documents/method_4_0.pdf 1668

USEPA. (2017d). Appendix B—Definition and Procedure for the Determination of the Method Detection 1669 Limit—Revision 2. 40 C.F.R. § 136. Retrieved from https://www.ecfr.gov/current/title-40/chapter1670 I/subchapter-D/part-136#Appendix-B-to-Part-136

USEPA. (2022a). Draft Method 1621—Screening Method for the Determination of Adsorbable Organic 1682 Fluorine (AOF) – Aqueous Matrices by Combustion Ion Chromatography (CIC).

USEPA. (2022b). 3rd Draft Method 1633—Analysis of Per- and Polyfluoroalkyl Substances (PFAS) in 1686 Aqueous, Solid, Biosolids, and Tissue Samples by LC-MS/M. Retrieved from 1687 https://www.epa.gov/system/files/documents/2022- 1688 12/3rd%20Draft%20Method%201633%20December%202022%2012-20-22_508.pdf

Winchell, L. J., Wells, M. J. M., Ross, J. J., Kakar, F., Teymouri, A., Gonzalez, D. J., Dangtran, K., Bessler, S. M., Carlson, S., Almansa, X. F., Norton, J. W., & Bell, K. Y. (2024). Fate of perfluoroalkyl and polyfluoroalkyl substances (PFAS) through two full-scale wastewater sludge incinerators. Water Environment Research, 96(3). <https://doi.org/10.1002/wer.11009>
