# Supplementary figures and images for: Fate of PFAS Through a Biosolids Drum Dryer With Regenerative Thermal Oxidizer Emissions Control
Source: Water Environ Res. 2025 Oct 20;97(10):e70149. doi: 10.1002/wer.70149 (PMC12536579; doi:10.1002/wer.70149)

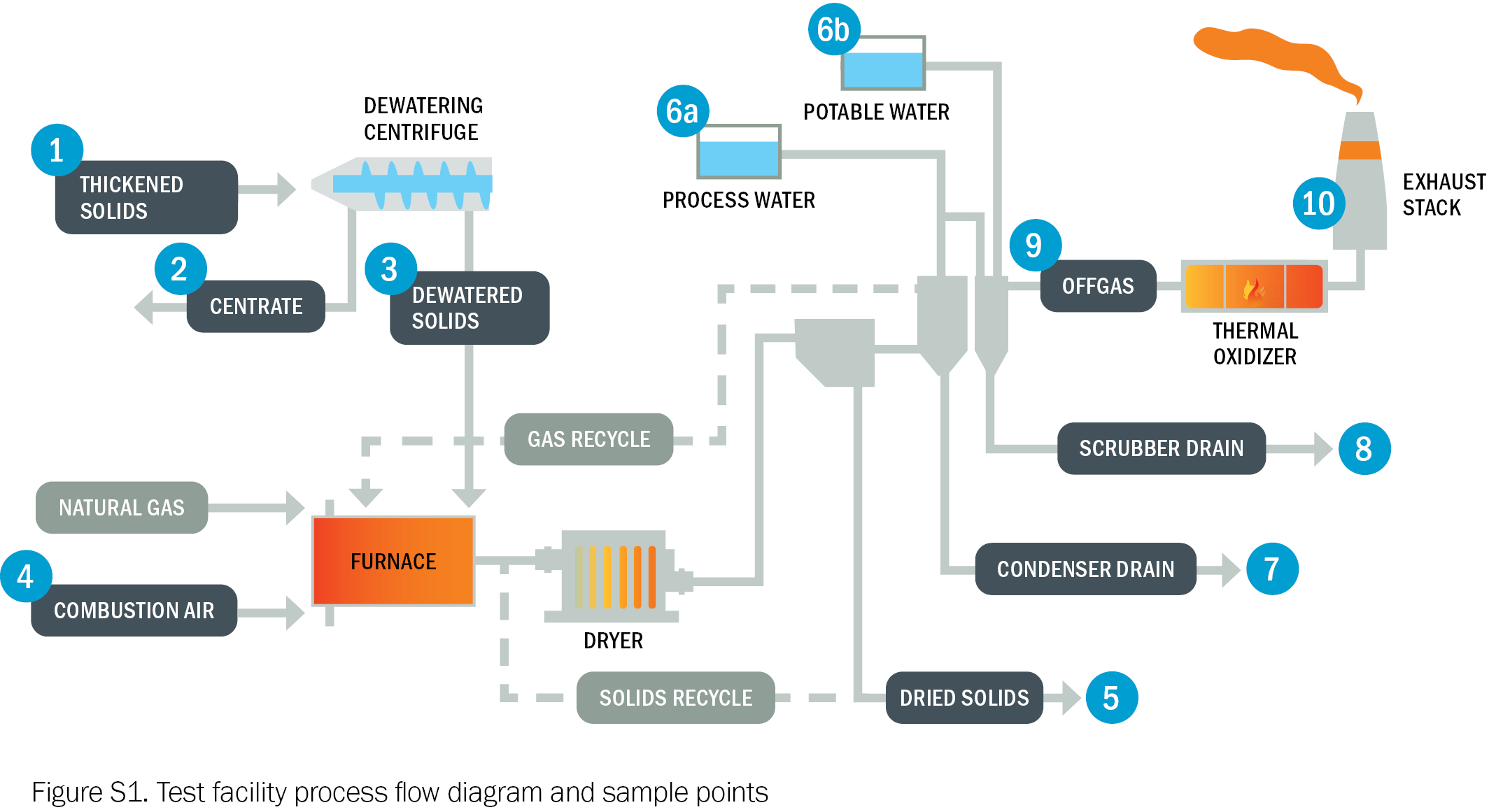

Supplement: Supplementary file 2 — Figure S1: Test facility process flow diagram and sample points. [file WER-97-e70149-s001.png]
